# A Two‐Protein Chemoreceptor Complex Regulates Oxygen Thresholds in Bacterial Magneto‐Aerotaxis

**DOI:** 10.1002/advs.202417315

**Published:** 2025-06-25

**Authors:** Julian Herz, Carina Weigel, Leonie Scheder, Raz Zarivach, Itay Algov, Yonatan Chemla, Felix Popp, Cornelius Riese, Mohammad A. Charsooghi, Lital Alfonta, Michael M. Meijler, Dirk Schüler, Damien Faivre, Daniel Pfeiffer

**Affiliations:** ^1^ Department of Microbiology University of Bayreuth 95447 Bayreuth Germany; ^2^ Department of Life Sciences and National Institute for Biotechnology in the Negev Ben‐Gurion University Beer Sheva 8410501 Israel; ^3^ Ilse Katz Institute for Nanoscale Science and Technology, Department of Life Sciences, and Department of Chemistry Ben‐Gurion University of the Negev Beer Sheva 8410501 Israel; ^4^ Department of Chemistry Ben‐Gurion University of the Negev Beer Sheva 8410501 Israel; ^5^ Department of Biology I Ludwig Maximilians University of Munich 82152 Planegg‐Martinsried Germany; ^6^ Department of Biomaterials Max Planck Institute of Colloids and Interfaces 14476 Potsdam Germany; ^7^ Aix‐Marseille Université CEA CNRS BIAM Saint Paul Lez Durance 13115 France; ^8^ Present address: Department of Cancer Biology Dana‐Farber Cancer Institute Boston MA 02215 USA; ^9^ Present address: Synthetic Biology Center Department of Biological Engineering Massachusetts Institute of Technology Cambridge MA 02139 USA

**Keywords:** aerotaxis, chemoreceptor, magnetosome, magnetospirillum, magnetotaxis

## Abstract

Bacteria in changing environments rely on motility and sensory mechanisms to locate optimal conditions. This process depends on specialized chemoreceptors to sense environmental stimuli. Exceptionally high numbers of chemoreceptor genes are present in magnetotactic bacteria (MTB), which combine magnetic alignment via intracellular magnetic nanoparticles (magnetosomes) and oxygen sensing for a unique navigation strategy toward low‐oxygen zones, called magneto‐aerotaxis. However, chemoreceptors for aerotaxis in MTB have not been experimentally identified. This study examines chemoreceptors in the model MTB *Magnetospirillum gryphiswaldense*. Gene deletion analysis shows that *M. gryphiswaldens*e relies on a complex and partly redundant set of chemoreceptors to sense oxygen. Within this diverse repertoire of chemoreceptors, a receptor formed by two interacting proteins is identified that plays a key role in aerotaxis. Interaction assays and microscopy confirm that both proteins interact within polar‐lateral regions in the cell. Moreover, genetic, biochemical, and motility experiments demonstrate that the chemoreceptor complex promotes a cellular response away from oxygen via the redox cofactor flavin adenine dinucleotide (FAD), independent of magnetic fields. These findings provide first insights into how MTB control oxygen sensing at the molecular level, shedding light on the mechanisms underlying bacterial navigation and highly complex chemosensory systems.

## Graphical abstract

Magnetotactic bacteria align with Earth's magnetic field, helping them migrate toward low‐oxygen environments. This study reveals how *Magnetospirillum gryphiswaldense* uses a specialized flavin adenine dinucleotide (FAD)‐dependent chemoreceptor, composed of two interacting proteins, for precise navigation in oxygen gradients. These findings enhance understanding of bacterial navigation and complex chemosensory systems, and may inspire synthetic biology applications like microrobotic design.

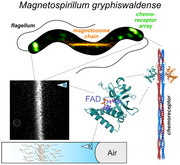

## 1 Introduction

Many bacteria demonstrate motility through flagella and use chemotaxis to navigate chemical gradients. A unique navigational mechanism that incorporates Earth's magnetic field is found in magnetotactic bacteria (MTB), which are abundant in many chemically stratified aquatic habitats. To align with the geomagnetic field, MTB employ magnetosomes – membrane‐bound magnetic iron crystals arranged in chains.^[^ ^1^ ^]^ Passive magnetic alignment is linked with flagella‐driven swimming and aerotaxis, a behavioral response guiding the search for optimal oxygen concentrations. Combined magneto‐aerotaxis is thought to facilitate migration to growth‐favoring micro‐ or anoxic zones.^[^ ^2^ ^]^

At the molecular level, temporal perception of environmental stimuli is mediated by chemoreceptors (methyl‐accepting chemotaxis proteins (MCPs)) within the chemosensory system to modulate flagellar motor function and coordinate behavior. Exceptionally high numbers of chemotaxis and motility genes are found in MTB,^[^ ^3^, ^4^, ^5^, ^6^, ^7^, ^8^, ^9^, ^10^ ^]^ with few documented exceptions.^[^ ^11^ ^]^ However, MCPs in MTB have remained poorly understood, as evidenced by few studies addressing their function.^[^ ^12^, ^13^, ^14^ ^]^ This is due to the uncultivability and genetic inaccessibility of most MTB,^[^ ^1^ ^]^ and the complexity of chemosensory systems in tractable strains.^[^ ^8^ ^]^ Likewise, individual MCPs are inadequately understood in many non‐MTB with a high number of MCPs.^[^ ^3^ ^]^

Different MCPs associated with oxygen‐sensing have been identified in non‐magnetotactic microorganisms.^[^ ^15^ ^]^ Protoglobin^[^ ^16^, ^17^ ^]^ and hemerythrin MCPs^[^ ^18^, ^19^ ^]^ bind oxygen directly via a heme iron complex and a non‐heme diiron center, respectively. In contrast, MCPs containing aerotaxis‐related PAS (Per‐Arnt‐Sim) domains (e.g., Aer in *Escherichia coli*) sense oxygen (and other electron acceptors) indirectly through metabolism‐dependent energy taxis via the respiratory chain and a PAS‐bound flavin adenine dinucleotide (FAD) cofactor.^[^ ^20^, ^21^, ^22^, ^23^ ^]^ A special bipartite energy taxis MCP found in *Campylobacter jejuni* consists of two proteins: a small cytoplasmic PAS‐domain sensory protein (CetB) and an inner membrane‐linked transducer (CetA).^[^ ^24^, ^25^ ^]^ Bipartite MCPs are encoded in diverse bacterial genomes, including genetically tractable *Magnetospirillum* strains^[^ ^26^ ^]^ (MTB inhabiting freshwater habitats), but their role in bacteria other than *C. jejuni* has remained obscure. Intriguingly, in *Magnetospirillum magneticum*, a bipartite MCP was suggested to function not in aerotaxis, but in a magnetoreceptive mechanism, allowing active sensing of Earth's magnetic field to maintain magnetic alignment.^[^ ^12^, ^13^, ^14^ ^]^

A closely related species, *Magnetospirillum gryphiswaldense* – a model for magnetosome formation and magneto‐aerotaxis – possesses one of the most complex chemosensory systems found in prokaryotes, including four chemotaxis operons (*cheOp1‐4*) and over 50 MCP genes, mostly encoded outside *cheOp1‐4*.^[^ ^8^ ^]^ Individual deletions of *cheOp1* and *cheOp4* resulted in loss of aerotactic behavior and impaired aerotaxis, respectively.^[^ ^8^, ^27^ ^]^ However, MCPs mediating aerotaxis in *M. gryphiswaldense* (or other MTB) had not been characterized. Thus, we aimed to identify oxygen‐sensing MCPs in *M. gryphiswaldense*. Via deletions of selected MCP genes, we provide evidence that *M. gryphiswaldense* possesses a sophisticated, partly redundant MCP repertoire. Furthermore, our findings do not support a role for any of the investigated MCPs in magnetorecepetion. Most importantly, we uncover an FAD‐dependent bipartite MCP as a key contributor to aerotaxis.

## 2 Results

### 2.1 Identification of a Bipartite Chemoreceptor in *M. gryphiswaldense*

Reassessment of the *M. gryphiswaldense* genome (GenBank acc. CP027526^[^ ^28^ ^]^) revealed 55 MCP‐encoding genes, in line with estimates of ≥56 and 54 MCPs from earlier versions of the genome^[^ ^8^ ^]^ and the Microbial Signal Transduction (MiST) database,^[^ ^29^ ^]^ respectively. These MCPs are characterized by diverse sensory modules also found in non‐magnetotactic prokaryotes.^[^ ^15^, ^30^, ^31^ ^]^ Among them, Cache (calcium channels and chemotaxis), PAS, and four‐helix bundle domains are the most common (Dataset S1, Supporting Information).

To identify relevant aerotaxis‐related MCPs, we deleted genes of MCPs featuring putative aero‐ or energy taxis‐related sensory domains (Figures S1 and S2, Supporting Information). The selected proteins include six PAS‐domain MCPs: two single chemoreceptors (Aer_1/2_) and four (CetBA‐type) bipartite systems encoded by gene pairs (Figures S1 and S3, Supporting Information). Additionally, we deleted two protoglobin (HemAT_1/2_) and two hemerythrin (MCP‐Hr_1/2_) MCPs, and the Cache‐domain MCP MSR1_02290, previously linked to a non‐magnetic Tn*5* mutant^[^ ^32^ ^]^ (Figure S1, Supporting Information). MCPs with extracellular Cache domains are known to mediate sensing of small organic molecules^[^ ^30^, ^31^, ^33^, ^34^, ^35^ ^]^ and energy taxis.^[^ ^36^ ^]^ Several selected MCPs carry additional sensory modules likely involved in small‐molecule recognition,^[^ ^30^, ^31^, ^37^ ^]^ such as a cyclic diguanylate (c‐di‐GMP)‐responsive PilZ domain (Figure S1 and Dataset S1, Supporting Information). In total, 11 MCP deletion strains were created (each lacking a single MCP), together representing one‐fifth of the total MCP complement, and a double deletion of the PAS‐domain MCPs Aer_1_ and Aer_2_.

**Figure 1:**
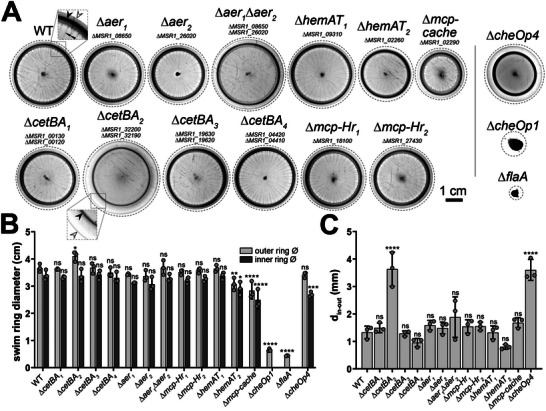
Swim halos of the WT and MCP deletion strains. Motile *M. gryphiswaldense* cells form macroscopic ring‐like patterns in soft agar through chemo‐/aerotaxis and growth‐dependent spreading, guided by emerging nutrient and electron acceptor gradients shaped by cellular metabolic activity and limited oxygen diffusion. A) Representative swim halos of the WT and MCP deletion strains are depicted. Dashed lines indicate cropped regions of images. Squares highlight twofold magnified areas. Morphological features, including the “inner ring,” the “outer ring,” and “spoke‐wheel‐like stripes,” are denoted by black, white, and black double arrowheads, respectively. For comparison, non‐motile (∆*flaA*) and non‐aerotactic (∆*cheOp1*) strains are shown. The ∆*flaA* strain lacks flagella due to deletion of the major flagellin gene *flaA*,^[^ ^40^ ^]^ while ∆*cheOp1* shows loss of aerotaxis due to deletion of the primary chemotaxis operon 1.^[^ ^8^ ^]^ The swim halo morphology of the ∆*cetBA_2_* strain partially resembles that of the ∆*cheOp4* strain (exhibiting impaired aerotaxis^[^ ^27^ ^]^), characterized by an increased distance between inner and outer swim rings and absence of spoke‐wheel‐like stripes (see Figure S4, Supporting Information, for a detailed comparison of both strains). Other stripe patterns are background. Note that the local geomagnetic field is insufficient to cause swim halo distortion (see Figure S24, Supporting Information). B) To quantify swim halo size, inner and outer swim ring diameters were measured. Larger values indicate faster cell spread in soft agar. C) To assess potential alterations in chemo‐/aerotactic behavior, the distance between inner and outer swim rings was calculated. Data in panels A‐C are from n  =  3 independent experiments. Dots in bar charts represent individual experiment results. Bars represent the mean, and error bars represent the standard deviation (SD). Statistical analysis involved one‐way analysis of variance (ANOVA) with Dunnett's multiple‐comparison test to compare mutants against the WT. Significance levels are indicated as follows: *, *p* < 0.05; **, *p* < 0.01; ***; *p* < 0.001; ****, *p* < 0.0001; not significant (ns), *P* ≥ 0.05.

**Figure 2:**
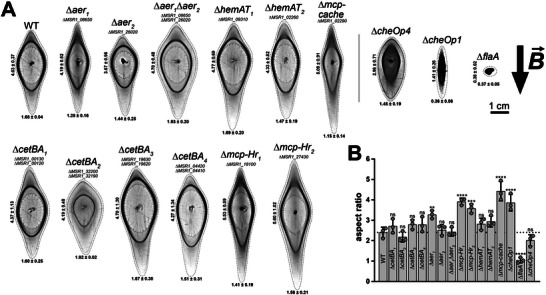
Swim halos of the WT and MCP deletion strains in a magnetic field. To assess magnetic alignment, swim halo distortion was quantified after incubating soft agar plates in a horizontally applied magnetic field. A) Representative swim halos formed in a homogeneous 600 µT magnetic field, with the field direction indicated by a black arrow, are shown. Dashed lines indicate cropped areas of images. The mean heights and widths (± SD) of the distorted outer rings (in centimeters) are provided. Note that stripes, apart from the regularly ordered stripes related to swim halos, are background. B) Aspect ratios (vertical/horizontal outer ring diameters) indicate the degree of magnetic alignment in motile strains. For the non‐motile ∆*flaA* strain, the aspect ratio is ≈1, similar to a non‐magnetic strain, as no halo forms. Dots represent individual experimental results, bars indicate the mean, and error bars show the SD. The dotted line represents WT behavior. Panels A‐B show data from *n* = 3 independent experiments. Statistical analysis was performed using one‐way ANOVA with Dunnett's multiple‐comparison test to compare mutants against the WT. **, *p* < 0.01; ***; *p* < 0.001; ****, *p* < 0.0001; ns, *P* ≥ 0.05.

Deletions in the chemosensory repertoire of non‐magnetotactic bacteria often alter their response to nutrient and electron acceptor gradients,^[^ ^23^, ^24^, ^38^ ^]^ prompting us to investigate *M. gryphiswaldense* MCP deletion strains using soft agar motility assays.^[^ ^39^ ^]^ In soft agar, motile cells spread in a chemo‐/aerotaxis‐ and growth‐dependent manner, forming ring‐like patterns, which are distorted under the influence of a horizontal magnetic field, allowing quantification of magnetic alignment.^[^ ^8^, ^27^, ^39^ ^]^ Unlike a non‐motile ∆*flaA* mutant^[^ ^40^ ^]^ and the non‐aerotactic ∆*cheOp1* strain,^[^ ^8^ ^]^ all MCP deletion mutants formed swim halos in soft agar, both without (**Figure** **1**) and with (**Figure** **2**) an applied magnetic field. Nonetheless, variations in swim halo morphology were observed, such as altered inner and outer swim ring appearance and spacing – patterns potentially linked to the ability of *M. gryphiswaldense* to utilize and exhibit tactic responses against the terminal electron acceptors nitrate and oxygen.^[^ ^8^, ^41^, ^42^ ^]^ For example, the ∆*mcp‐cache* and ∆*hemAT_2_* strains exhibited a reduced spreading efficiency (Figure 1). The most notable phenotype was observed in the ∆*cetBA_2_* strain, lacking a bipartite MCP. This strain formed a swim halo with a thinner inner ring, missing the spoke‐wheel‐like stripes seen in the wild type (WT) (Figure 1). Furthermore, akin to a ∆*cheOp4* strain^[^ ^8^ ^]^ that is impaired in aerotactic band formation,^[^ ^27^ ^]^ the ∆*cetBA_2_* strain displayed a 2.7‐fold increased distance between its inner and outer swim rings (Figure 1C; see Figures S4 and S5, Supporting Information for additional explanations). Except for a slight delay in the ∆*cetBA_2_* strain, MCP deletion strains showed WT‐like growth (Figure S6A, Supporting Information), suggesting that swim halo size differences are unrelated to growth.

Altered swim halo morphology of the ∆*cetBA_2_* mutant persisted under a magnetic field (Figure 2A). Specifically, while the distance between inner and outer swim rings increased in the direction of the magnetic field for all strains due to swim ring distortion, only the ∆*cetBA_2_* strain (and to a lesser extent the ∆*aer_1_* ∆*aer_2_* strain) showed a clear increase in distance perpendicular to the magnetic field. Importantly, none of the MCP mutants showed significantly impaired magnetic alignment, as confirmed by quantitative analysis of distorted swim halos (Figure 2B; refer to Figure S5, Supporting Information for measurement descriptions) and magnetic response (*C*_mag_) measurements (Figure S6B, Supporting Information), which assess magnetic field‐dependent changes in cell orientation and light scattering.^[^ ^43^ ^]^ Interestingly, some strains, such as the *MSR1_02290* deletion mutant – lacking a gene previously linked to a non‐magnetic phenotype^[^ ^32^ ^]^ – even showed improved alignment (see Supplemental Results & Discussion, Supporting Information). Consistent with the magnetic alignment phenotypes, transmission electron microscopy (TEM) revealed no obvious alterations in magnetosome chain formation in MCP deletion mutants (Figure S7, Supporting Information).

In summary, results suggested functional redundancy among MCPs, as no single deletion caused complete loss or severe impairment in swim halo formation. Nevertheless, altered swim halo morphology in some mutants indicated possible defects in aero‐ or chemotaxis. Given the conspicuous phenotype of the ∆*cetBA_2_* mutant, we focused subsequent analyses on this bipartite MCP system.

### 2.2 Characterization of the *cetBA_2_* Gene Locus

**Figure 3:**
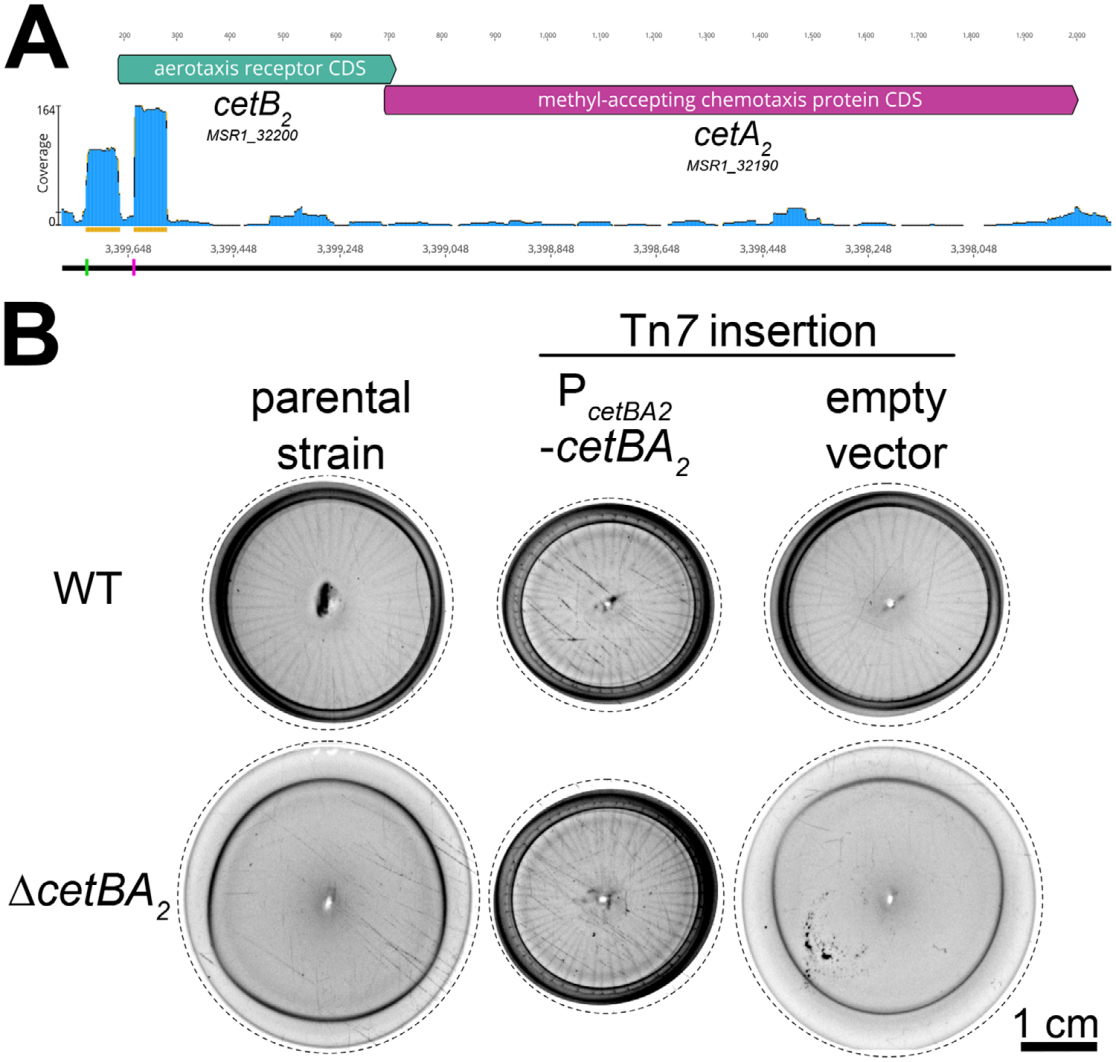
Genomic organization of the *cetBA_2_* locus. A) *cetA_2_* and *cetB_2_* are overlapping genes. Primary (green) and internal transcription start sites (magenta), identified by Cappable‐seq,^[^ ^45^ ^]^ are shown. Top numbers indicate base pairs; bottom numbers show genome positions. B) Expression of *cetBA_2_* in the ∆*cetBA_2_* strain restores a swim halo morphology closely resembling the WT. Shown are representative swim halos of the WT and ∆*cetBA_2_* strains carrying either an integrative Tn*7*‐based *cetBA_2_* expression cassette (including the putative native *cetBA_2_* promoter) or an empty vector in comparison to the parental strains. Three Tn*7* insertion mutants (n  =  3) per strain and construct were analyzed. Dashed lines indicate where images were cropped. Stripe patterns, other than the regularly ordered stripes of swim halos, are background.

In *M. gryphiswaldense*, the *cetBA_2_* locus comprises two overlapping genes, *cetB_2_* and *cetA_2_* (**Figure** **3**A; Figure S3, Supporting Information). A similar genetic organization is found for three other bipartite MCPs in *M. gryphiswaldense*, designated CetBA_1_, CetBA_3_, and CetBA_4_ (Figures S1 and S3, Supporting Information). A fifth CetB paralog (CetB_5_) encoded next to a CheY paralog lacks a corresponding CetA partner (Figure S3, Supporting Information). CetB and CetA paralogs share mean amino acid identities of 54.8% and 28.4% (Table S1, Supporting Information), respectively. Based on a proteome dataset,^[^ ^44^ ^]^ CetA_2_ is one of the most abundant MCPs in *M. gryphiswaldense* (Dataset S1, Supporting Information). RNA sequencing data^[^ ^45^ ^]^ suggests that *cetB_2_* and *cetA_2_* are cotranscribed from the same promoter (Figure 3A). Reintroducing *cetBA_2_* with its promoter into the ∆*cetBA_2_* strain restored WT swim halo morphology (Figure 3B), confirming the ∆*cetBA_2_* phenotype arises from *cetBA_2_* deletion. Moreover, individual deletions of *cetB_2_* and *cetA_2_* phenocopied the ∆*cetBA_2_* strain (Figure S8, Supporting Information), indicating a functional association between CetB_2_ and CetA_2_. Additionally, this observation implies that other CetB and CetA paralogs cannot serve as substitutes for CetB_2_ and CetA_2_, potentially due to interaction specificity or insufficient expression levels (as evidenced by proteomic data^[^ ^44^ ^]^ failing to detect CetB_3_ and CetB_5_; Dataset S1, Supporting Information). The ∆*cetB_2_* and ∆*cetA_2_* strains were successfully complemented by reintroducing the respective deleted gene (Figure S9, Supporting Information), confirming that functionality of the *cetB_2_* and *cetA_2_* genes was retained in the ∆*cetA_2_* and ∆*cetB_2_* strains, respectively.

In conclusion, the genetic context and deletion phenotypes of *cetB_2_* and *cetA_2_* suggested a functional link between the encoded proteins, motivating further investigation.

### 2.3 CetB_2_ and CetA_2_ Mediate an Oxygen Repellent Response Independent of Magnetic Fields

*M. gryphiswaldense* forms sharp aerotactic bands at low micromolar oxygen concentrations, reflecting microaerophilic behavior likely as part of a broader, metabolism‐driven energy taxis that includes additional terminal electron acceptors such as nitrate.^[^ ^8^, ^40^, ^41^, ^42^, ^46^ ^]^ Soft agar motility assays with varying oxygen and nitrate levels support that both electron acceptors influence the tactic behavior of *M. gryphiswaldense* and imply a role for CetB_2_ and CetA_2_ in sensing both (Figure S10, Supporting Information). Additionally, ∆*cetBA_2_* cells showed polar North‐ or South‐seeking behavior when grown in oxygen gradients superimposed with a magnetic field aligned parallel or antiparallel to the oxygen gradient (Figure S11, Supporting Information), respectively, suggesting their aerotaxis is partially impaired rather than completely lost.

**Figure 4:**
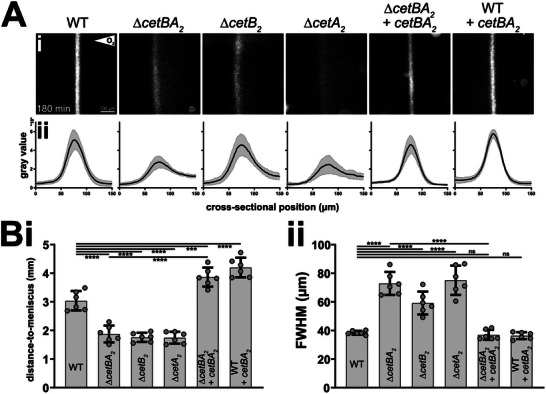
Deletion of *cetBA_2_* impairs aerotaxis. Ai) Examination of aerotactic band formation in flat glass microcapillaries, illustrated through dark‐field microscopy images captured after 180 min in a zero field. The microscopic scale and orientation of the oxygen gradient are indicated in the first panel. Aii) Averaged band intensity profiles with shaded areas representing 95% confidence intervals (CI). Bi) Band distance to the air‐liquid interface (meniscus) and Bii) full width at half maximum (FWHM) after 180 min. Panels A and B display data from n  =  6 microcapillary experiments. In panel B, dots represent individual experimental results, bars show the mean, and error bars depict the SD. Statistical analysis was conducted using one‐way ANOVA with Sidak's multiple comparison post‐test; ***, *P* < 0.001; ****, *P* < 0.0001; ns, *P* ≥ 0.05. The following strains were analyzed: the WT, a co‐deletion strain of *cetB_2_* and *cetA_2_*, single deletions of *cetB_2_* and *cetA_2_*, and WT and ∆*cetBA_2_* strains harboring a Tn*7*‐based *cetBA_2_* expression cassette with the native promoter.

To study the impact of CetB_2_ and CetA_2_ on aerotaxis, we conducted microcapillary assays to analyze aerotactic band formation. Aerotactic band formation at a defined distance from the air‐liquid interface in a microcapillary filled with cell suspension results from cells swimming toward a preferred microaerophilic zone within an oxygen gradient (Movie S1, Supporting Information), formed by limited oxygen diffusion and consumption via cellular respiration.^[^ ^8^, ^40^, ^41^, ^42^, ^46^ ^]^ Experiments were first performed in a zero field to rule out magnetic field effects on aerotactic band formation,^[^ ^46^ ^]^ utilizing a microscope equipped with coils allowing to apply defined magnetic fields.^[^ ^39^ ^]^ Consistent with Bennet et al.,^[^ ^46^ ^]^ the WT formed a sharp aerotactic band (**Figure** **4**A) ≈3 mm from the meniscus after 180 min (Figure 4B‐i). Strains lacking *cetB_2_* and/or *cetA_2_* showed no detectable delay in the onset of aerotactic band formation (Figure S12, Supporting Information). However, unlike the WT, these strains formed bands closer to the air‐liquid interface (≈1.8 mm after 180 min; Figure 4B‐i) and showed distortion toward higher oxygen concentrations (Figure 4A), with up to a twofold increase in band width (Figure 4B‐ii). Tracking of cells near the aerotactic band revealed decreased swimming speeds in strains lacking *cetB_2_* and/or *cetA_2_*, particularly in hyperoxic regions (Figure S13B and Table S2A, Supporting Information). However, this is likely due to the diffuse aerotactic bands formed by these strains, causing more cell collisions, as opposed to impaired motility (see Supplemental Results & Discussion, Supporting Information).

Reintroducing *cetBA_2_* into the ∆*cetBA_2_* strain restored a WT‐like band morphology (Figure 4; Figure S12 and Movie S2, Supporting Information). Moreover, although band formation was detectable from the start in all strains, the establishment of a positionally stable aerotactic band was delayed in absence of *cetBA_2_*, but accelerated in the complemented ∆*cetBA_2_* strain and the WT with an additional copy of the MCP genes (Figure S12, Supporting Information). This, in conjunction with changes in band localization (Figure 4B‐i), suggests that CetB_2_ and CetA_2_ mediate a repellent response to oxygen and influence the spatio‐temporal dynamics of aerotactic band formation. This hypothesis gains further support by the aerotactic band shifting farther from the meniscus in the complemented ∆*cetBA_2_* strain or the WT with an additional copy of *cetBA_2_*, compared to the WT, likely due to elevated CetBA_2_ levels (Figure 4B‐i). Additionally, the CetBA_2_‐overproducing WT shows altered cell distribution within the aerotactic band, with more cells in hypoxic regions (Figure 4A).

Distortion of the aerotactic band in the ∆*cetBA_2_* strain (Figure 4) persisted when microcapillary experiments were repeated under a magnetic field (Figure S14, Supporting Information), indicating this phenotype is independent of magnetic fields (see Supplemental Results & Discussion, Supporting Information). Additionally, consistent with Figure 2, single‐cell tracking^[^ ^39^ ^]^ (Figure S15 and Table S3, Supporting Information) and magnetic response measurements employing an automated magnetic optical density meter^[^ ^47^ ^]^ (Figure S16, Supporting Information) substantiated the lack of impairment in magnetic sensing in the ∆*cetBA_2_* strain, which is not contingent on active perception of magnetic fields by CetBA_2_.

Collectively, all observations suggest that aerotaxis, but not magnetic orientation, is compromised in the absence of *cetBA_2_*, leading to an orientation toward oxygen levels above the WT optimum.

### 2.4 CetA_2_ and CetB_2_ Interact

**Figure 5:**
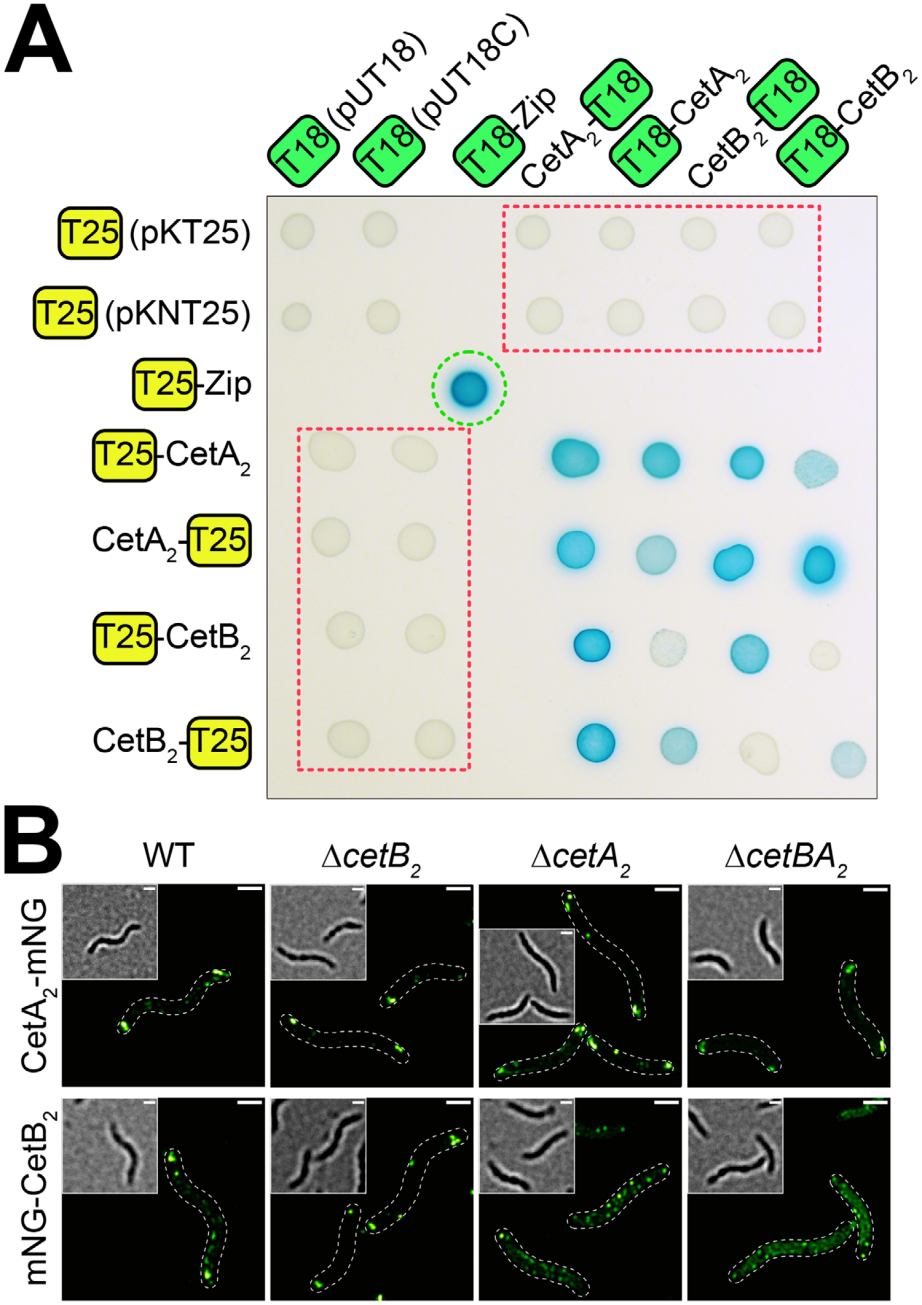
CetA_2_ and CetB_2_ interact. A) Two‐hybrid analysis indicates the interaction of both proteins and their homooligomerization by the blue color of colonies on lactose and 5‐bromo‐4‐chloro‐3‐indolyl‐β‐D‐galactopyranoside (X‐Gal)‐containing mineral salts agar. The positive control (leucine zipper) and negative controls (T18‐ or T25‐fusions with the corresponding empty vector) are marked by green and red dashed lines, respectively. A minimum of three (n ≥ 3) cotransformants were analyzed per tested interaction; shown are representative results. B) Fluorescent fusions of CetA_2_ and CetB_2_ with mNeonGreen (mNG) localize to both cell poles in the WT (3D‐SIM maximum intensity projection; cells are outlined by dashed lines, brightfield as inset). Polar‐lateral localization of the CetA_2_ receptor is independent of CetB_2_, but polar‐lateral CetB_2_ localization relies on CetA_2_, as revealed by the loss of polar mNG‐CetB_2_ signals in the ∆*cetA_2_* and ∆*cetBA_2_* strains. mNG fusion protein expression cassettes (including the constitutive P*_*mamDC45*_* promoter) were genomically inserted via Tn*5*‐based transposition. Shown are representative cells from *n* = 3 analyzed Tn*5* insertion mutants per strain. Scale bars, 1 µm.

Based on biochemical data, it was suggested that CetB mediates energy taxis in *C. jejuni* through direct interaction with CetA.^[^ ^25^ ^]^ To determine whether *M. gryphiswaldense* CetB_2_ and CetA_2_ interact specifically, an interaction analysis was conducted via heterologous expression of two‐hybrid fusion proteins in *E. coli*, utilizing the adenylate cyclase‐based bacterial two‐hybrid assay.^[^ ^48^ ^]^ Examination of adenylate cyclase activity on indicator agar plates revealed that both proteins form homooligomers and interact with each other (**Figure** **5**A). Their in vivo interaction in *M. gryphiswaldense* is supported through 3D structured illumination microscopy (3D‐SIM), demonstrating loss of polar‐lateral CetB_2_ localization in the absence of *cetA_2_* (Figure 5B). These data suggest that CetB_2_ contributes to aerotactic signal transduction through interaction with the CetA_2_ receptor in polarly localized chemotaxis arrays.

### 2.5 CetB_2_ Monitors Redox Changes Through a Flavine Adenine Dinucleotide Cofactor

**Figure 6:**
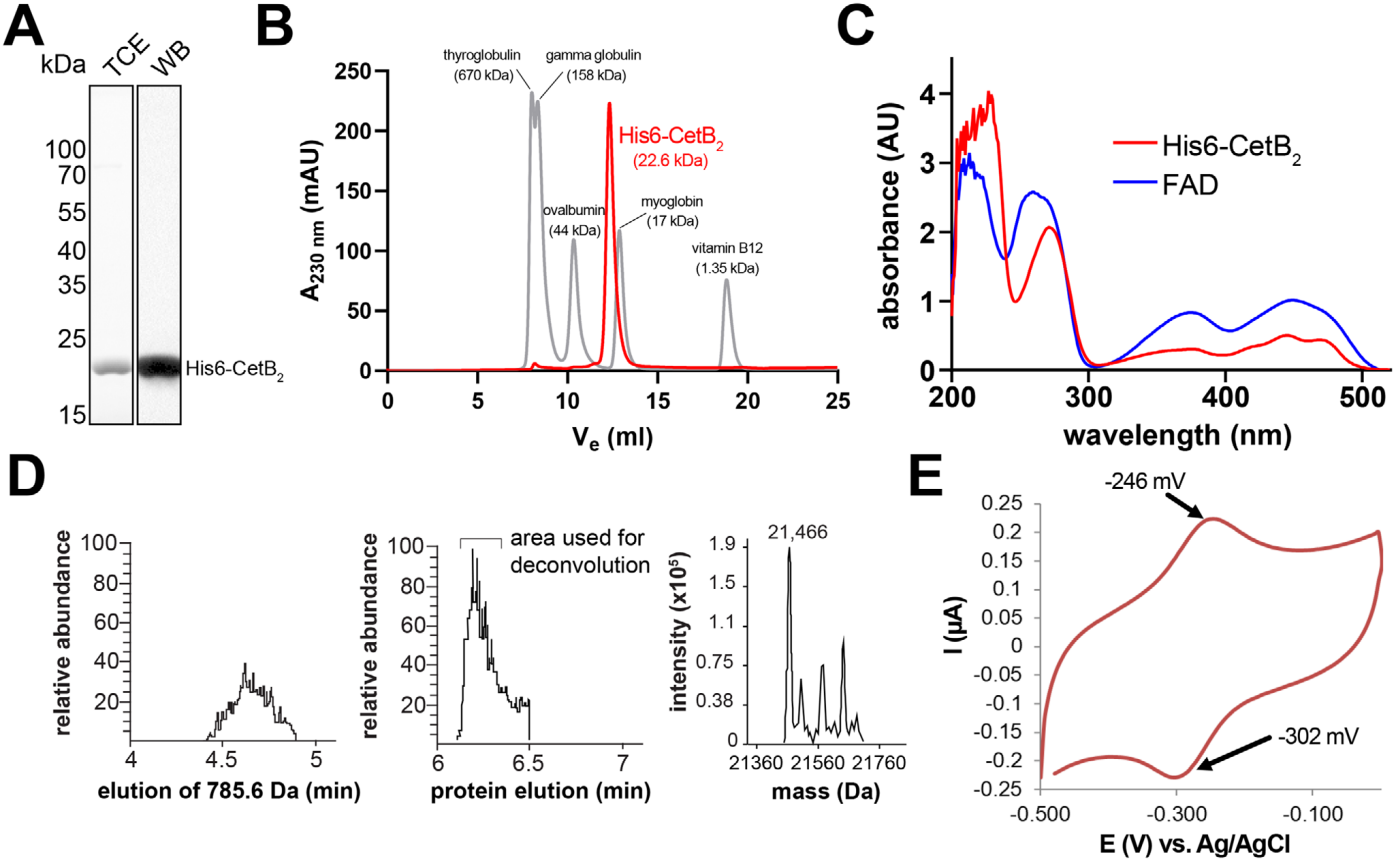
Biochemical characterization of CetB_2_. A) Sodium dodecyl sulfate polyacrylamide gel electrophoresis (SDS‐PAGE) of purified His6‐CetB_2_. Detection was performed using 2,2,2‐Trichloroethanol (TCE) in‐gel fluorescence and anti‐His‐tag Western blotting (WB). B) Analytical gel filtration chromatography of His6‐CetB_2_ on a Superdex 75 Increase 10/300 GL column yielded an apparent molecular weight of ≈22.6 kDa, consistent with FAD binding (theoretical molecular weight: 21.6 kDa + 0.786 kDa for FAD). To adjust y‐axis scaling, protein standard absorbance values (sizes indicated in brackets) were divided by ten. C) UV/vis absorbance spectroscopy of His6‐CetB_2_ (0.8 mg ml^−1^) in comparison to FAD (0.1 mg ml^−1^) both dissolved in 50 mm Na‐phosphate buffer (pH 8), 300 mM NaCl, 5% (v/v) glycerol. D) Purified His6‐CetB_2_ was subjected to LC‐MS analysis. The chromatogram depicts the elution of FAD (left plot) and His6‐CetB_2_ (middle plot). The x‐axis shows retention time; the y‐axis, relative signal intensity. The detection of FAD in the LC‐MS analysis, eluting ahead of the protein, indicates that FAD was non‐covalently bound to the protein and released during chromatographic separation. The right plot depicts the deconvoluted masses corresponding to the CetB_2_ elution peak, suggesting potential protein modifications (see Figure S18, Supporting Information). E) Electrochemical analysis of electrode‐immobilized CetB_2_ by cyclic voltammetry.

The PAS‐domain protein CetB in *C. jejuni* was proposed to sense the cellular redox state via FAD, acting as a dimer in its functional state.^[^ ^25^ ^]^ However, PAS domains bind various small‐molecule ligands.^[^ ^30^, ^31^, ^49^ ^]^ To determine the biochemical properties of *M. gryphiswaldense* CetB_2_, we purified polyhistidine‐tagged CetB_2_ (His6‐CetB_2_) via affinity chromatography (**Figure** **6**A). Purified CetB_2_ displayed an intense yellow color (Figure S17, Supporting Information), indicative of FAD binding.^[^ ^50^ ^]^ Moreover, size exclusion chromatography suggests that the discrepancy between the predicted and experimentally determined molecular weight of CetB_2_ may be due to a bound cofactor similar in size to FAD (Figure 6B). Contrary to the observed homooligomerization of CetB_2_ in vivo (Figure 5A), size exclusion chromatography indicates that CetB_2_ is monomeric under the tested in vitro conditions (Figure 6B). Ultraviolet‐visible (UV/vis) spectroscopy (Figure 6C) and Liquid Chromatography‐Mass Spectrometry (LC‐MS) (Figure 6D; Figure S18, Supporting Information), support that CetB_2_ contains FAD, with characteristic peaks near 375 nm and a trimodal peak ≈450 nm in the UV/vis spectrum.^[^ ^21^, ^50^, ^51^ ^]^ Bioinformatics confirmed conserved amino acids in CetB_2_ and other CetB paralogs (Figure S19, Supporting Information) linked to FAD binding in PAS domains.^[^ ^21^, ^23^, ^49^, ^52^, ^53^, ^54^ ^]^

Cyclic voltammetry was used to identify the FAD redox peaks in CetB_2_. Therefore, purified CetB_2_ was applied to a glassy carbon electrode and enclosed with a dialysis membrane (Figure S20, Supporting Information). Potentials from ‐0.5 to 0 V were applied against an Ag/AgCl reference electrode under an Argon atmosphere. Scans at 250 mV sec^−1^ showed an oxidation peak at ‐246 mV and a reduction peak at ‐302 mV (Figure 6E), with a midpoint potential of ‐274 mV and a narrow peak‐to‐peak separation of 56 mV. This suggests electrochemical reversibility and direct electron transfer, indicating the proximity of FAD to the electrode due to the protein structure. Fast electron transfer rates were confirmed by the reversible peaks at high scan rates. Similar peaks appeared in an aerobic environment, with a reductive current onset at ≈‐350 mV, indicating protein‐mediated oxygen reduction.

**Figure 7:**
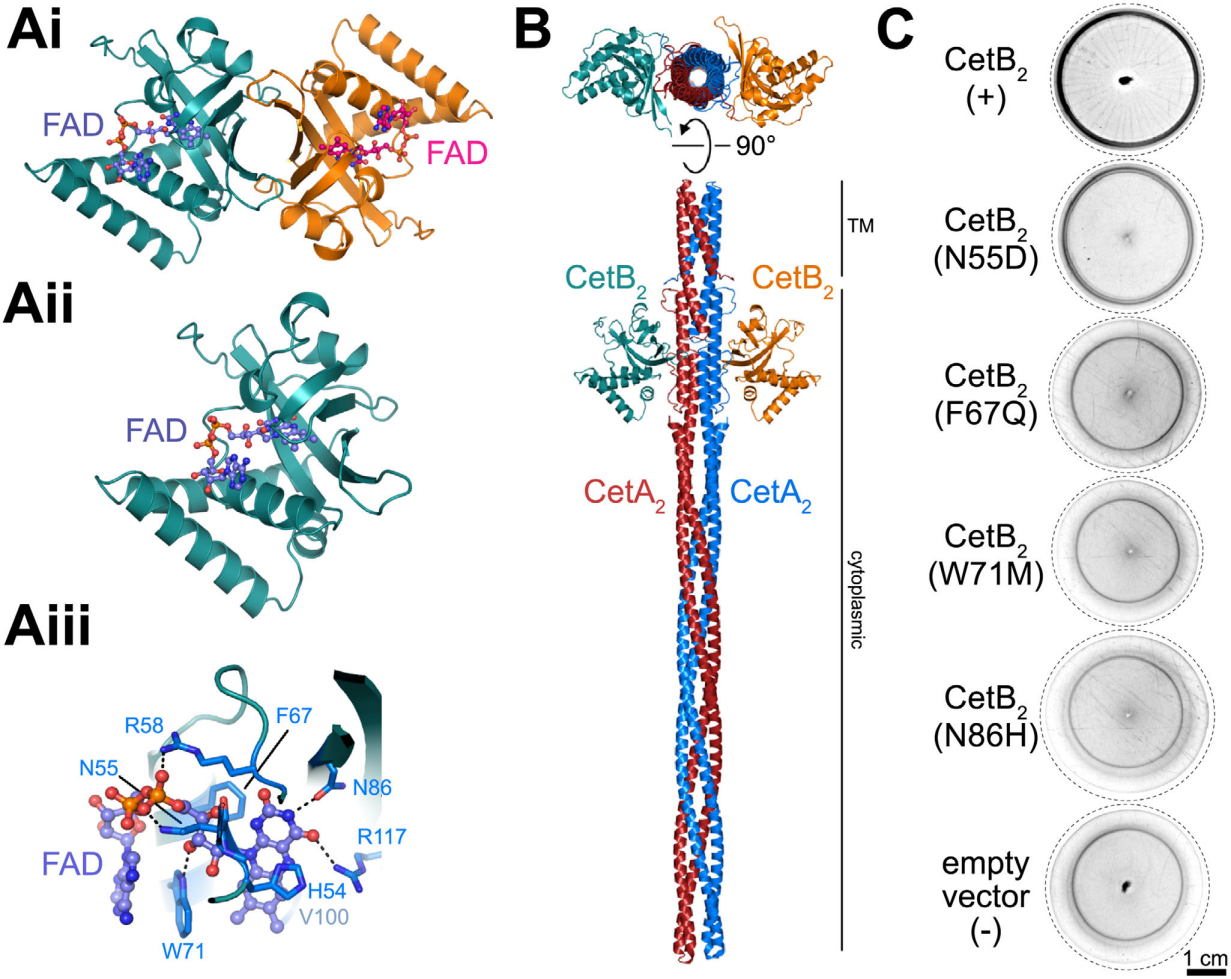
Structural models. A) AF2 predictions of (Ai) homodimeric and (Aii) monomeric CetB_2_ structures. CetB_2_ is shown as deep teal and orange cartoons. The docked FAD is shown as a ball‐and‐stick model. (Aiii) Close‐up of the FAD binding pocket. CetB_2_ is shown as a cartoon, the docked FAD is shown as a ball‐and‐stick model, and the binding residues are as sticks. Dotted lines represent hydrogen bonds. Red, blue, and orange represent oxygen, nitrogen, and phosphate, respectively. Other colors represent carbon atoms. B) AF2 prediction of a CetA_2_‐CetB_2_ complex in cartoon representation (CetA_2_: blue and red; CetB_2_: deep teal and orange; TM: transmembrane helices). C) Swim halo phenotypes of CetB_2_ variant strains with single amino acid substitutions in the FAD binding pocket. The N55D substitution introduces a negative charge that may repel the FAD phosphate group. F67Q replaces a large hydrophobic residue with a hydrophilic one, potentially disrupting FAD ring binding. W71M maintains similar size and hydrophobicity but may prevent hydrogen bonding with the FAD sugar. N86H may interfere sterically with the FAD ring due to increased side chain size. CetB_2_ variants were expressed in the ∆*cetB_2_* strain under control of the *cetBA_2_* promoter upon Tn*7*‐based insertion. Transcomplementation is indicated by a reduced spacing between inner and outer swim rings and the appearance of spoke‐wheel‐like stripes; other stripe patterns are background. The positive control (+) is the ∆*cetB_2_* strain complemented with WT *cetB_2_*. The negative control (‐) is the ∆*cetB_2_* strain with an empty Tn*7* cassette. For each construct, *n* = 3 Tn*7* insertion mutants were analyzed; representative micrographs are shown. AF2 predictions of the structural effects of mutations are shown in Figure S21B (Supporting Information).

For a deeper understanding of CetB_2_, we employed AlphaFold2 (AF2) to predict dimeric (**Figure** **7**A‐i) and monomeric (Figure 7A‐ii, Figure S21A, Supporting Information) structures. The AF2 Predicted Aligned Error (PAE) analysis indicates a strong dimeric correlation, with a dimeric interface spanning 776 Å^2^ (PISA server). However, a predicted CetB_2_‐CetA_2_ structure suggests that two CetB_2_ molecules bind to a CetA_2_ dimer as individual monomers (Figure 7B), utilizing the same interaction interface that could be involved in CetB_2_ homodimerization (Figure 7A‐i). Similar structures were obtained for other bipartite MCP complexes (Figures S22 and S23, Supporting Information), providing further evidence for a 1:1 CetA:CetB stoichiometry.

By overlapping CetB_2_ with its closest homolog in the Protein Data Bank (8DIK, the PAS domain structure of the *E. coli* Aer receptor,^[^ ^54^ ^]^ with an E‐value of 1.60e^−14^ as determined by the Foldseek server; Figure S21A, Supporting Information), we were able to directly locate the FAD cofactor within CetB_2_. The FAD is exposed to the surface (consistent with voltammetry) and is held by hydrophobic and charged residues (His54, Asn55, Arg58, Phe67, Trp71, Asn86, Val100, and Arg117; Figure 7A‐iii), similar to other FAD‐binding PAS domains.^[^ ^49^, ^54^ ^]^ To assess the importance of FAD binding, selected predicted FAD‐binding residues were mutated (Figure 7C, Figure S21B, Supporting Information). Substitutions at Phe67, Trp71, and Asn86 rendered CetB_2_ non‐functional, indicating that these residues are critical for FAD binding. In contrast, the replacement of Asn55 with Asp resulted in an intermediate phenotype, characterized by a WT‐like swim halo lacking radial stripe formation (Figure 7C), likely due to partial disruption of FAD binding caused by charge repulsion.

In summary, our data, including structural models, strongly support that CetB_2_ monitors redox changes through a bound FAD cofactor and conveys them to the CetA_2_ receptor through direct interaction.

## 3 Discussion

Many bacteria rely on chemotaxis to navigate their environments by sensing signals through chemoreceptors. Microorganisms with few chemoreceptors, like the well‐studied gut bacterium *E. coli* (five MCPs), are often adapted to relatively stable niches.^[^ ^3^ ^]^ In contrast, bacteria in dynamic habitats with significant physicochemical fluctuations, such as marine and freshwater ecosystems, sediments, and soil, often possess many MCPs.^[^ ^3^, ^55^, ^56^, ^57^ ^]^ However, the complexity of their systems limits understanding of individual chemoreceptors.^[^ ^3^ ^]^ Here, we addressed MCP function in *M. gryphiswaldense*, which contains one of the most complex chemosensory systems found in prokaryotes.^[^ ^8^ ^]^ Most MCP deletion mutants in our study exhibited relatively small changes in swim halo morphology (Figure 1), suggesting a finely tuned and partially redundant chemosensory repertoire. Such a repertoire may be vital for a gradient‐based lifestyle in complex environments like freshwater sediments. Precise localization to microaerophilic conditions is essential to maintain magnetic navigation in magnetospirilla, as excess oxygen suppresses magnetosome formation.^[^ ^58^ ^]^

In our study, the strongest phenotype was observed for the ∆*cetBA_2_* strain lacking a bipartite MCP (Figures 1 and 4) composed of a small cytoplasmic PAS domain sensory protein (CetB_2_) and an inner membrane‐linked transducer (CetA_2_). The MCP Amb0994 from *M. magneticum* ^[^ ^12^ ^]^ shares 42.1% amino acid identity with CetA_2_. Amb0994 is likely part of a bipartite system (Figure S23, Supporting Information), given its lack of a sensory domain and genomic proximity to *amb0995* (encoding a PAS‐domain protein).^[^ ^12^, ^13^ ^]^ However, no disruption of aerotaxis was detected in a ∆*amb0994‐0995* strain.^[^ ^13^ ^]^ Instead, a magnetoreceptive mechanism was suggested that involves direct interaction of the magnetosome chain cytoskeletal protein MamK with Amb0994, converting magnetic torque into a stimulus sensed by the MCP.^[^ ^12^, ^13^, ^14^, ^59^ ^]^ Intriguingly, the *amb0994‐0995* deletion strain failed to align in a 1 mT magnetic field – well above the geomagnetic range (25 to 65 µT)^[^ ^60^ ^]^ – despite intact magnetosome chains, but alignment was restored at >5 mT.^[^ ^13^ ^]^ Unlike the *M. magneticum* ∆*amb0994‐0995* strain, none of our MCP deletion mutants, including individual deletions of all bipartite systems, showed impaired magnetic alignment, including magnetic fields <1 mT (Figure 2, Figures S6B, S15, and S16, Supporting Information). Some strains even displayed an apparent improvement in magnetic alignment (Figure 2B; Figures S6B, S15B and Supplemental Results & Discussion, Supporting Information), arguing against a similar magnetoreceptive model for *M. gryphiswaldense*. Currently, we cannot exclude the existence of MCP‐cytoskeletal interactions in *M. gryphiswaldense*, a magnetoreceptive mechanism that relies on MCP redundancy (requiring multiple combined deletions), or magnetorecepetion by MCPs beyond those investigated in our study. However, future studies must also assess the proposed magnetoreceptive mechanism in *M. magneticum* ^[^ ^13^ ^]^ under physiologically relevant magnetic fields and during navigation in oxygen gradients. The prior discovery of an impaired, yet not entirely abolished, ability to align with magnetic fields just marginally stronger than the geomagnetic field in a ∆*mamK* strain^[^ ^39^ ^]^ further questions a MamK‐dependent magnetoreceptive mechanism in *M. gryphiswaldense*.

In contrast to a magnetoreceptive role, our results demonstrate that the bipartite CetBA_2_ MCP functions in aerotaxis. This function likely forms part of a general energy taxis response, involving different terminal electron acceptors (Figure S10, Supporting Information) and metabolizable substrates. Our findings show that the CetBA_2_ system influences the response to oxygen, leading to an orientation toward elevated oxygen levels in its absence (Figure 4). In contrast, increased CetBA_2_ activity promotes an aerophobic response. Thus, intracellular CetB_2_ and CetA_2_ levels need to be precisely balanced to adjust microaerophilic behavior. Consistent with our observations (Figure 4), simulations show that raising the upper threshold of favorable oxygen concentrations results in aerotactic band localization closer to the meniscus and increased band width.^[^ ^61^ ^]^ Comparable effects of chemoreceptor deletion or overexpression have been observed in non‐magnetotactic microorganisms. In *Halobacterium salinarum*, a HemAT‐type MCP promoted an aerophobic response.^[^ ^17^ ^]^ In contrast, in *A. brasilense*, deletion of *aer* caused cells to accumulate farther from the meniscus in a microcapillary.^[^ ^22^ ^]^ Similarly, Ma et al. reported that Aer overproduction caused *E. coli* to move toward the meniscus,^[^ ^62^ ^]^ and the overexpression of HemAT in *Bacillus subtilis* induced an aerophilic response as well.^[^ ^17^ ^]^

As suggested for other PAS‐domain MCPs,^[^ ^15^ ^]^ CetB_2_ (located beneath the inner membrane; Figure 7B) likely senses changes in the cellular energy state through its FAD cofactor (Figures 6 and 7A), possibly through interaction with electron transport chain components. Subsequently, the signal is transmitted to CetA_2_ through direct interaction (Figures 5 and 7B). Although PAS‐domain proteins, such as CetB in *C. jejuni* ^[^ ^25^ ^]^ or the nitrogen fixation regulatory protein NifL from *Azotobacter vinelandii*,^[^ ^53^, ^63^ ^]^ were suggested to operate as dimers, our results do not offer unequivocal evidence that CetB_2_ functions as a dimer. One possibility is that the interaction between CetB_2_ monomers in vitro (Figure 6B) is too weak for stable dimerization, while within the native context, CetB_2_ dimerization may be supported by CetA_2_ (or by chemoreceptors specific to *E. coli* during two‐hybrid analysis; Figure 5A) or the redox status of CetB_2_‐bound FAD. Alternatively, CetB_2_ may interact with CetA_2_ as a monomer, as supported by structural modeling (Figure 7B). Two‐hybrid analysis indicates that CetB_2_‐CetA_2_ interaction is favored over CetB_2_ homooligomerization, as suggested by blue colony color intensity (Figure 5A). A receptor complex with two CetB_2_ molecules individually bound to a CetA_2_ dimer aligns with structural predictions of other bipartite MCP complexes (Figures S22 and S23, Supporting Information) and high molecular weight species isolated from *C. jejuni*.^[^ ^25^ ^]^

As a final point, in *M. gryphiswaldense*, the polar‐lateral localization of CetA_2_ and CetB_2_ (Figure 5B) implies a connection to either the F5 or F7 chemotaxis pathway (encoded by *cheOp1* and *cheOp4*, respectively), both associated with distinct polar‐lateral MCP arrays.^[^ ^27^, ^64^ ^]^ Although the ∆*cetBA_2_* phenotype partially resembles that of the ∆*cheOp4* strain (Figure 1; Figure S4, Supporting Information and ref. [27]), the predicted 38‐heptad class of CetA_2_ (Dataset S1, Supporting Information) suggests that CetA_2_ (as the majority of MCPs in *M. gryphiswaldense*) signals independent of *cheOp4* in a *cheOp1*‐dependent manner. In future studies, we will explore chemoreceptor pathway linkage in *M. gryphiswaldense* to confirm molecular connections underlying magneto‐aerotaxis. The presence of multiple bipartite MCPs also makes *M. gryphiswaldense* an excellent model for studying chemotactic signal integration, interaction specificity, and redundancy in bipartite MCP systems. Additionally, these systems offer potential for bioengineering approaches, such as chimeric CetB proteins, to modulate the aerotactic response. Detailed analysis of other MCP deletion strains generated in our study, using microcapillary assays or microfluidic systems^[^ ^65^, ^66^, ^67^ ^]^ to mimic redox gradients and structure of native environments, along with further and combined MCP gene deletions, may provide further insights into how fine‐tuning of aerotaxis and sensing of other chemical cues in *M. gryphiswaldense* are controlled.

## 4 Conclusion

MTB navigate toward preferred micro‐ or anoxic environments by harnessing both passive magnetic alignment and active flagellar‐mediated motility, with the latter controlled by a chemosensory signaling system that senses environmental cues. This study uncovers molecular mechanisms of oxygen sensing in the model MTB *M. gryphiswaldense*, revealing a finely tuned system for aerotaxis. A chemoreceptor complex formed by two interacting proteins is identified as a key component of temporal oxygen gradient detection, as evidenced by misorientation of cells toward higher oxygen concentrations upon receptor deletion. Contrary to prior suggestions, this FAD‐dependent chemoreceptor complex functions independently of magnetic fields. These results highlight the complexity and versatility of bacteria in implementing sensory mechanisms to navigate their environments. Beyond advancing knowledge of bacterial motility, these findings hold potential for innovative applications in synthetic biology, such as engineering MTB with tailored oxygen responses for microrobotic applications.

## 5 Experimental Section

### Bacterial Strains and Culture Conditions

Strains are listed in Table S4 (Supporting Information). *M. gryphiswaldense* was grown in modified flask standard medium (FSM)^[^ ^58^ ^]^ at 28 °C, either in 6‐well plates without agitation (2% headspace oxygen; Scholzen Microbiology Systems AG microoxic incubator) or in polypropylene tubes with moderate shaking (120 rpm). *E. coli* was grown in lysogeny broth (LB) medium at 37 °C and shaking at 180 rpm. For cultivation of *E. coli* WM3064 (W. Metcalf, unpublished) 0.1 mm DL‐α,Ɛ‐diaminopimelic acid (DAP) was added. Media were solidified by the addition of 1.5% (wt/vol) agar. Selection was achieved by addition of kanamycin at a concentration of 5 µg ml^−1^ (*M. gryphiswaldense*) or 25 µg ml^−1^ (*E. coli*). For growth assays, cultures were adjusted to equal optical density and monitored using a microplate reader (infinite 200Pro, Tecan, Switzerland), as previously described.^[^ ^68^ ^]^ Magnetic response (*C*_mag_) was determined as previously reported^[^ ^43^, ^47^ ^]^ and detailed in the Supplemental Materials & Methods (Supporting Information). Selection and analysis of magnetotactic swimming polarity were also carried out following established protocols.^[^ ^8^ ^]^ This involved exposure of non‐agitated cultures to either a uniform 0.6 mT magnetic field (simulating Northern or Southern Hemisphere geomagnetic field polarity using coils) or a Zero Gauss Chamber as control.

### Molecular and Genetic Techniques

Oligonucleotides (Table S5, Supporting Information) were purchased from Sigma‐Aldrich (Steinheim, Germany). Genetic material was amplified using Phusion (Thermo Scientific) and Q5 (New England Biolabs) proofreading DNA polymerases. Plasmids were constructed by standard molecular techniques (see Supplemental Materials & Methods and Table S4, Supporting Information), employing FastDigest™ restriction enzymes and T4 DNA Ligase (Thermo Scientific). All constructs were sequenced by Macrogen Europe (Amsterdam, Netherlands). Vectors targeting *M. gryphiswaldense* were transferred from *E. coli* WM3064 to *M. gryphiswaldense* via conjugation. Markerless site‐specific chromosomal deletions were conducted using the homologous recombination‐based GalK‐counterselection system^[^ ^69^ ^]^ or the Cre‐*lox*‐based pCM184 vector.^[^ ^70^ ^]^ Transcomplementation experiments were carried out using a Tn*7*‐based site‐specific insertion vector as previously explained.^[^ ^68^ ^]^ Fluorescent protein fusions (using a codon‐optimized mNG‐encoding gene for *M. gryphiswaldense*) and two‐hybrid vectors were constructed as previously described,^[^ ^27^, ^68^ ^]^ using a Tn*5*‐based insertion vector^[^ ^64^, ^71^ ^]^ and the bacterial adenylate cyclase‐based two‐hybrid system,^[^ ^72^ ^]^ respectively.

### Motility and Aerotaxis Assays

Soft agar motility assays were conducted as outlined previously.^[^ ^39^ ^]^ Microcapillary assays and single‐cell tracking were also performed as previously reported, utilizing a microscope equipped with triaxial pairs of magnetic coils.^[^ ^27^, ^39^ ^]^ Methodological details are provided in the Supplemental Materials & Methods and Figures S5 and S24 (Supporting Information).

### Electron and Super‐Resolution Microscopy

TEM and 3D‐SIM were performed as previously described^[^ ^27^, ^68^ ^]^ (see Supplemental Materials & Methods, Supporting Information for details).

### Protein Biochemistry

His6‐tagged CetB_2_ was produced using *E. coli* Rosetta^TM^(DE3) pLysSRARE and the pET28a T7 RNA polymerase expression system (Novagen), followed by purification using nickel agarose affinity chromatography. Size exclusion chromatography followed previously established procedures.^[^ ^73^, ^74^ ^]^ Additional information on chromatography methods can be found in the Supplemental Materials & Methods (Supporting Information). UV/vis spectroscopy was performed using an Evolution 201 UV–vis spectrophotometer (Thermo Scientific Instruments, Madison, Wisconsin, USA). LC‐MS was performed on an LCQ Fleet mass spectrometer (Thermo Scientific) with an electrospray ionization (ESI) source. Spectra were collected in the positive ion mode and analyzed by Xcalibur software (Thermo Scientific).

### Voltammetric Measurements

Cyclic voltammetric measurements were performed using a PalmSens3 potentiostat (Palm Instruments, Houten, The Netherlands). Electrochemical measurements were conducted using a standard 3‐electrode system. This system included a disc glassy carbon electrode, an Ag/AgCl 3 M KCl reference electrode, and a graphite rod as the counter electrode. To create a stable electrochemical environment, a 150 mm phosphate‐citrate buffer at pH 5.0 was prepared. For the CetB_2_ protein sample, the initial concentration was 8.29 mg ml^−1^. To facilitate electrochemical studies, the sample was diluted tenfold with Tris 50 mm buffer at pH 7.0. The electrode preparation involved applying 10 µl of the 1:10 diluted protein sample onto the surface of a glassy‐carbon electrode. After application, the sample was allowed to dry for ≈15 min. This drying step was crucial for ensuring the uniformity and stability of the sample on the electrode surface. To prevent protein from leaching out, a 12–14 kDa dialysis membrane was placed over the electrode surface. To secure the membrane, an O‐ring and parafilm were utilized, ensuring the integrity of the setup throughout the experimental procedure (Figure S20, Supporting Information).

### Image and Sequence Analysis

Images were analyzed and processed using ImageJ Fiji.^[^ ^75^ ^]^ To enhance image clarity in figures, swim halo, microcapillary, and TEM micrographs were post‐processed using the unsharp mask filter in Affinity Photo 2.6. The filter was applied uniformly across the entire image to prevent selective enhancement of individual structures. Graphs were generated using Past 4.13^[^ ^76^ ^]^ and Prism 7.04 (GraphPad). DNA and protein sequence analysis was performed in Geneious Prime 2023.0.4 (Biomatters). Protein domains were predicted using the InterPro^[^ ^77^ ^]^ plugin within Geneious (Michael Thon and Biomatters), along with the MiST^[^ ^29^ ^]^ and KEGG^[^ ^78^ ^]^ databases.

### Statistical Analysis

Unless stated otherwise, raw data were plotted. SD and individual data points, along with violin box plots, were used to illustrate data variability, the standard error of the mean (SEM) to indicate mean precision, and CI to represent the range within which the true mean is likely to fall with the specified probability. Sample sizes (n), the statistical tests used, and the definition of *P*‐values are provided in the figure legends. Prior to the analysis of significance, data sets were tested for normality using the D'Agostino and Pearson, Shapiro‐Wilk, and Kolmogorov‐Smirnov tests. Statistical analysis was performed in Prism 7.04 (GraphPad).

## Conflict of Interest

The authors declare no conflict of interest.

## Author Contributions

D.P. conceived and designed the research. J.H., C.W., L.S., R.Z., I.A., Y.C., F.P., M.C., and D.P. performed experiments. J.H., C.W., L.S., R.Z., I.A., Y.C., F.P., C.R., M.C., L.A., M.M., D.S., D.F., and D.P. analyzed data. D.P. wrote and revised the manuscript, with input and review from all authors.

## Supporting information



Supporting Information

Supplemental Movie 1

Supplemental Movie 2

Supplemental Dataset

## Data Availability

The data that support the findings of this study are available from the corresponding author upon reasonable request.
