# Supplementary material for: A Two‐Protein Chemoreceptor Complex Regulates Oxygen Thresholds in Bacterial Magneto‐Aerotaxis
Source: Adv Sci (Weinh). 2025 Jun 25;12(34):e17315. doi: 10.1002/advs.202417315 (PMC12442639; doi:10.1002/advs.202417315)
Supplement: Supplementary file 1 — Supporting Information [file ADVS-12-e17315-s004.pdf]

ADVANCED SCIENCE
Open Access

Supporting Information

for *Adv. Sci.*, DOI 10.1002/advs.202417315

A Two-Protein Chemoreceptor Complex Regulates Oxygen Thresholds in Bacterial Magneto-Aerotaxis

*Julian Herz, Carina Weigel, Leonie Scheder, Raz Zarivach, Itay Algov, Yonatan Chemla, Felix Popp, Cornelius Riese, Mohammad A. Charsooghi, Lital Alfonta, Michael M. Meijler, Dirk Schüler, Damien Faivre and Daniel Pfeiffer**

Supporting Information for

A Two-Protein Chemoreceptor Complex Regulates Oxygen Thresholds in Bacterial Magneto-Aerotaxis

Julian Herz, Carina Weigel, Leonie Scheder, Raz Zarivach, Itay Algov, Yonatan Chemla, Felix Popp, Cornelius Riese, Mohammad A. Charsooghi, Lital Alfonta, Michael M. Meijler, Dirk Schüler, Damien Faivre, and Daniel Pfeiffer

Corresponding author: Daniel Pfeiffer
Email: daniel.pfeiffer@uni-bayreuth.de

This PDF file includes:

Supporting Text
Figures S1 to S24
Tables S1 to S5
Legends for Movies S1 to S2
Legend for Dataset S1
SI References

Other supporting materials for this manuscript include the following:

Movies S1 to S2
Dataset S1

Supplemental Materials & Methods

Molecular and Genetic Techniques

Markerless Site-specific Chromosomal Deletions

For the construction of GalK-based deletion vectors,[1] homologous arms flanking the target gene were amplified (primers are specified in **Tables S4** and **S5**), fused by overlap-extension PCR, and cloned into pORFM[1] cut with EcoRV. Flanking primers used for blunt-end cloning were phosphorylated with T4 polynucleotide kinase (Thermo Scientific). The corresponding vector backbone was dephosphorylated using FastAP thermosensitive Alkaline Phosphatase (Thermo Scientific). After conjugative transfer into *M. gryphiswaldense* and GalK-based counterselection, colonies were screened for deletion with primers flanking the modified genetic locus, and deletion was confirmed via sequencing of the PCR product. For the construction of the *MSR1_02290* deletion plasmid, a modified version of the pORFM plasmid (pFM271e_1[2]) and XhoI and XbaI restriction sites were used for cloning. *MSR1_26020* deletion was performed using the Cre-*lox* based pCM184 vector[3] as described previously.[4] For the construction of the *MSR1_26020* deletion vector, DNA fragments located upstream and downstream of *MSR1_26020* were cloned into pCM184 employing the AatII/EcoRI and HpaI/SacI restriction sites, respectively.

Tn7-based Transposon Insertion Vectors

To transcomplement the Δ*cetBA*$_2$, Δ*cetB*$_2$, and Δ*cetA*$_2$ strains, PCR fragments containing the respective missing gene(s) along with the *cetBA*$_2$ promoter were amplified (using primers 551 & 552 with genetic material from the deletion strains as template; see **Table S4** for details) and cloned into the Tn*7*-based site-specific insertion vector pBAMII-Tn*7*. pBAMII-Tn*7* was cleaved with XhoI and SmaI before ligating with the respective XhoI-cut PCR product. To generate CetB$_2$ variants with single amino acid substitutions, two PCR fragments were amplified using appropriate primer pairs (see **Tables S4** and **S5**) and then fused via overlap extension PCR to produce a full-length *cetB*$_2$ gene containing the desired mutation, which was subsequently cloned into pBAMII-Tn*7* using the same restriction sites.
Successful conjugative transfer into *M. gryphiswaldense* and genomic insertion (at the *glmS* locus) of the constructs were confirmed by PCR using appropriate primers, and the transcomplementation efficiency was evaluated via soft agar motility assays.

Tn5-based Transposon Insertion Vectors

The construction of fluorescent protein fusions was carried out utilizing a Tn*5*-based insertion vector with the constitutive P$_{mamDC45}$ promoter.[5,6] For the construction of pBAM-P$_{mamDC45}$-*cetA*$_2$-*omNG*, *cetA*$_2$ and an *M. gryphiswaldense* codon-optimized mNeonGreen[7]-encoding gene (*omNG*) were amplified with primers 630/113 and 631/632, respectively. Subsequently, both fragments were fused via overlap extension PCR employing primers 630/632 after the addition of a coding sequence for a 4-helix

linker (LAEAAAKEAAAKEAAAKEAAAKAAA) to the *omNG* gene via PCR with primers 104/632. The resulting fragment was cut with HindIII and BamHI (primers 630 and 632 contained restriction site overhangs) and ligated into the backbone of pJH39,[6] a Tn*5*-based insertion vector harboring the constitutive $P_{mamDC45}$ promoter.
For the construction of a $CetB_2$ fluorescent fusion, a codon-optimized mGreen-Lantern[8] gene (*omGL*) and *cetB*$_2$ were amplified using primer pairs 650/652 and 642/549, respectively. Both fragments were combined via overlap extension PCR (primers 650/549) after the addition of a 4-helix linker coding sequence to the *cetB*$_2$ gene employing primers 104/549. Subsequently, the resulting fragment was cloned into pJH39 using the HindIII and BamHI restriction sites. Due to poor expression of the mGreenLantern-$CetB_2$ fusion in *M. gryphiswaldense*, *omGL* was replaced with *omNG* using mega-primer PCR[9] and primers 666/667, resulting in the plasmid pBAM-$P_{mamDC45}$-*omNG-cetB*$_2$.
After conjugative transfer into *M. gryphiswaldense*, the localization and functionality of both mNeonGreen fusions was evaluated using localization microscopy and soft agar motility assays. Due to the random Tn*5*-based insertion of the expression cassette into the chromosome, at least three different transposon insertion mutants per strain and construct combination were analyzed to account for differences in the expression level. Fluorescence microscopy revealed that both fluorescent fusions localize to polar-lateral chemoreceptor arrays in the wild-type background. However, while the mNG-$CetB_2$ fusion expressed in the Δ*cetB*$_2$ background restored a wild-type-like swim halo morphology, the $CetA_2$-mNG fusion failed to restore the wild-type swim halo phenotype in the *cetA*$_2$ deletion strain. This suggests that the mNG tag affects signal transduction without impacting $CetA_2$ localization.

Magnetic Response

For magnetic response measurements, cells were cultured in FSM[10] at 28°C in 6-well plates with a 2% oxygen headspace, using a Scholzen Microbiology Systems AG microoxic incubator. To determine the magnetic response (C_{mag}),[11] cultures were diluted to an optical density of 0.1 at 565 nm. Measurements were then taken with a magnetic field applied in parallel (E_{max}) and perpendicular (E_{min}) to the light path using a powerful permanent magnet (~100 mT[12]) and an Ultrospec 2100 *pro* spectrophotometer (GE Biochrom Amersham Biosciences). The C_{mag} was calculated using the following formula:

$$C_{mag} = \frac{E_{max}}{E_{min}} - 1$$

Recording of the magnetic response as a function of the magnetic field was conducted using an open-source automated magnetic optical density meter (MagOD).[13] Therefore, the magnetic field was applied both parallel and perpendicular to the light beam (green LED), each for a duration of 20 s. Subsequently, the detector photodiode voltage (V_{DIO}) was determined at the end of each 20-s interval within a magnetic field range of 0.1-1.6 mT. The magnetic response was then calculated as outlined in

reference[13] according to the following formula, where V_{OFFSET} represents the amplifier offset voltage (3.1 V) and V_{REF} the reference value of a sample without bacteria:

$$C_{mag} = \frac{\log \frac{(V_{OFFSET} - V_{REF})}{(V_{OFFSET} - V_{DIO\ (parallel)})}}{\log \frac{(V_{OFFSET} - V_{REF})}{(V_{OFFSET} - V_{DIO\ (perpend.)})}}$$

Soft-agar Motility Assay

Swimming assays in soft agar were performed as previously described.[12] For preculturing, cells were grown for several passages in non-agitated 6-well plates under defined microoxic conditions (2% headspace oxygen; Scholzen Microbiology Systems AG microoxic incubator). Cultures were adjusted to an optical density at 565 nm of 0.1, and five microliters of diluted cell suspension were pipetted into 0.2% (w/v) soft agar, consisting of modified FSM[10] with a lowered potassium lactate concentration of 1.5 mM (Ø 14 cm-petri dishes filled with 120 ml of soft agar). Unless indicated otherwise, soft agar plates were incubated at 28°C under atmospheric conditions (21% headspace oxygen) for three days, with exposure to the local geomagnetic field. The slight difference (~14 µT) in horizontal field intensities, as depicted in **Fig. S24**, did not distort swim halos. During selected experiments (**Fig. S10**), atmospheric oxygen was reduced to 0% or 2% (instead of 21% headspace oxygen), and the $NaNO_3$ concentration in the medium was adjusted to 2 mM or 8 mM (instead of 4 mM), or $NaNO_3$ was replaced with 4 mM NH_4Cl. To examine magnetic alignment (**Fig. 2**), we employed standard petri dishes (Ø 8.5 cm) filled with 40 ml of soft agar.[14] After a two-day incubation under a uniform horizontal magnetic field (0.6 mT), facilitated by a custom coil setup,[15] plates were documented using the Gel Doc EZ Gel Documentation System and Image Lab Software 6.1.0 (Bio-Rad). Swim halo measurements were performed in ImageJ Fiji,[16] as depicted in **Fig. S5**. To enhance the visibility of swim halos, an unsharp mask filter (radius: 20 px, factor: 2, threshold: 0%) was applied using Affinity Publisher 2.6.

Microcapillary Assay and Single-Cell Tracking

Microcapillary Assay for Studying Aerotactic Band Formation

Analysis of aerotactic band formation under defined magnetic field conditions was conducted as previously described,[17] using custom-made triaxial pairs of magnetic coils mounted on a Nikon Eclipse FN1 upright microscope. The microscope was equipped with an S Plan Fluor 20× differential interference contrast (DIC) N1 objective (numerical aperture (NA), 0.5), a dark-field condenser (NA, 0.95), and a pco.edge 4.2 sCMOS camera (PCO).[12] To conduct the experiment, microoxically grown cultures (6-well plates; 2% headspace oxygen level; Scholzen Microbiology Systems AG microoxic incubator) were adjusted to an $OD_{565\ nm}$ of 0.1, and 1 ml of diluted cell suspension was purged with N_2 for 15 min. Samples were transferred into rectangular glass capillaries (0.1 by 1 mm Vitro Tubes #5010-050) by capillary forces. Capillaries were closed on

one end using vacuum grease and incubated in a zero field (geomagnetic field canceled), or alternatively a 400 μT magnetic field, at room temperature. Subsequently, images and/or videos were captured at various time points to document the spatiotemporal dynamics of aerotactic band formation. The band distance to the meniscus and intensity profiles were determined using the line length and profile tools within ImageJ Fiji.[16] An unsharp mask filter (radius: 10 px, factor: 1, threshold: 0%) was applied post-analysis to micrographs in Affinity Publisher 2.6 to improve their clarity for the figures.

Tracking of Cells in the Vicinity of the Aerotactic Band

To analyze the motility of cells in regions adjacent to the aerotactic band, the microcapillary assay was conducted as described above, and movies of the aerotactic band were recorded after 180 min. For movie recording, the camera region of interest (ROI) was set to 1,500 by 1,500 pixels. Movies were recorded for 40 s with a frame rate of 25 frames per s (fps). For single-cell tracking, movies were cropped, resulting in analysis regions extending ~150 μm on each side of the aerotactic band. Single-cell tracking was conducted in NIS-Elements (Nikon) as previously described,[12] using the ‘Spot Detection’ algorithm and ‘Random Motion’ model with the ‘Standard Deviation Multiplication Factor’ set to 2.5, ‘Maximum Gap Size’ to 3, and ‘Maximum Object Speed’ to ‘infinity.’ Tracks with 30 or fewer frames and line speeds lower than 5 μm/s were excluded from the analysis.

Single-Cell Tracking for Analysis of Magnetic Alignment

Single-cell tracking to investigate magnetic alignment was performed under uncontrolled atmospheric conditions, following established protocols.[12] In brief, cells were grown in FSM[10] in 6-well plates at 28°C with a 2% oxygen headspace (Scholzen Microbiology Systems AG microoxic incubator), diluted to an optical density of 0.01 at 565 nm, and then observed using dark-field microscopy on a Nikon Eclipse FN1 upright microscope equipped with three pairs of magnetic coils.[12] To quantify magnetic alignment, we calculated the variance of sin θ for a population of bacteria. This calculation is based on the average heading angles (θ) from individual single-cell trajectories, which denote the orientation of the velocity vector relative to the magnetic field vector axis when projecting swimming trajectories onto the focal plane.[12,18,19] The variance of sin θ offers a quantification of the spread of cell orientations in relation to consistent magnetic field lines, and is expected to decline as the magnetic field strength increases.[12,19] In cases where cells move randomly, the variance of sin θ approaches 0.5. Conversely, if cells tend to align with the magnetic field, the value nears zero, signifying a high degree of alignment.

Transmission Electron Microscopy

For transmission electron microscopy (TEM), cells were grown at 28°C under microaerobic conditions (2% headspace oxygen). For TEM sample preparation, cells were fixed by adding formaldehyde (1.5%) and subsequent incubation at 4°C for at least 1 h. Cells were concentrated by centrifugation of 1 ml of culture (OD_{565} ~0.15) at

3,500 *g* for 3 min, followed by resuspension in ~50 µl of the residual medium. Afterward, cells were adsorbed onto carbon-coated copper mesh grids (CF200-CU, Electron Microscopy Sciences, Pennsylvania) and washed two times with millipore water. Images were recorded with an EM Zeiss 902A transmission electron microscope at 80 kV accelerating tension. For the assembly of **Fig. S7A-B**, an unsharp mask filter (radius: 10 px, factor: 2, threshold: 0%) was applied to the TEM images using Affinity Publisher 2.6 to enhance the visibility of structural details. To generate magnetosome localization heatmaps (**Fig. S7C**), TEM images were segmented using trainable Weka segmentation[20] in ImageJ Fiji[16] to extract cell outlines and magnetosomes, followed by analysis of binary images in CHAP (Chain Analysis Program[21]). Magnetosome numbers per cell (**Fig. S7D**) were determined manually using the Cell Counter tool in ImageJ.

Structured Illumination Microscopy

3D-SIM (striped illumination at 3 angles and 5 phases) imaging was conducted using a Nikon Eclipse Ti2-E N-SIM E fluorescence microscope, featuring a CFI SR Apo TIRF AC 100×H NA1.49 Oil objective lens, a hardware-based 'perfect focus system' (Nikon), LU-N3-SIM laser unit (Nikon) (488, 561, and 640 nm wavelength lasers; emission filters: 525/50, 605/70, and 700/75 nm), and an Orca Flash4.0 LT Plus sCMOS camera (Hamamatsu). Sample preparation involved 'MSR agarose pads' and high-precision coverslips (0.17 mm thickness, no. 1.5H; Marienfeld), while calibration of the objective correction collar and SIM grating focus utilized TetraSpeck fluorescent beads (T-7279 TetraSpeck microspheres), following established protocols.[22] 3D-SIM *z*-series were acquired as described previously[17,22,23] with 120 nm *z*-step spacing. Image reconstruction was carried out in NIS-Elements 5.01 (Nikon) using the 'Stack Reconstruction' algorithm, with 'Illumination Modulation Contrast' set to 'auto' and 'High-resolution Noise Suppression' set to 0.1. Images were analyzed and processed using ImageJ Fiji.[16] Cell contours in brightfield micrographs were identified with MicrobeJ.[14]

Two-Hybrid Assay

For protein interaction studies using the adenylate cyclase (CyaA)-based bacterial two-hybrid assay,[24] genes of interest were amplified from chromosomal DNA of *M. gryphiswaldense* and cloned into pUT18C, pUT18, pKT25, and pKNT25 plasmids (refer to **Tables S4** and **S5** for restriction sites and primers). The resulting plasmids were verified by DNA sequencing and cotransformed into the *cya*$^-$ *E. coli* BTH101 reporter strain. Cotransformants were plated on LB agar supplemented with 40 µg ml^{-1} 5-bromo-4-chloro-3-indolyl-β-D-galactopyranoside (X-Gal), 0.5 mM isopropyl β-D-1-thiogalactopyranoside (IPTG), ampicillin (100 µg ml^{-1}), and kanamycin sulfate (50 µg ml^{-1}). Incubation took place at 28°C, and colonies were subsequently assessed for blue color development. To visualize the results, cotransformants were cultured overnight at 28°C in LB liquid medium containing IPTG (0.5 mM), ampicillin (100 µg ml^{-1}), and kanamycin sulfate (50 µg ml^{-1}). Three microliters of cell suspension were spotted on M63 mineral salts agar supplemented with 0.2% (w/v) maltose, X-Gal (40 µg

ml^{-1}), 0.5 mM IPTG, ampicillin (50 µg ml^{-1}), and kanamycin sulfate (25 µg ml^{-1}). M63 plates were incubated at 28°C for 32 h and photographed using a Lumix DMC-FZ38 camera (Panasonic).

Protein Purification

To heterologously produce His$_6$-tagged CetB$_2$, *E. coli* Rosetta^{TM}(DE3) pLysSRARE containing pET28a-*cetB$_2$* (**Table S4**) was grown at 28°C in 4x 400 ml LB medium containing 50 µg/ml kanamycin and 34 µg/ml chloramphenicol (2 l Erlenmeyer flask shaking at 150 rpm; inoculated 1:20 with an overnight culture). Gene expression was induced with 0.1 mM IPTG at an optical density of ~0.8 at 600 nm, and cells were harvested by centrifugation after 18 h of incubation at room temperature. Cell pellets were stored at -20°C until further use. For each pellet, each corresponding to one of the four cultures, 15 ml of ice-cold 50 mM Na-phosphate buffer (pH 8), 300 mM NaCl, 5% (v/v) glycerol, and 20 mM imidazole was added. The resuspended cells were then combined, pooling two 15 ml aliquots at a time to produce two separate 30 ml preparations. Cells were subsequently disrupted by three passages through a cooled French press chamber (Aminco, Silver Spring, MD, USA) at 96 MPa, after adding 100 µl of DNase I (1 mg/ml) to reduce sample viscosity. To separate soluble proteins from cell debris, membranes, and other insoluble material, the samples were centrifuged for 2 h at 35,000 rpm using a Ti 50.2 rotor in a Sorval WX Ultra Series Centrifuge (Thermo Fisher Scientific) at 4°C. His6-tagged CetB$_2$ was purified from the soluble fraction via Ni-NTA affinity chromatography utilizing 5 ml gravity flow polypropylene columns (Pierce) and Ni-NTA agarose (Jena Bioscience). Purification was performed at room temperature, and His6-CetB$_2$ was eluted using a stepwise imidazole gradient. Imidazole and other low-molecular-weight molecules were removed by size exclusion chromatography (PD-10 desalting column). Purified His6-CetB$_2$ was concentrated via ultrafiltration, aliquoted, and stored on ice for a few days or otherwise at -80°C until use. Protein samples were analyzed by SDS-PAGE and immunoblot using a monoclonal anti-Histidine-tag-specific antibody (mouse, Bio-Rad, MCA1396, LOT 1711). Protein concentrations were determined using the colorimetric ROTI®Quant PCA kit (Carl Roth).

Analytical Gel Filtration Chromatography

Size-exclusion chromatography was carried out utilizing a Superdex 75 Increase 10/300 GL column (geometric column volume (V_c) of 23.56 ml) on an ÄKTA pure system (GE Healthcare) operated at room temperature at a flow rate of 0.5 ml/min. The column void volume (V_0) was determined with blue dextran 2000 to 7.96 ml. One hundred microliters of purified His6-CetB$_2$ (0.8 mg/ml) were applied to the column and eluted with 50 mM Na-phosphate buffer (pH 8), 300 mM NaCl, 5% (v/v) glycerol, with detection at 230 nm, 280 nm, and 450 nm. The apparent molecular weight of His6-CetB$_2$ was determined after calibration of the column using standard proteins (Bio-Rad gel filtration standard, catalog-no. 151-1901). To this end, the partition coefficient (K_{av}) for each protein was calculated using the elution volume (V_e) and the formula $K_{av} = (V_e - V_0)/(V_c - V_0)$.

Supplemental Results & Discussion

Improvement in Magnetic Alignment Following Deletion of MCP Genes

Contrary to a model proposed in *M. magneticum* (see **Introduction** and **Discussion**), which suggests that magnetic alignment is stabilized by interaction between the magnetosome chain cytoskeletal protein MamK and the bipartite MCP Amb0994-95,[25,26] none of our *M. gryphiswaldense* MCP deletion strains – including individual deletions of all bipartite systems – showed impaired magnetic alignment (**Figs. 2**, **S6B**, **S15A-B**, **S16**; **Table S3**), even under weaker magnetic field intensities than those tested in the *M. magneticum* study. In fact, in some cases, we even observed improved magnetic alignment (**Figs. 2B**, **S6B**), suggesting that a similar magnetoreceptive model does not apply to *M. gryphiswaldense*. For example, magnetic alignment of the *MSR1_02290* deletion strain (Δ*mcp-cache*) was increased in both the soft agar motility assay (**Fig. 2**) and magnetic response measurements (**Fig. S6B**). This observation contradicts previous findings,[27] suggesting that the non-magnetic phenotype of a *MSR1_02290* Tn-insertion mutant reported by Silva et al.[27] may have been caused by a spontaneous second-site mutation in magnetosome genes or by polar effects of the Tn-insertion on the expression of nearby genes (**Fig. S2**) involved in anaerobic respiration (*napFDAGHBC*[28]) and/or magnetosome formation (magnetosome island (MAI)[29]).

The causes of the enhanced magnetic alignment in certain MCP deletion strains (such as the Δaer_1 strain and hemerythrin MCP deletion mutants, in addition to Δ*mcp-cache*) in our study remain elusive. TEM analysis of cells revealed minor, non-significant differences between strains (**Fig. S7**), suggesting that changes in magnetosome number or chain configuration are unlikely to account for improved magnetic alignment. Alternatively, an improvement in magnetic alignment could potentially be related to an altered reversal behavior (associated with modified chemo- or aerotactic sensing abilities), which, in turn, is linked to the frequency of changes in flagellar polymorphisms that may affect the orientation of cells and contribute to changes in magnetic alignment.[30] Although this effect may be more pronounced in soft agar motility assays – where swim halo distortion in a magnetic field depends on both motility- and growth-driven spreading[12] – magnetic response measurements, which directly assess magnetic alignment independent of growth, can still be influenced by cell motility.[31] Additionally, the outcomes of both assays can be affected by changes in cell morphology, potentially leading to an apparent improvement in magnetic alignment. Cell shape influences spreading in soft agar[23] and may also impact light scattering properties of cells during magnetic response measurements, along with other factors such as intracellular storage granules.[11] **Fig. S7C** indicates possible subtle changes in cell length or curvature in some of the MCP deletion strains (e.g., cells lacking Aer-type receptors appear slightly shorter than wild-type cells), which, however, require further quantification.

To draw more conclusive evidence of an actual increase in magnetic alignment (as indicated by soft agar and C_{mag} assays), further investigation using techniques such as single-cell tracking[12] and the MagOD device[13] may be helpful. Both methods

enable the application of defined, homogeneous magnetic fields over a range of intensities. This overcomes limitations of the original C_{mag} assay, which relies on strong, inhomogeneous fields generated by permanent magnets.[12] Although a comprehensive characterization of all MCP deletion mutants using these techniques – that were exemplarily used to characterize the Δ*cetBA*$_2$ strain (**Figs. S15, S16**) – was beyond the scope of this study, our results clearly show that magnetic alignment is neither significantly impaired nor lost in any of our *M. gryphiswaldense* MCP mutants, unlike the severe impairment seen with *amb0994-95* deletion in *M. magneticum*.[26]

Analysis of CetBA$_2$ Function in Aerotaxis via Single-Cell Tracking

To gain deeper insights into the role of the CetBA$_2$ MCP in aerotactic band formation, we analyzed wild-type, Δ*cetBA*$_2$, Δ*cetB*$_2$, and Δ*cetA*$_2$ cells using single-cell tracking. Swimming trajectories were analyzed in the vicinity of the aerotactic band (~150 µm on either side) under zero-field conditions (**Fig. S13A**) utilizing a specialized microscope[12] (see **Supplemental Material & Methods** for details).
Swimming speeds near the aerotactic band (**Fig. S13B**, **Table S2A**) were consistent with previous estimates and observations linking speed to cellular energy levels.[32,33] The wild-type strain exhibited higher swimming speeds on the hyperoxic side of the band – where oxygen concentrations exceed optimal levels – averaging 18.6 ± 0.17 µm/s in hypoxic regions and 25.1 ± 0.37 µm/s in hyperoxic regions (**Table S2A**). In contrast, the Δ*cetBA*$_2$, Δ*cetB*$_2$, and Δ*cetA*$_2$ strains showed reduced swimming speeds, by up to 14% in hypoxic and 24% in hyperoxic regions, compared to the wild type. This reduction, especially in hyperoxic areas, may result from the more diffuse aerotactic bands formed by these mutants, which likely increase cell collisions resulting in lower apparent swimming speeds. This is supported by the observation that Δ*cetBA*$_2$ cells showed no reduction in swimming speed compared to the wild type in diluted suspensions (**Fig. S15C**, **Table S3B**).
Near the aerotactic band, mean reversal rates were slightly higher under hyperoxic conditions (**Table S2B**), but no significant differences were detected between hypoxic and hyperoxic regions or among the strains (**Fig. S13C**). Notably, reversal rates near the band (~0.9 s^{-1}) were higher than those previously reported for anoxically adapted *M. gryphiswaldense* cells during oxygen upshift experiments in a microoxic chamber (up to 0.34 s^{-1}).[15]

Analysis of Aerotactic Band Formation in a Magnetic Field

Although our primary focus was to study aerotaxis phenotypes without magnetic field influence (**Figs. 4**, **S12**), we also analyzed aerotactic band formation in the presence of a 400 µT magnetic field (**Fig. S14**), as a previous study indicated this enhances aerotactic band formation.[32] Moreover, we aimed to clarify whether the MCP CetBA$_2$ plays a role in a magnetoreceptive mechanism, as suggested for a related bipartite MCP in *M. magneticum*.[26]

While we were able to reproducibly study aerotactic band formation under zero-field conditions, indicating the robustness of aerotaxis without an applied magnetic field, the position of the band for both the wild type and the Δ*cetBA*$_2$ mutant during the initial phase of band formation was more consistent across individual measurements when a magnetic field parallel to the oxygen gradient was applied (compare error bars of curves in **Figs. S12B**, **S14Ci**). In both the wild type and the Δ*cetBA*$_2$ strain, the band also appears to approach a quasi-stable position more rapidly under a magnetic field than under zero-field conditions, although it had not fully stabilized by the end of the experiments (compare slopes of curves in **Figs. S12B**, **S14Ci**). Moreover, compared to zero field conditions (**Figs. 4B, S12B**), under the influence of a magnetic field, the aerotactic band for the Δ*cetBA*$_2$ mutant remained farther from the meniscus throughout the experiments (**Fig. S14Ci-ii**), resulting in a non-significant difference compared to the wild type. However, the distorted appearance of the aerotactic band in the Δ*cetBA*$_2$ strain observed under zero-field conditions (**Fig. 4A**) was still preserved (**Figs. S14B**), indicating this phenotype is independent of magnetic fields. Interestingly, both the wild type and Δ*cetBA*$_2$ mutant showed increased cell localization (by ~20% compared to zero field) to the aerotactic band (cell samples adjusted to similar optical densities), as evidenced by the comparison of intensity profiles in **Figs. 4A** and **S14Ciii**. Additionally, the width of the aerotactic band increased in both strains compared to zero-field conditions (**Figs. 4Bii**, **S14Civ**). This suggests that the magnetic field may indeed improve cell localization to preferred microoxic conditions.
In conclusion, the persistence of an impaired aerotaxis phenotype in the Δ*cetBA*$_2$ strain under both zero-field and magnetic field conditions, combined with largely similar altered spatiotemporal band formation dynamics in the wild type and Δ*cetBA*$_2$ strains upon magnetic field exposure, argues against a role for CetBA$_2$ in magnetoreception. Instead, the observed effects on band formation in both strains likely result solely from passive magnetic alignment.

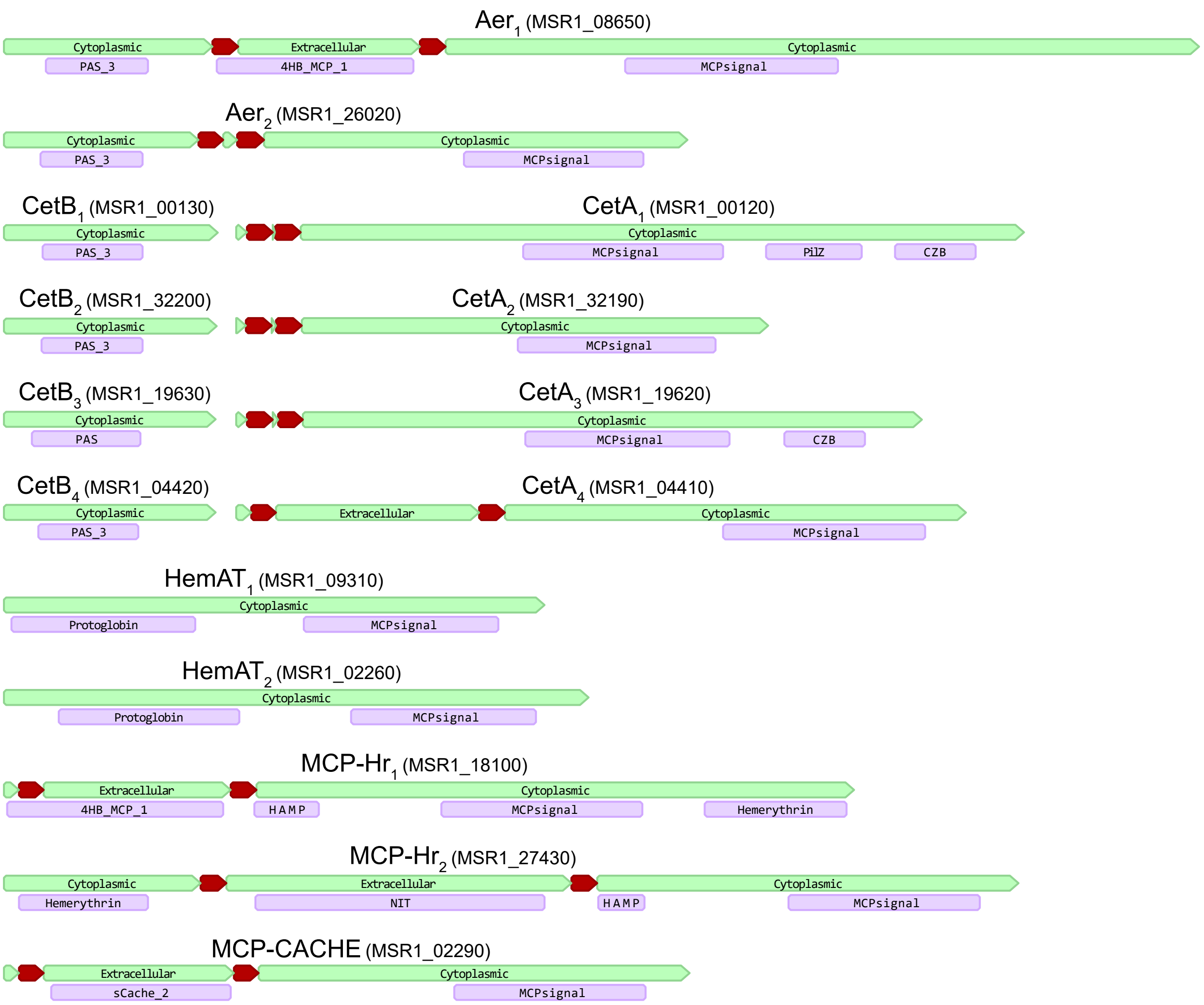

Fig. S1. MCPs investigated in this study. Protein motifs were predicted using InterProScan and Geneious Prime 2023.0.1. For simplicity, only the results produced by Pfam (colored purple), TMHMM (red arrows), and a topological domain prediction (colored green) are shown. Predicted putative sensory domains include PAS (Per-Arnt-Sim; in Aer- and bipartite CetBA-type MCPs), protoglobin (HemAT-type MCPs), hemerythrin (MCP-Hr), and sCache2 (MCP-CACHE). Some MCPs contain additional (putative) sensory domains, such as a four-helix bundle (4HB) motif in Aer$_1$, a nitrate- and nitrite-responsive (NIT) domain in MCP-Hr$_2$, a cyclic diguanylate (c-di-GMP)-binding PilZ domain in CetA$_1$, and a chemoreceptor zinc-binding (CZB) domain in CetA$_1$ and CetA$_3$. In the case of the protoglobin-type MCPs HemAT$_1$ and HemAT$_2$, no transmembrane motifs (red arrows) were detected.

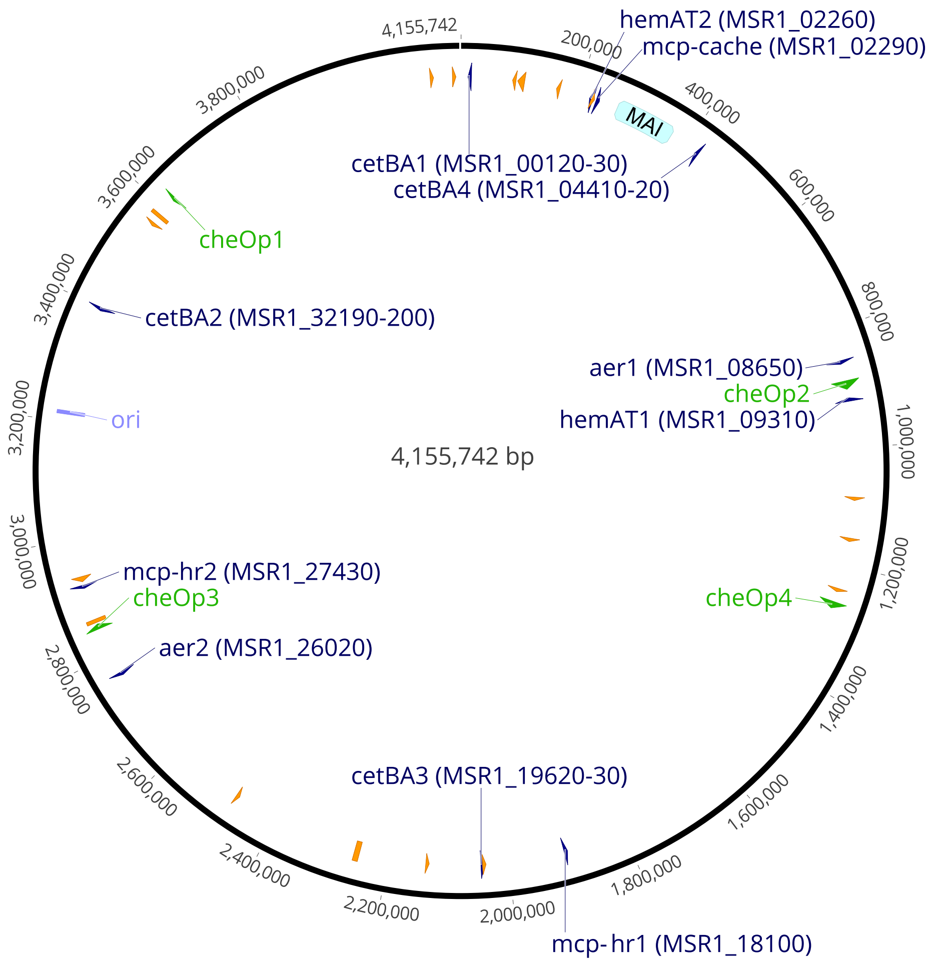

Fig. S2. Genomic localization of MCP genes and related functional elements. The figure illustrates the genomic distribution of MCP-encoding genes (shown in dark blue), which were individually deleted in the present study. Additionally, magnetosome biogenesis genes forming the magnetosome island (MAI) are highlighted in turquoise, and the major chemotaxis operons *cheOp1-4* in green, along with flagellar biosynthesis genes in orange. The chemotaxis systems encoded by *cheOp1* and *cheOp4*, are classified as F5 and F7, respectively, which are related to flagellar motility. In constrast, *cheOp2* and *cheOp3* are related to alternative cellular functions (ACF) systems with hitherto unknown functions. For simplicity, other MCP and chemotaxis genes are not highlighted. A complete list of MCPs is provided in **Dataset S1**.

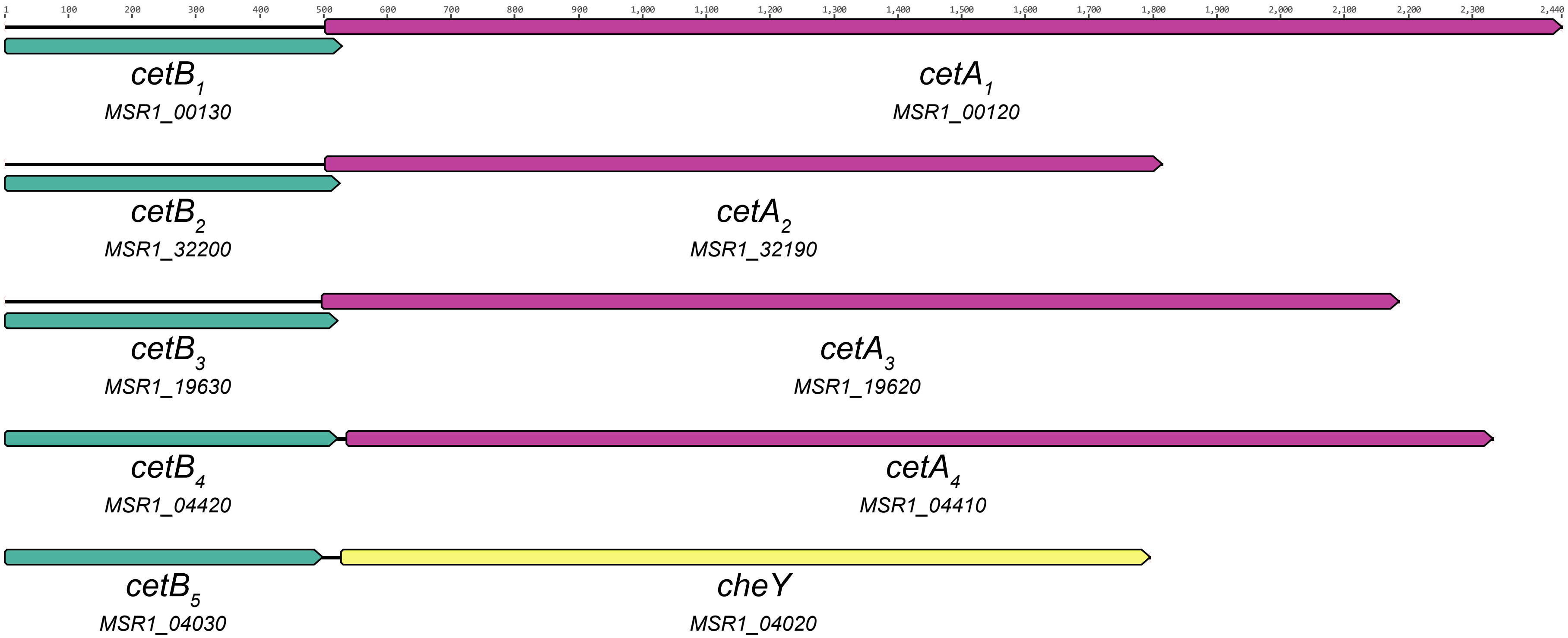

Fig. S3. Genetic organisation of *cetBA* loci. In *M. gryphiswaldense*, four operons encoding bipartite MCPs were identified. These operons consist of two overlapping genes each, with the exception of *cetB*$_4$ and *cetA*$_4$, which are not overlapping. Compared to *C. jejuni*, the order of genes is inverted in *M. gryphiswaldense*, with *cetB* positioned ahead of *cetA*. In *C. jejuni*, *cetA* is followed by *cetB*, while an additional CetB paralog named CetC is encoded in front of *cetA*. An additional fifth CetB paralog (MSR1_04030) is present in *M. gryphiswaldense*, but it lacks the CetA counterpart. This CetB paralog is encoded adjacent to a putative *cheY* gene.

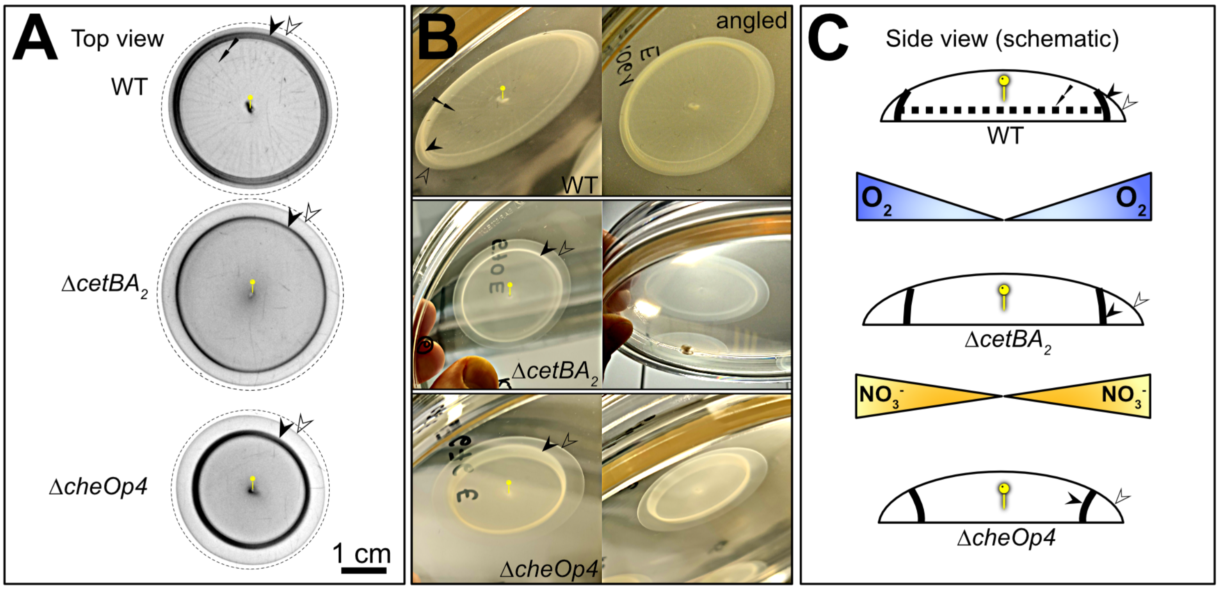

Fig. S4. Three-dimensional (3D) architecture of swim halos. (A) Micrographs and **(B)** photographs captured from various angles display representative swim halos formed after three days by the wild-type (WT), Δ*cetBA*$_2$, and Δ*cheOp4* strains. **(C)** Schematics portray the 3D morphology of distinct inner and outer ring structures, which are possibly related to the ability of *M. gryphiswaldense* to respond tactically and metabolically to the terminal electron acceptors nitrate and oxygen (see also **Fig. S10**). Depending on the respective strain, the inner ring (marked by black arrowheads) is characterized by a conical cylinder with varying base and top radii. The outer ring (white arrowheads) exhibits a dome-shaped appearance. In the wild type, radial stripes (black double arrowheads) arranged in a spoke-wheel-like pattern are visibile. Other stripe patterns are due to background. Note that the swim rings of the Δ*cetBA*$_2$ and Δ*cheOp4* strains, while sharing similarities such as an increased distance between inner and outer rings and the lack of spoke-wheel-like stripes (in comparision to the wild type), also exhibit notable differences. In the Δ*cetBA*$_2$ strain, the increased inner-outer ring distance is due to an enlarged outer ring, whereas in the Δ*cheOp4* strain, it results from a reduced inner ring size (see also **Fig. 1**). Moreover, the inner ring of the Δ*cheOp4* strain adopts a more conical 3D shape, leading to an increased 2D thickness, whereas the inner ring of the Δ*cetBA*$_2$ strain appears thinner than in the wild type due to its reduced conical shape. The inoculation spot is indicated by a yellow pin symbol. In 'C', the putative distributions of oxygen and nitrate in the medium are indicated. These distributions are influenced by the tactic responses and spread of cells, their metabolic activity including oxygen and nitrate consumption through aerobic and anaerobic respiration, respectively, and the diffusion of atmospheric oxygen into the soft agar.

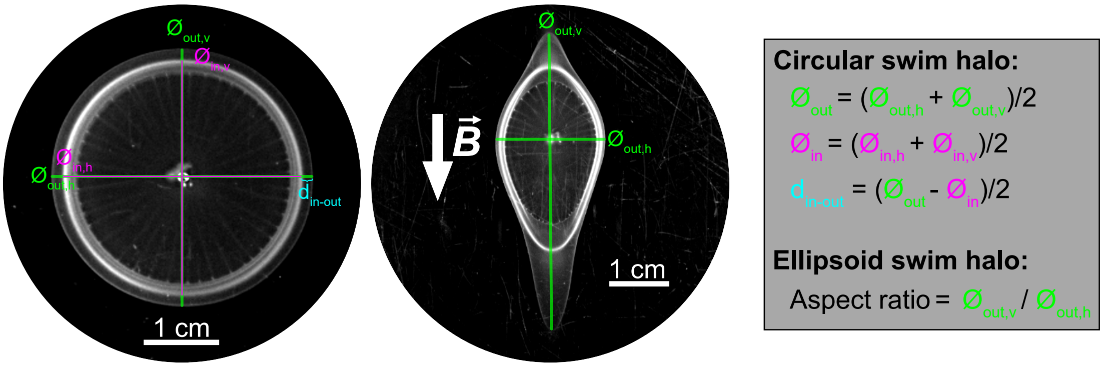

Fig. S5. Swim halo measurements were conducted to analyze circular and ellipsoid swim halos. For circular swim halos, we quantified the inner (magenta line) and outer (green line) ring diameters by calculating their respective means based on measurements in both horizontal and vertical directions. Subsequently, we determined the distance between the inner and outer rings (denoted as '$d_{in\text{-}out}$' and represented by a turquoise-colored bracket) by subtracting the inner diameter from the outer diameter and then dividing the result by two. For ellipsoid swim halos, which were obtained after incubating plates in a homogeneous 600 μT magnetic field, we calculated the aspect ratio by dividing the vertical by the horizontal outer ring diameter.

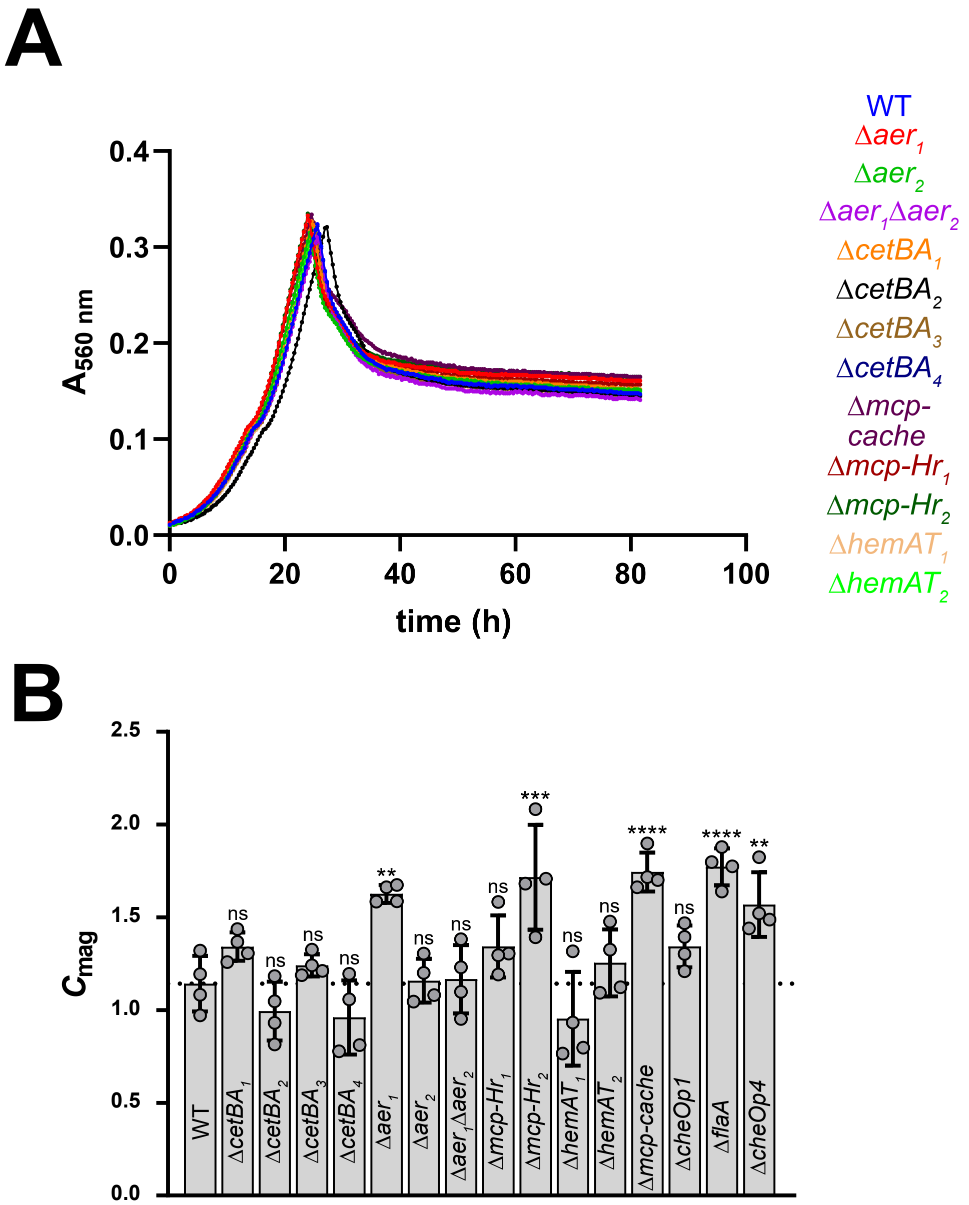

Fig. S6. Growth analysis and magnetic response measurements of the wild type and MCP deletion strains. (A) Growth was monitored at 28°C in 24-well plates, conducted under an air atmosphere with periodic shaking. Growth measurements were taken at 20-min intervals, and between measurements, the plates were shaken for 30 s every 5 min with an amplitude of 6 mm in orbital mode. The data shown is from a representative experiment out of n = 3 independent culturing experiments. **(B)** Magnetic response (C_{mag}) from n = 4 independent growth cultures. Dots represent individual experimental results, bars indicate the mean, and error bars show the SD. The dotted line represents wild-type behavior. In addition to the wild type and MCP deletion strains, non-motile (Δ*flaA*), non-aerotactic (Δ*cheOp1*), and aerotaxis-impaired (Δ*cheOp4*) strains were included during magnetic response measurements. Statistical analysis was performed using one-way analysis of variance (ANOVA) with Dunnett's multiple-comparison test to compare mutants against the wild type. **, $P < 0.01$; ***; $P < 0.001$; ****, $P < 0.0001$; not significant (ns), $P \geq 0.05$.

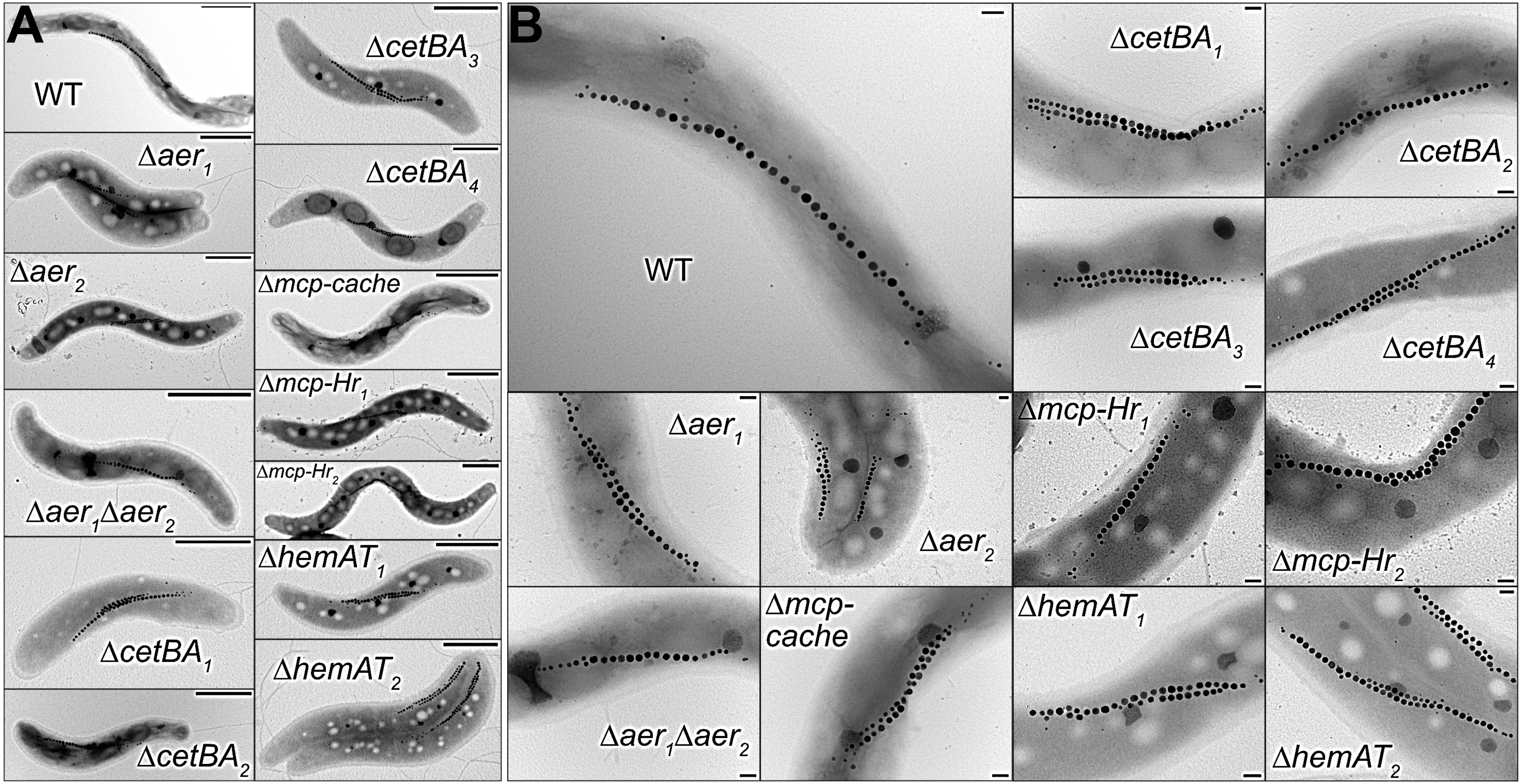

Fig. S7 (continued on next page). Representative TEM micrographs of the wild type (WT) and MCP deletion strains. (A) Cells (scale bars, 1 μm). **(B)** Magnetosome chains (scale bars, 100 nm).

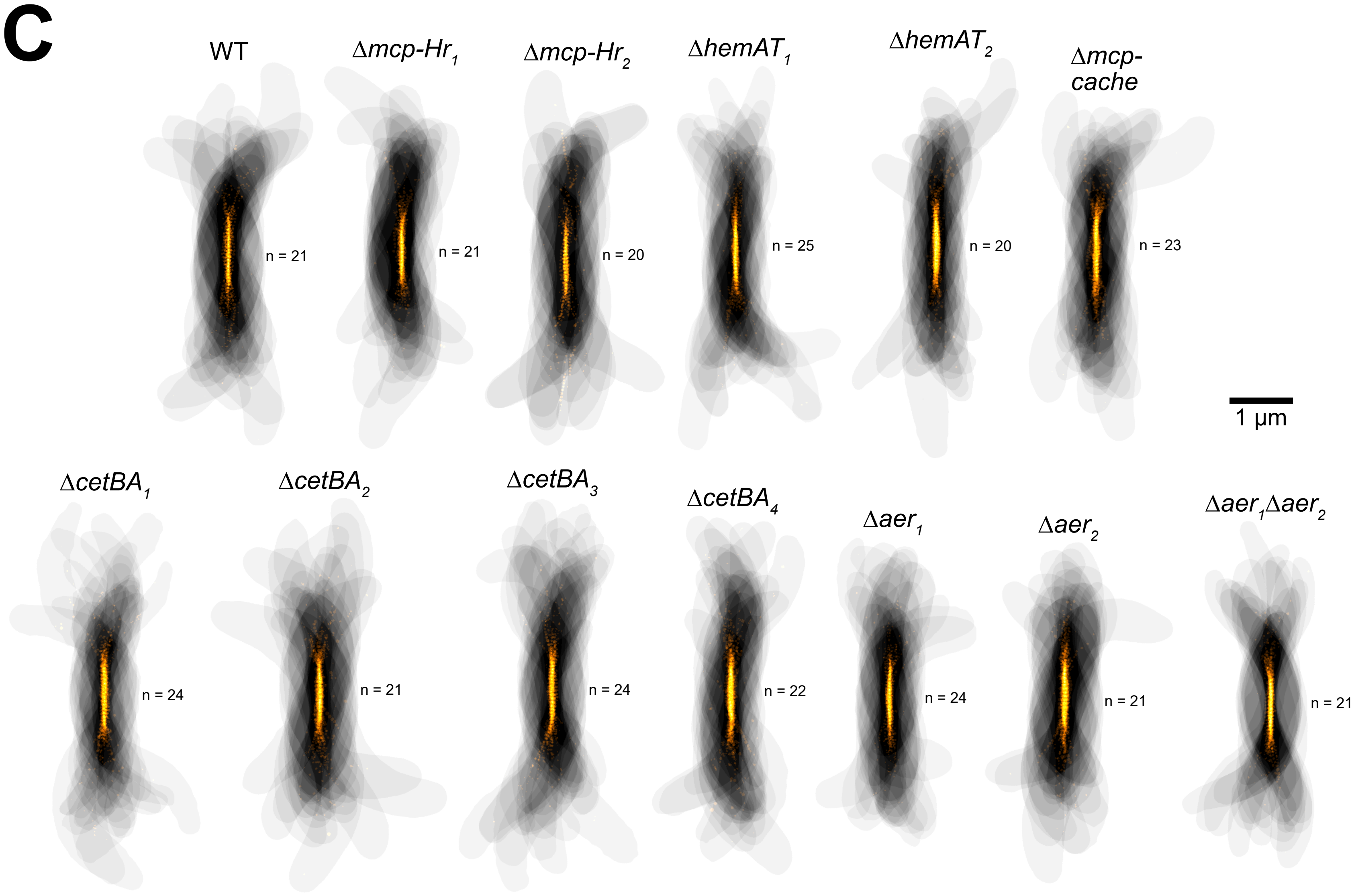

Fig. S7 (continued from previous page). (C) Heatmaps of subcellular magnetosome localization. Magnetosomes are shown in orange and cell bodies in gray. All MCP mutant strains exhibit midcell localization of a contiguous magnetosome chain along the rotational axis of the cell (regions of positive inner curvature), similar to the wild type. The numbers of analyzed cells (n) per strain are indicated in the figure.

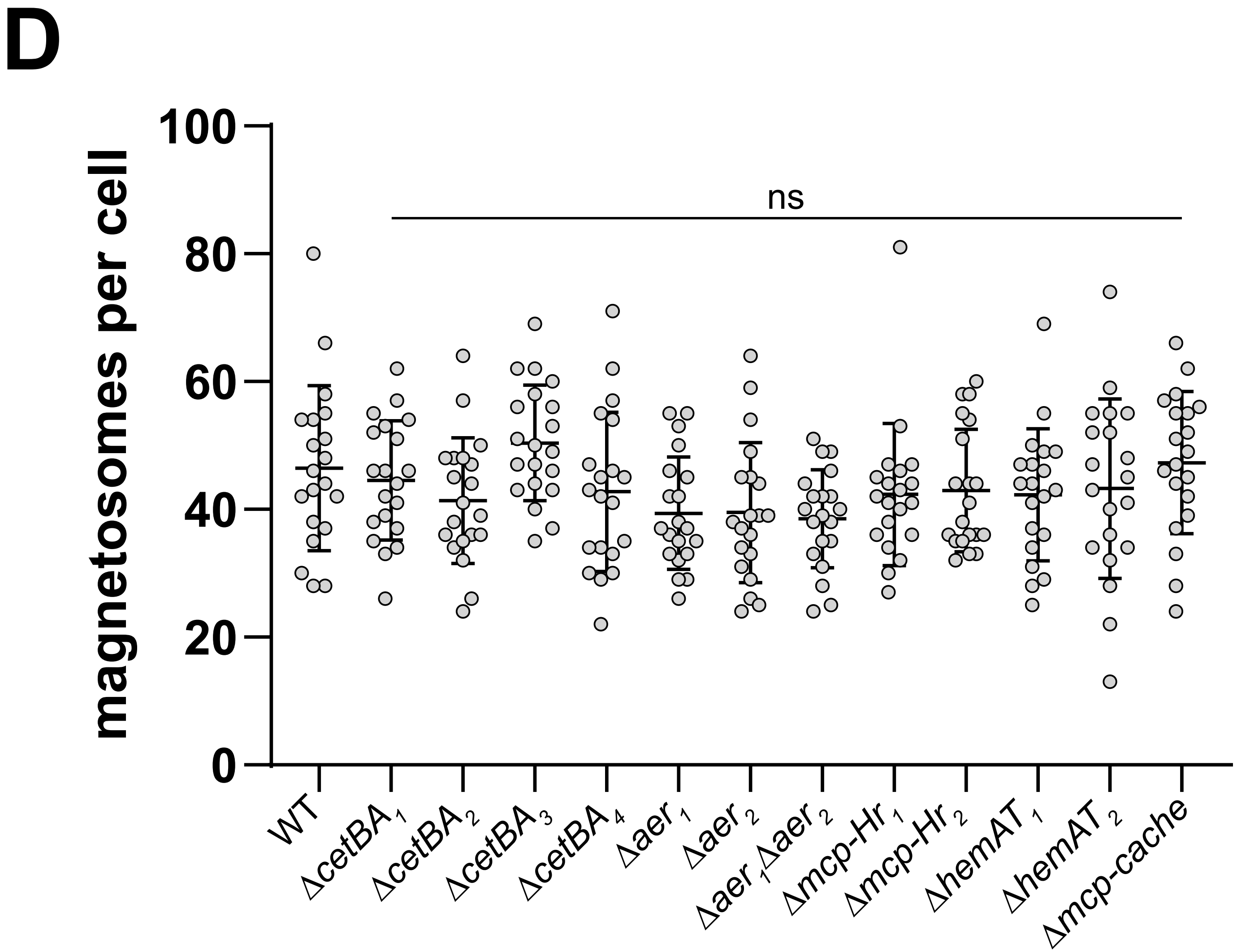

Fig. S7 (continued from previous page). (D) Quantitative analysis of TEM data. The quantification of magnetosome numbers per cell revealed variations between strains, but no significant (ns) differences ($P \geq 0.05$) were observed between MCP deletion strains and the wild type (WT) (Kruskal-Wallis test with Dunn's multiple comparison test). Dots represent individual magnetosome counts (n = 20 cells per strain), centered horizontal lines indicate the mean, and error bars show the SD.

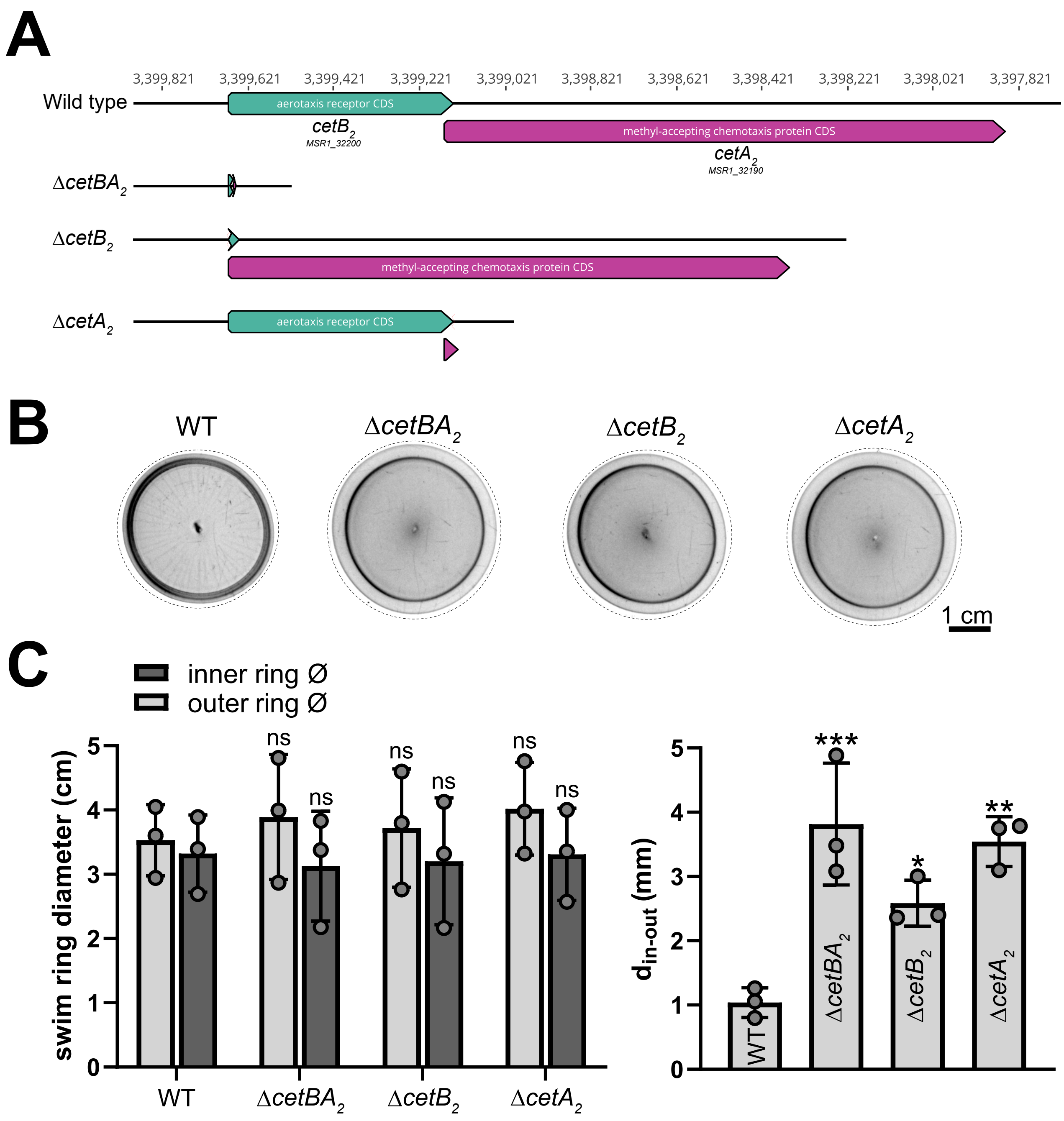

Fig. S8. Effects of individual *cetB*$_2$ and *cetA*$_2$ deletions. (A) Construction of single-gene deletion mutants. To assess the individual roles of *cetB*$_2$ and *cetA*$_2$, single deletions were generated using constructs that were carefully designed to ensure that the respective adjacent gene remains intact (see **Supplemental Material & Methods** and **Table S4** for details). **(B) Swim halo morphologies** and **(C) dimensions.** The Δ*cetB*$_2$ and Δ*cetA*$_2$ strains were compared with the wild type (WT) and the Δ*cetBA*$_2$ strain. Both single deletions produced swim halo phenotypes similar to the Δ*cetBA*$_2$ strain, though the increased distance between inner and outer rings was less pronounced in the Δ*cetB*$_2$ strain. Shown are representative swim halos after 3 days of incubation and their quantified dimensions (n = 3 independent experiments; bars represent the mean, error bars the SD, and dots the individual experimental results). Statistical significance was determined via one-way analysis of variance (ANOVA) with Dunnett's multiple-comparison test against the wild type. *, $P < 0.05$; **, $P < 0.01$; ***, $P < 0.001$; not significant (ns), $P \geq 0.05$. Note: The data presented in this figure are from an independent set of experiments unrelated to **Fig. 1**.

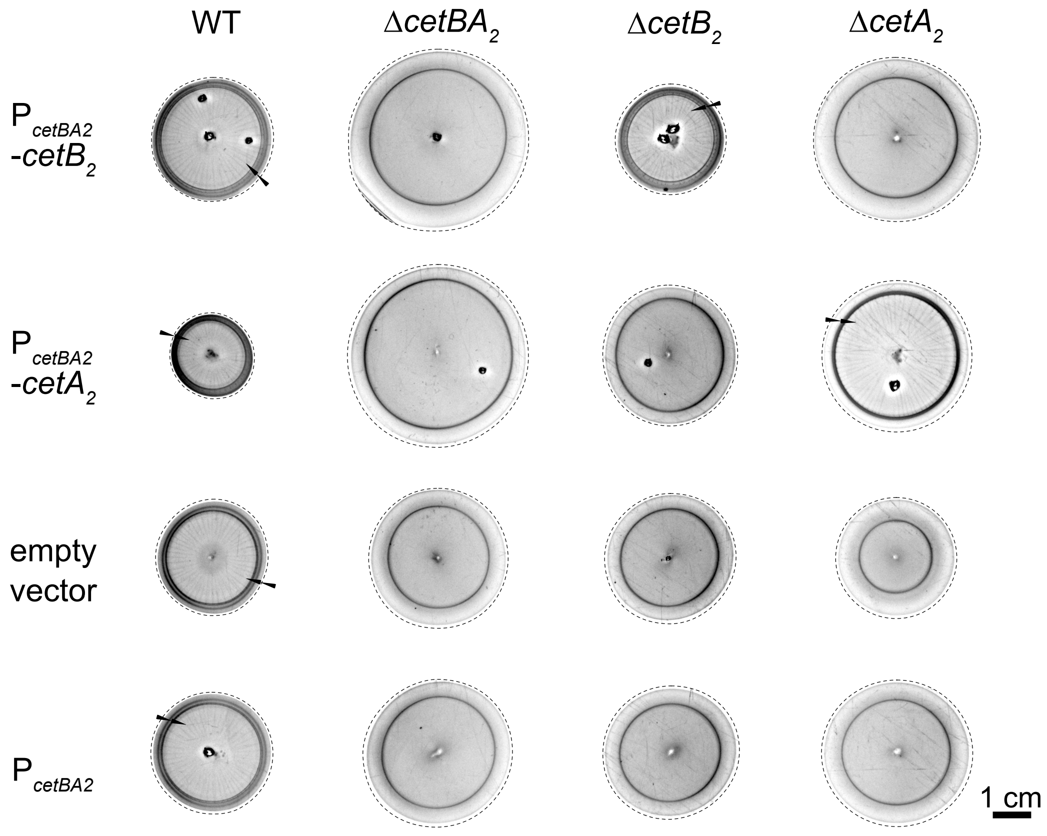

Fig. S9. Transcomplementation of the Δ*cetB*$_2$ and Δ*cetA*$_2$ strains. Deletion mutants lacking *cetB*$_2$ or *cetA*$_2$ were complemented via Tn*7*-based genomic insertion of the respective deleted gene. Complementation is indicated by a reduced distance between inner and outer swim rings and radial stripes (black double arrowheads; other stripe patterns are background). Swim halos (after three days in zero field) are shown for wild-type, Δ*cetBA*$_2$, Δ*cetB*$_2$, and Δ*cetA*$_2$ strains carrying Tn*7*-based *cetB*$_2$ or *cetA*$_2$ expression cassettes including the native *cetBA*$_2$ promoter (P$_{cetBA2}$). Controls include an empty Tn*7* vector and a Tn*7* construct with P$_{cetBA2}$ alone. The upper panel indicates parental strains, while the left-side notations describe the Tn*7* constructs. Representative results from n = 3 analyzed Tn*7* insertion mutants per strain and construct combination are shown.

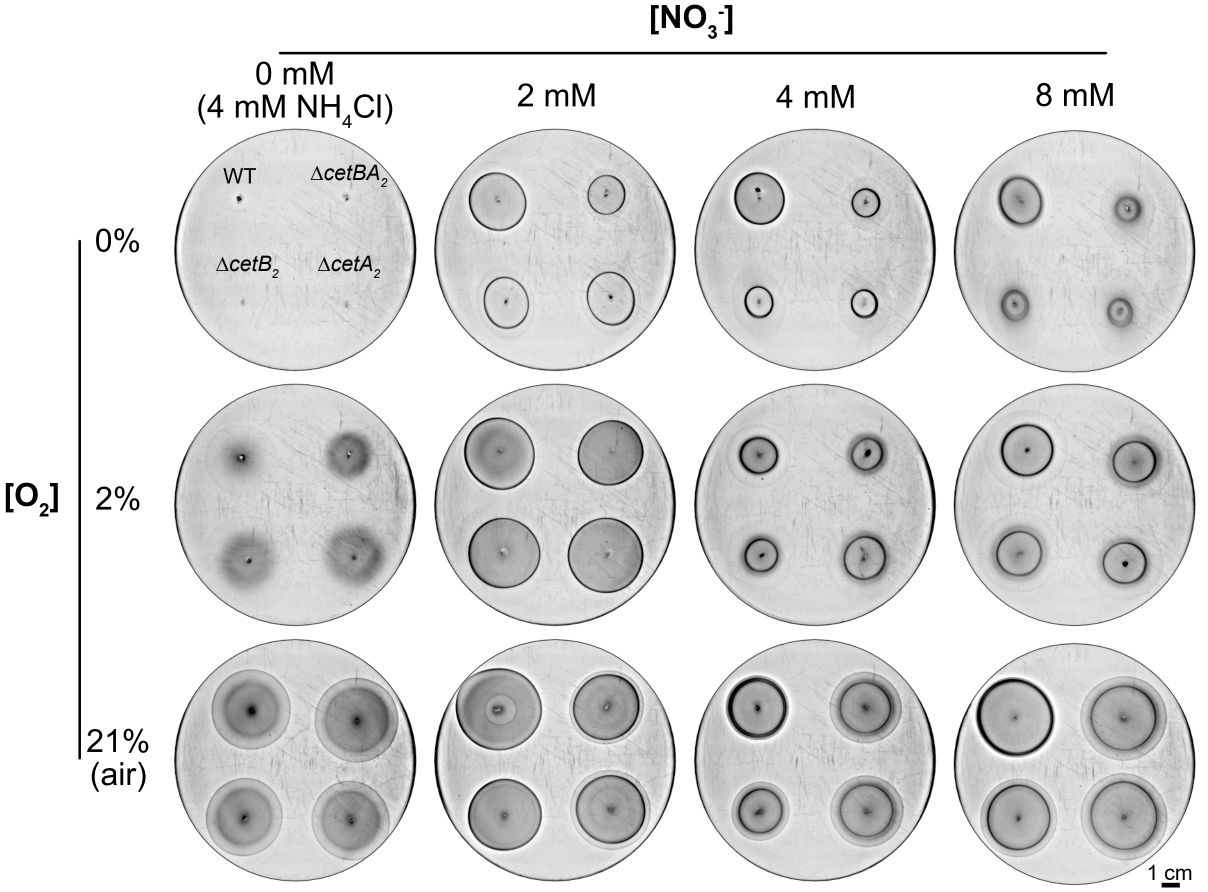

Fig. S10. Impact of nitrate and oxygen on swim halo formation. The wild type (WT) and Δ*cetBA*$_2$, Δ*cetB*$_2$, and Δ*cetA*$_2$ strains (see upper left panel) were inoculated into soft agar plates with varying nitrate concentrations (or 4 mM ammonium chloride instead of nitrate). Plates were then incubated for three days under specified atmospheric oxygen levels. Notably, swim halo morphology is influenced by both nitrate and oxygen, with the phenotypic distinction between the wild type and *cetBA*$_2$ deletion strains most prominent at 21% headspace oxygen and 4 mM nitrate (reflecting the standard conditions used for soft agar assays) or 8 mM nitrate. The pronounced phenotypic differences between the wild type and *cetBA*$_2$ mutants under elevated oxygen and nitrate levels suggest that CetB$_2$ and CetA$_2$ are involved in sensing both terminal electron acceptors, likely as components of metabolism-dependent energy taxis. No growth is observed in the absence of both oxygen and nitrate due to the lack of a terminal electron acceptor. Note that additional stripe patterns, besides the regularly ordered stripes associated with swim halos, are background. Representative micrographs from n = 3 independent experiment repeats are shown.

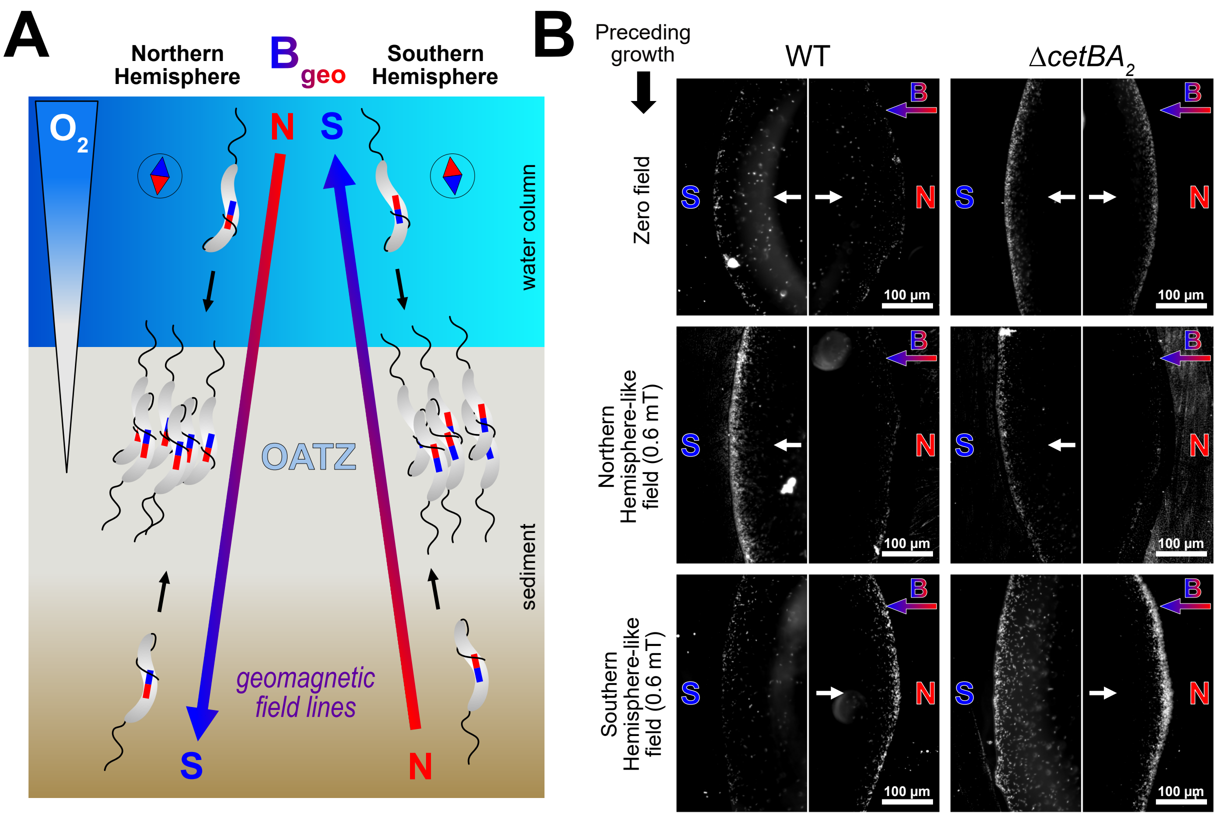

Fig. S11. Deletion of *cetBA*$_2$ does not eliminate swimming polarity. (A) Magnetotactic bacteria (MTB) passively align with the geomagnetic field (B_{geo}), exhibiting opposing inclinations in both hemispheres. Cells on the oxic side exhibit downward swimming toward their favored microhabitat, the 'oxic-anoxic transition zone' (OATZ), while MTB positioned below the OATZ migrate upward. Displacement into zones either below or above optimal oxygen concentrations increases the likelihood of directional reversals. North- and South-seeking magneto-aerotaxis denote the tendency of cells – when exposed to oxygen levels exceeding their optimum – to migrate toward the magnetic south and north poles of a physical magnet, respectively, corresponding to the Earth's North and South (geo)magnetic poles in the Northern and Southern Hemispheres. This behavior is thought to aid in guiding MTB downward into suboxic or anoxic habitats in both hemispheres, in response to oxygen concentrations surpassing their preferred optimum. **(B)** The North- or South-seeking swimming polarity of *M. gryphiswaldense* can be 'selected' through cultivation in oxygen gradients under the influence of a superimposed magnetic field. However, this polar behavior diminishes when no oxygen gradient is present, achieved through culture agitation and aeration. To assess the impact of *cetBA*$_2$ deletion on swimming polarity, the polar behavior of both the wild-type (WT) and Δ*cetBA*$_2$ strains was examined within microdroplets. Microdroplets were subjected to air exposure while placed in a uniform 400 µT magnetic field, following repeated culture passages of non-agitated cultures in either a Northern or Southern Hemisphere-like magnetic field (reflected by a vertical homogeneous 0.6 mT magnetic field produced by coils oriented parallel or antiparallel to the oxygen gradient), or a zero field as a control. White arrows indicate the preferred swimming polarity within the air-exposed microdroplet, wherein migration parallel to the magnetic field signifies a North-seeking swimming polarity, while migration antiparallel to the magnetic field indicates a South-seeking swimming polarity. Swimming polarity selection in the Δ*cetBA*$_2$ mutant was observed in independent repetitions of the experiment. Representative results are shown.

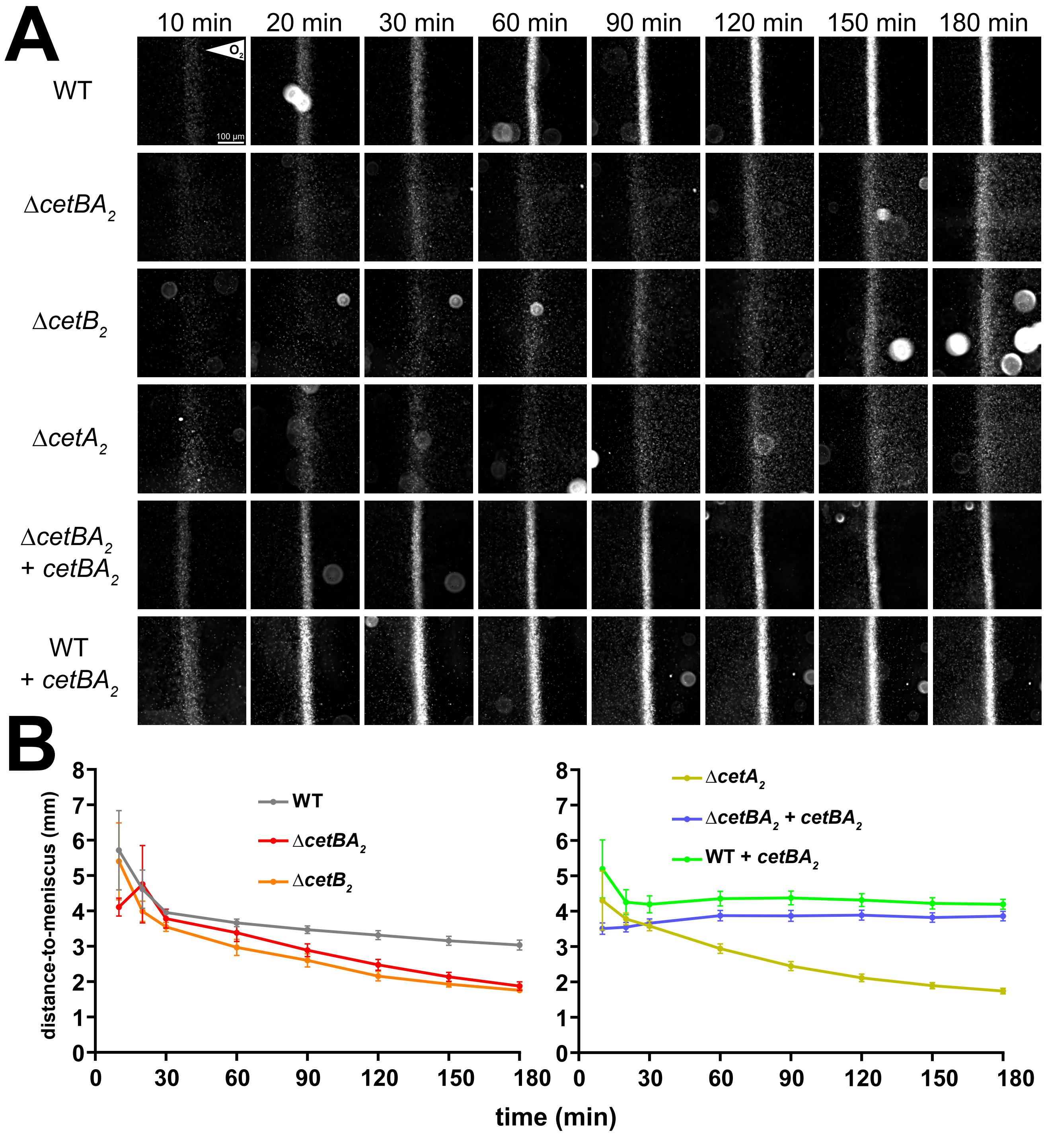

Fig. S12. Spatio-temporal dynamics of aerotactic band formation. (A) Micrographs illustrate the progression of aerotactic band formation over time in the absence of a magnetic field (zero field). The top-left panel illustrates the microscopic scale and direction of the oxygen gradient. **(B)** The mean distance (± SEM) of the aerotactic band from the air-liquid interface (meniscus) was measured at specified time-points. Six microcapillary experiments (n = 6) were conducted per strain. The following strains were analyzed: the wild type (WT), a co-deletion strain of *cetB*$_2$ and *cetA*$_2$ (Δ*cetBA*$_2$), single deletions of *cetB*$_2$ and *cetA*$_2$, and wild-type and Δ*cetBA*$_2$ strains harboring a Tn*7*-based *cetBA*$_2$ expression cassette with the native promoter.

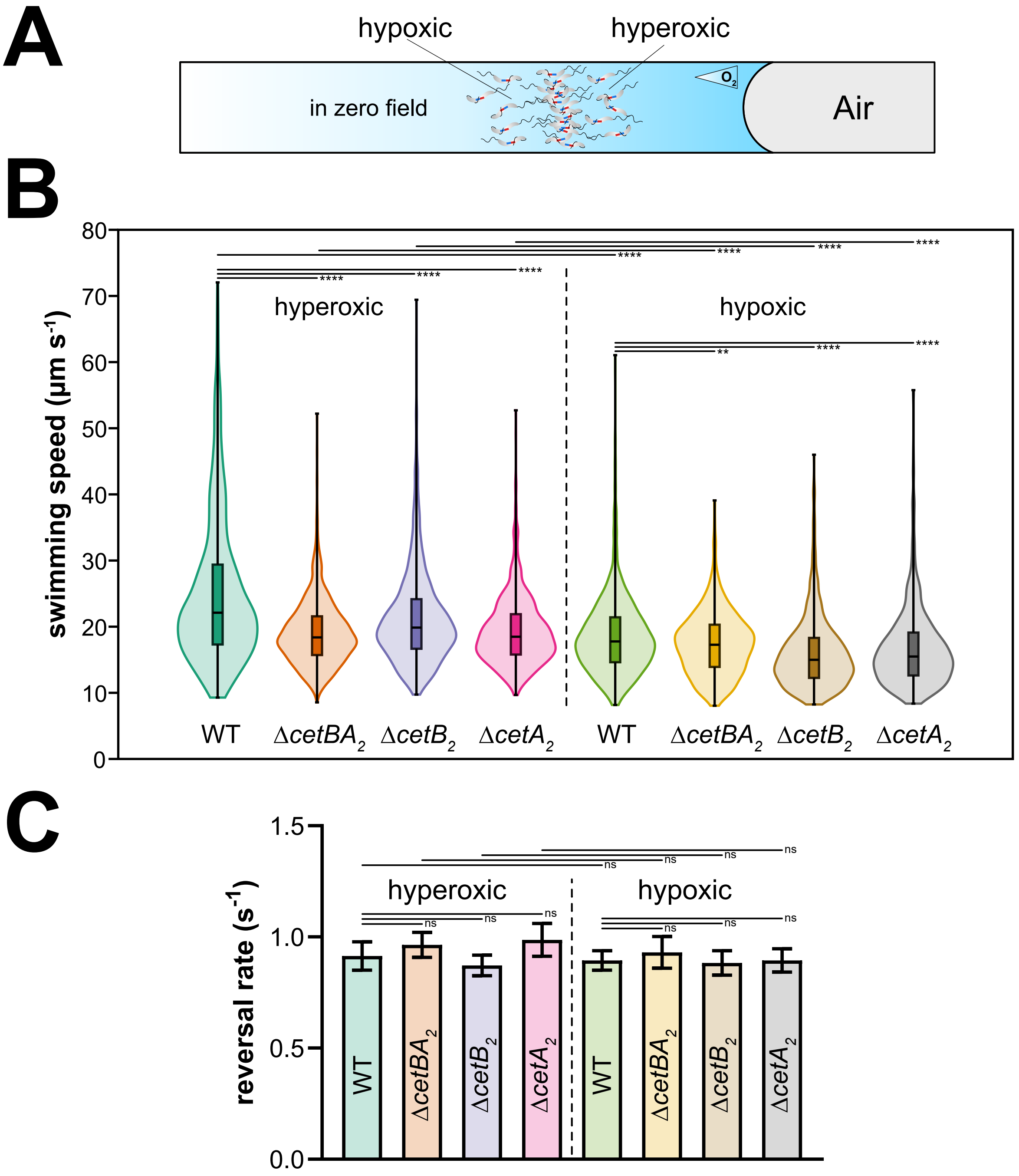

Fig. S13. Behavior of wild-type and *cetBA*$_2$ mutant cells near the aerotactic band. (A) Swimming speeds and reversal rates (after 180 min, under zero-field conditions) of wild-type (WT), Δ*cetBA*$_2$, Δ*cetB*$_2$, and Δ*cetA*$_2$ cells were determined by single-cell tracking in regions in vicinity of the aerotactic band (reflecting oxygen concentrations above and below optimal). **(B)** Distribution of swimming speeds illustrated through Box-violin plots; the median is represented by a horizontal line, the interquartile range by the box, and whiskers extend to the lowest and highest values. The displayed data is derived from averaged swimming speeds obtained from individual single-cell trajectories. **(C)** Reversal rates were manually determined for individual randomly selected single-cell trajectories. The bar chart presents the mean (± SEM) of the 'per track' values. Statistical analysis utilized the Kruskal-Wallis test followed by Dunn's multiple comparison post test; **, $P < 0.01$; ****, $P < 0.0001$; not significant (ns), $P \geq 0.05$. The presented findings are based on n ≥ 3 separate experiments. Cumulative track durations and total track counts are detailed in **Table S2**.

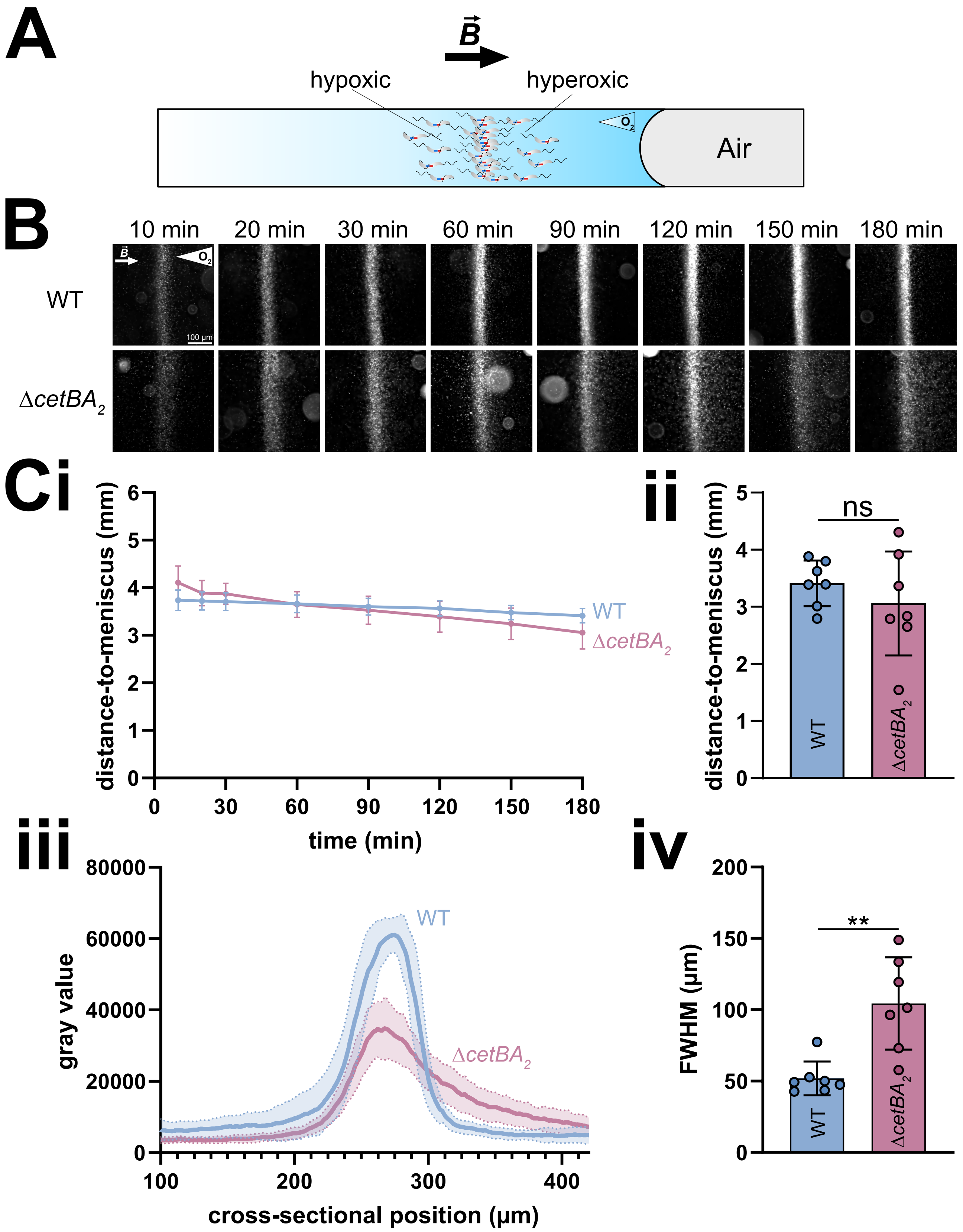

Fig. S14. Spatio-temporal dynamics of aerotactic band formation in a magnetic field. (A) Illustration of the experimental setup and orientation of the magnetic field (400 µT) relative to the oxygen gradient. **(B)** Aerotactic band formation is shown for both the wild type (WT) and the Δ*cetBA*$_2$ strain, illustrating the appearance of the band at the indicated time points. The upper left panel indicates the magnetic field direction, oxygen gradient, and scale. **(Ci)** Mean distance of the aerotactic band from the meniscus (± SEM) over the course of the experiment and **(Cii)** after 180 min (± SD). **(Ciii)** Averaged aerotactic band intensity profiles with shaded areas representing 95% confidence intervals and **(Civ)** mean (± SD) full width at half maximum (FWHM) after 180 min. The data shown are from n = 6 microcapillary experiments per strain. In panels Cii and Civ, dots denote individual experimental results. In both panels, statistical analysis was performed using the Mann-Whitney U test (**, $P < 0.01$; not significant (ns), $P \geq 0.05$). Magnetism of both strains was confirmed prior to aerotactic band formation experiments by assessing their magnetic response.

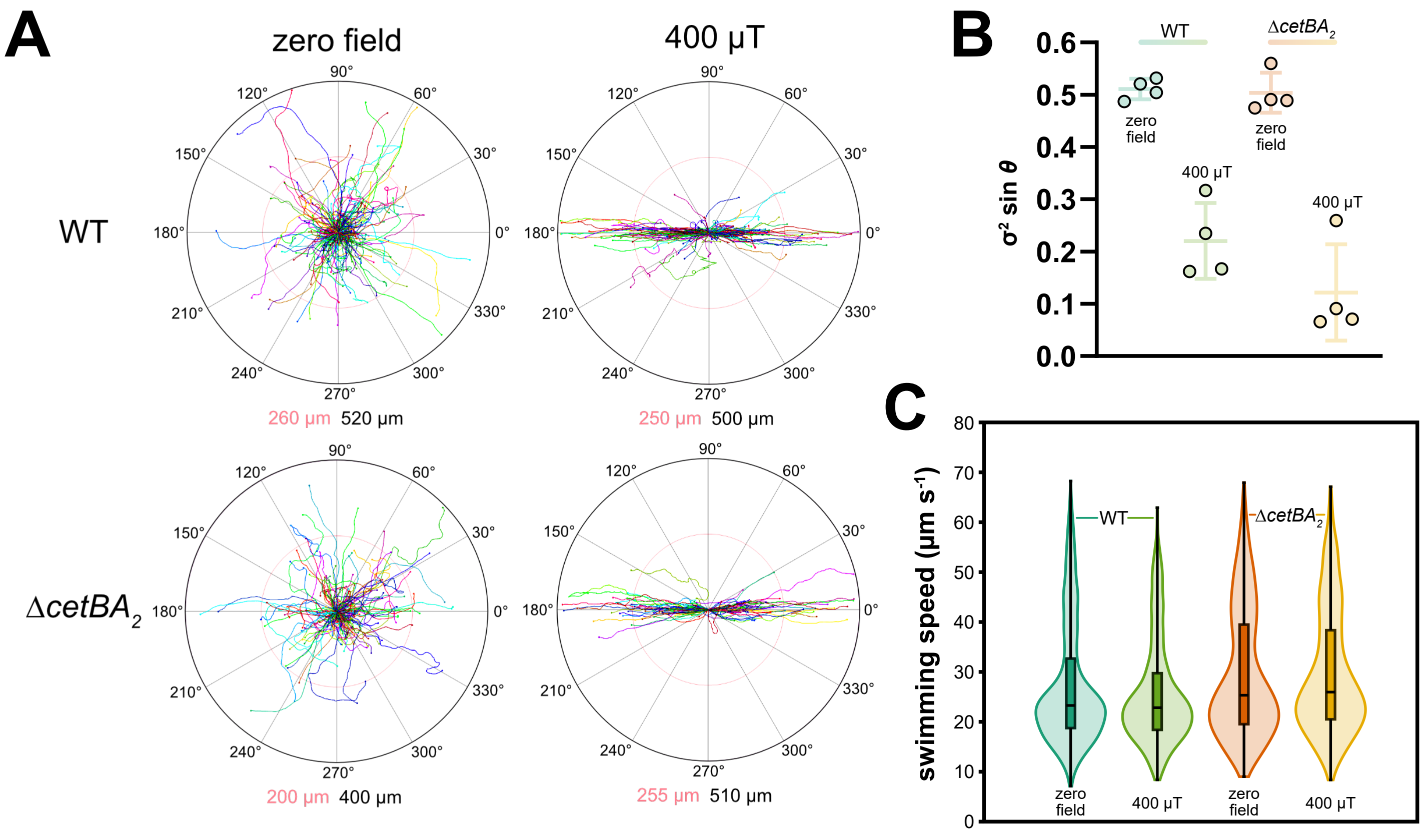

Fig. S15. Single-cell tracking confirms the absence of impaired magnetic alignment in the Δ*cetBA*$_2$ strain. (A) Illustrative swimming trajectories, depicted in a polar graph, showcase the movement patterns of both the wild type (WT) and the Δ*cetBA*$_2$ strain. These trajectories were documented in two conditions: a zero field (where the geomagnetic field was nullified) and a uniform 400 μT magnetic field. The orientation of the applied magnetic field aligns with the horizontal axis of the polar coordinate plot. Magnetic north and south are represented by angles of 0° and 180°, respectively. The track length, denoting the distance covered, is indicated beneath the graphs and is color-coded to match the inner and outer rings of the polar coordinate plot. **(B)** To assess magnetic alignment quantitatively, we computed the variance of sin θ for the population of bacteria based on the average heading angles θ of individual single-cell trajectories. This metric provides a quantification of the dispersion of cell orientations concerning consistent magnetic field lines and is anticipated to decrease with increasing magnetic field strength. In instances where cells exhibit random movement, the variance of sin θ approaches 0.5. Conversely, if cells exhibit alignment with the magnetic field, the value approaches zero, indicating a high degree of alignment. The variance in sin θ was separately computed for n = 4 different tracking experiments. Lines and error bars represent the mean (± SD) of these values. Dots denote individual experimental results. **(C)** Distribution of swimming speeds illustrated through Box-violin plots; the median is represented by a horizontal line, the interquartile range by the box, and whiskers extend to the lowest and highest values. The displayed distribution reflects the entire pooled data from n = 4 different tracking experiments, based on the averaged swimming speeds obtained from hundreds of individual single-cell trajectories. Cumulative track durations and total track counts are detailed in **Table S3**.

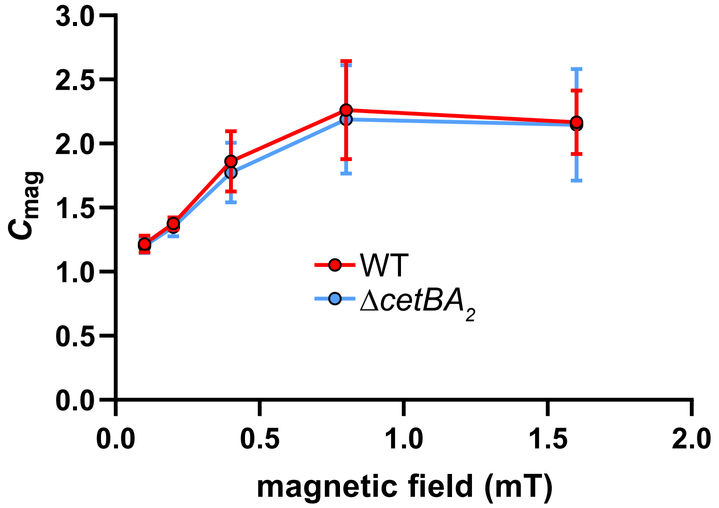

Fig. S16. Magnetic response of the wild-type (WT) and Δ*cetBA*$_2$ strains as a function of the magnetic field. The curves indicate no discernible difference in magnetic alignment between the two strains. Dots and error bars represent the mean and SD from n = 5 separate experiments.

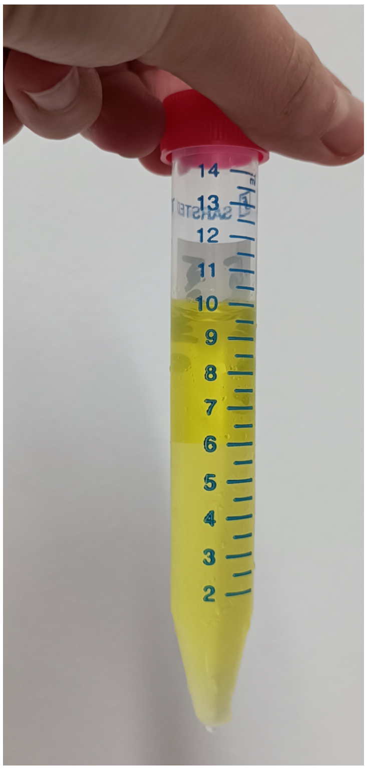

Fig. S17. Purified CetB$_2$. His6-CetB$_2$ displays a strong yellow color, indicative of FAD binding.

A

B

C

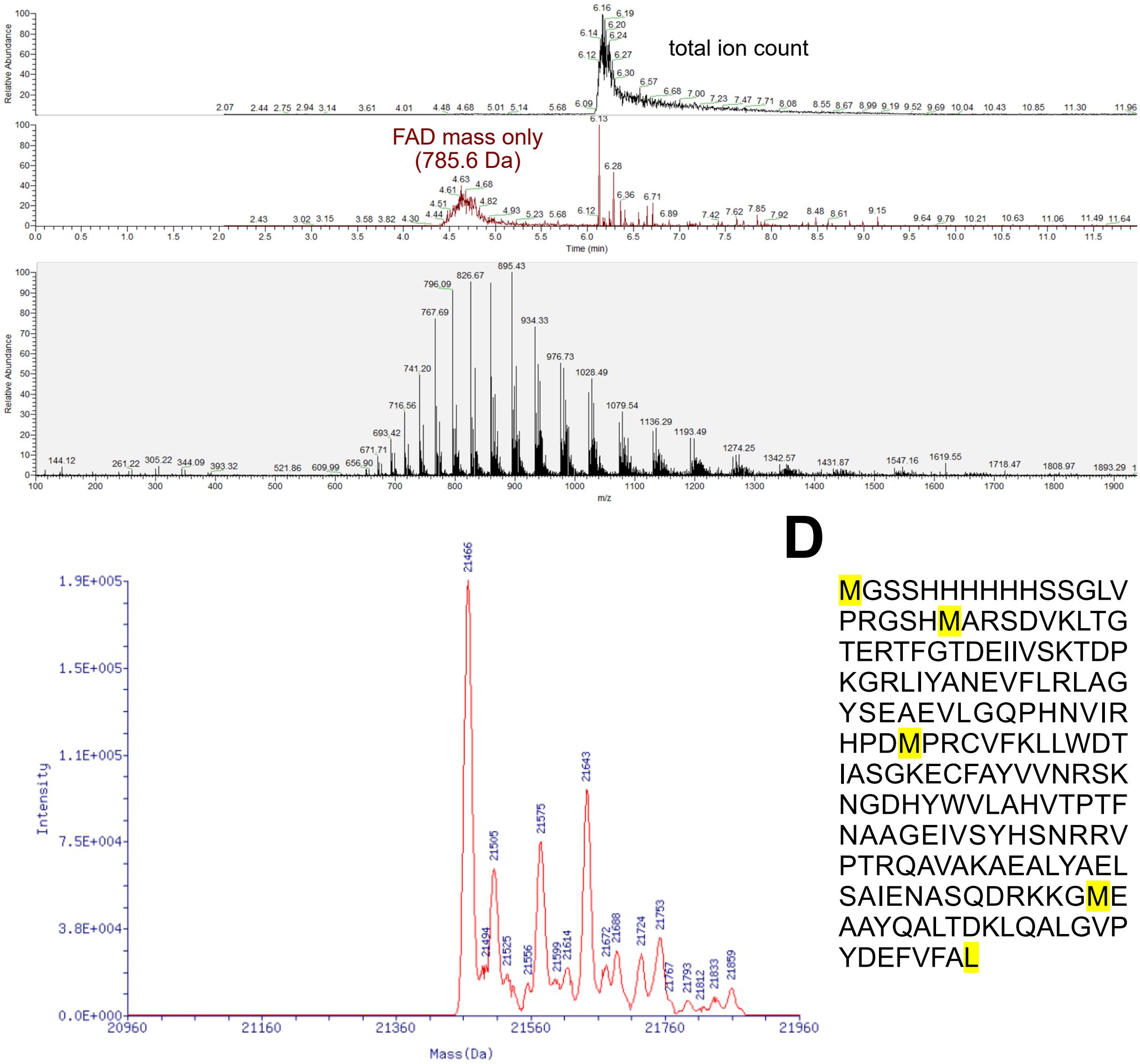

D

MGSSHHHHHHSSGLV
PRGSHMARSDVKLTG
TERTFGTDEIIVSKTDP
KGRLIYANEVFLRLAG
YSEAEVLGQPHNVIR
HPDMPRCVFKLLWDT
IASGKECFAYVVNRSK
NGDHYWVLAHVTPTF
NAAGEIVSYHSNRRV
PTRQAVAKAEALYAEL
SAIENASQDRKKGME
AAYQALTDKLQALGVP
YDEFVFAL

Fig. S18. Liquid Chromatography-Mass Spectrometry (LC-MS) analysis of purified His6-CetB$_2$. (A) Chromatogram, where the x-axis represents retention time and the y-axis represents relative signal intensity. The observation of FAD in the LC-MS analysis, where it elutes before the protein, suggests that FAD was non-covalently bound to CetB$_2$, but released upon chromatographic separation. **(B)** Mass spectrum (for retention time range 6.04-6.38 min) and **(C)** deconvoluted masses. The MS analysis indicates that the protein sample is relatively pure, though it also suggests that the His6-CetB$_2$ protein may have undergone some modifications. Predicted monoisotopic masses for His6-CetB$_2$ and putative modifications are as follows: full length unmodified: 21,586.9 Da; Met deletion: 21,455.9 Da; Leu deletion: 21,473.8 Da; N-acetyl: 21,628.9 Da; Met-oxidation: 21,602.9 Da, 21,618.9 Da, 21,634.9 Da, and 21,650.9 Da. **(D)** Amino acid sequence of His6-CetB$_2$, with potential modification sites highlighted in yellow.

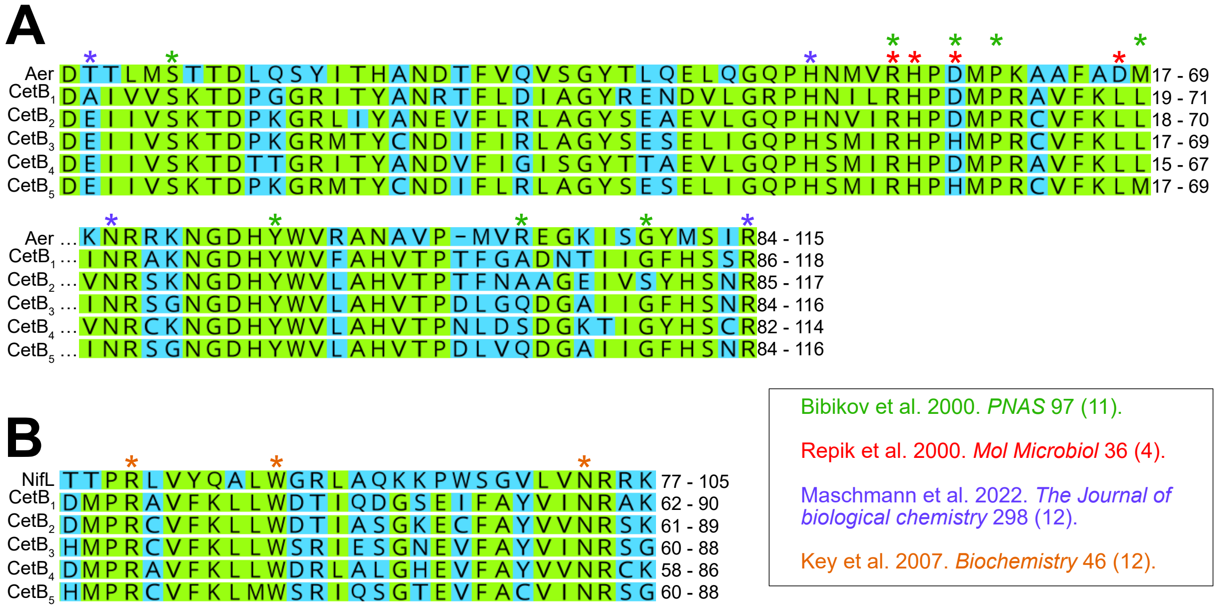

Fig. S19. Conservation of FAD-binding amino acid residues in CetB$_2$. Protein sequence alignments of **(A)** *E. coli* Aer (b3072) and **(B)** *Azotobacter vinelandii* NifL (AvCA_50990) to *M. gryphiswaldense* CetB$_1$-CetB$_5$, displaying selected regions of their PAS domains. Green coloring indicates 80% to 100% sequence similarity, whereas blue refers to a similarity of <80%. Amino acids in NifL and Aer, previously implicated in FAD binding, are marked by asterisks. The asterisk color indicates the respective study. Sequence alignments suggest that Ser23, His54, Arg58, His59, Asp61, Pro63, Arg64, Trp71, Asn86, Tyr94, and Arg117 in CetB$_2$ may be involved in FAD binding. Notably, these residues partially differ from those identified in the structural model of CetB$_2$ (**Fig. 7Aiii** and **Fig. S21B**), indicating that not all previously reported residues necessarily interact with FAD in CetB$_2$.

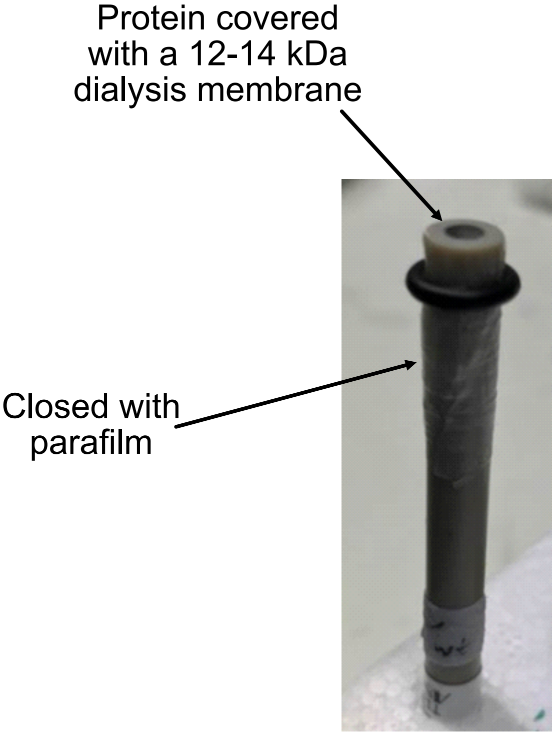

Fig. S20. Experimental setup for electrochemical measurements. Electrode preparation involved applying the protein sample onto a glassy-carbon electrode. After drying, a dialysis membrane was secured over the electrode with an O-ring and parafilm to prevent protein leaching.

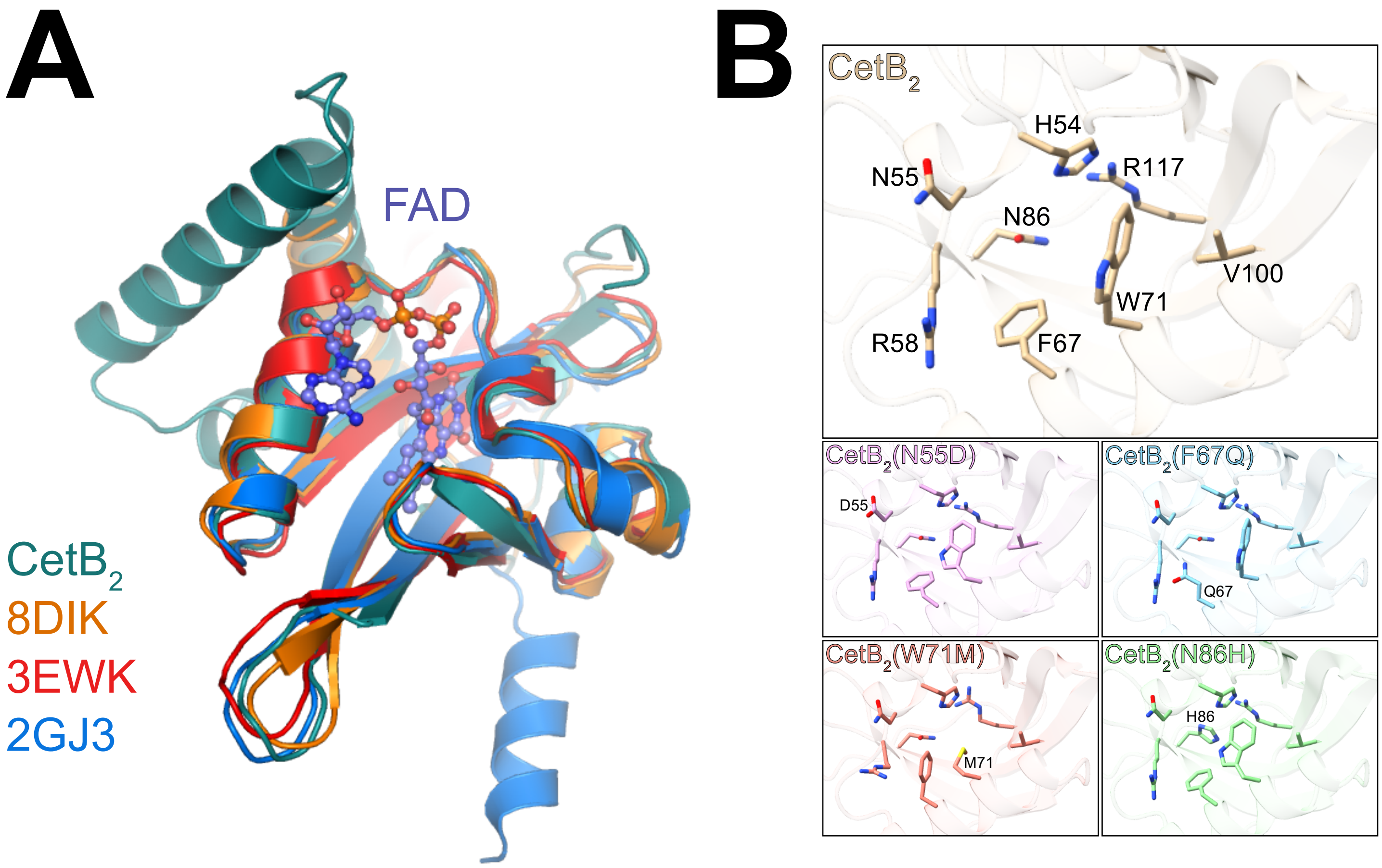

Fig. S21. (A) CetB$_2$ and homologs. The predicted CetB$_2$ structure (deep teal) is superimposed with the PAS domains of the *E. coli* Aer aerotaxis receptor (8DIK in gold), *Azotobacter vinelandii* NifL (2GJ3 in light blue), and *Methylococcus capsulatus* MmoS (3EWK in red). Proteins are represented as cartoon, and the docked FAD is shown as a ball-and-stick model. The comparison of CetB$_2$ with the other domains using the Foldseek server revealed the following E-values, sequence identities, and RMSD values: 8DIK (1.60e^{-14}, 41.2, 0.88), 2GJ3 (1.27e^{-10}, 33.9, 1.77), and 2EWK (8.04e^{-10}, 32.5, 5.6). **(B) Experimentally tested amino acid replacements in the CetB$_2$ FAD-binding pocket.** AlphaFold2 predictions for these variants revealed no relevant changes in the overall protein fold, but some alterations in the orientation of other predicted FAD-binding residues. All variants, with side chains of FAD-binding residues, are shown in comparison to the wild-type CetB$_2$ FAD-binding pocket (displayed in the first top panel).

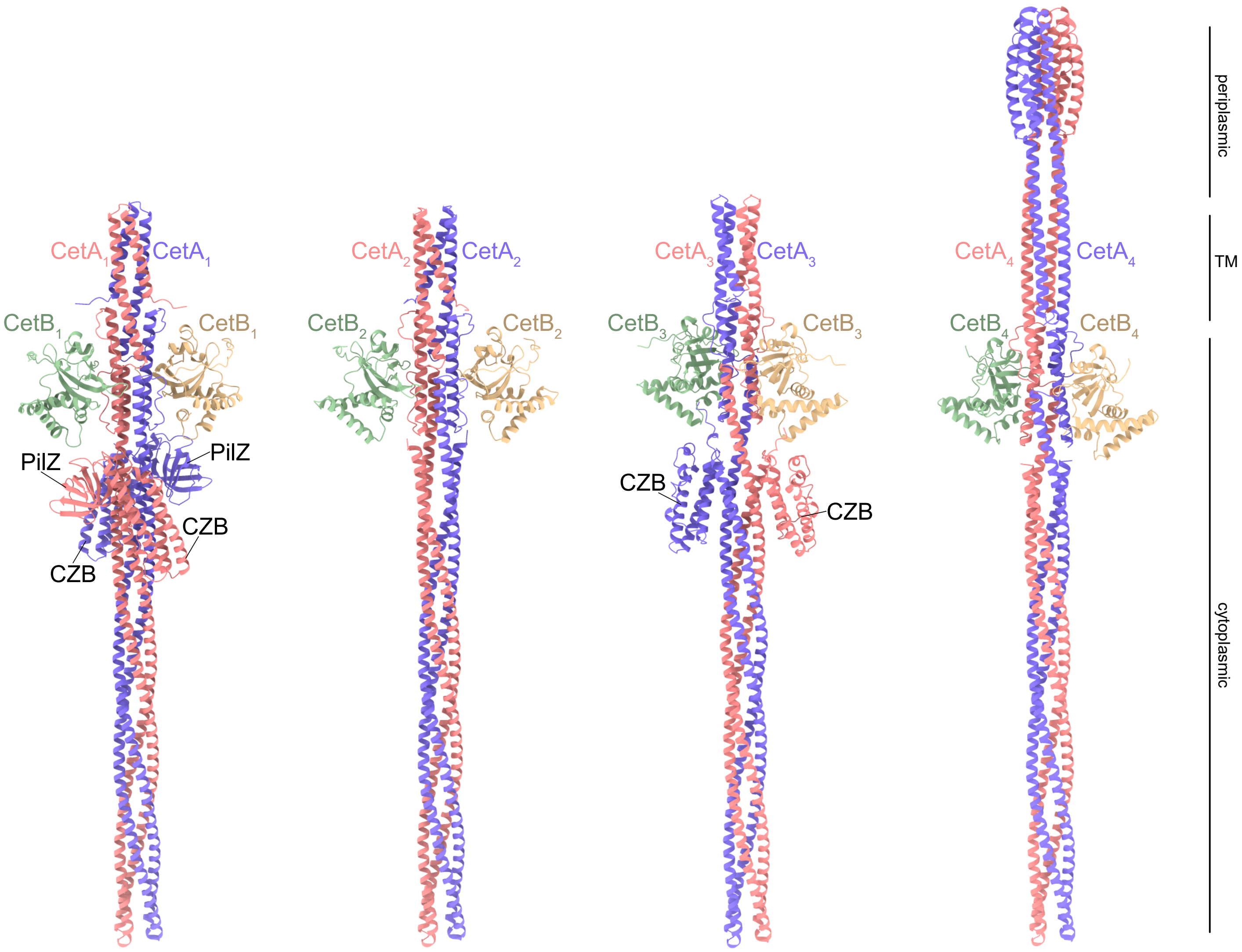

Fig. S22. Structural models (AlphaFold2) of bipartite chemoreceptor complexes from *M. gryphiswaldense*. Additional putative cytoplasmic sensory domains in $CetA_1$ and $CetA_3$ are positioned adjacent to receptor-bound $CetB_1$ and $CetB_3$, respectively, including the c-di-GMP-binding PilZ domain and the chemoreceptor zinc-binding (CZB) domain. $CetA_4$ appears to have an additional periplasmic sensing domain, which has no related hits in the InterProScan database. TM = transmembrane helices.

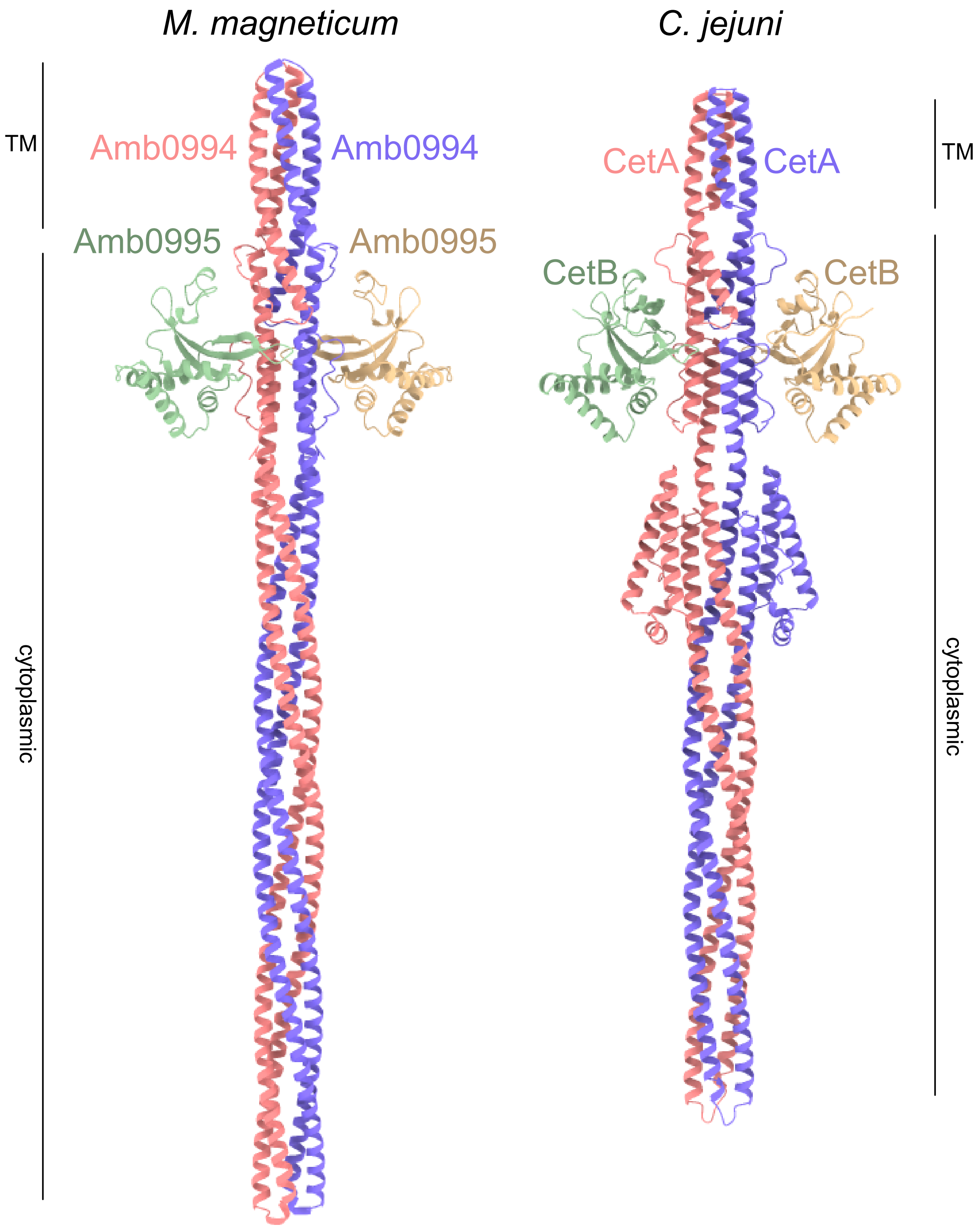

Fig. S23. Structural models (AlphaFold2) of bipartite chemoreceptor complexes from *M. magneticum* and *C. jejuni*. Amb0994 and Amb0995 from *M. magneticum* show 42.1% and 54.7% amino acid identity, respectively, compared to *M. gryphiswaldense* $CetA_2$ and $CetB_2$. In contrast, CetA and CetB from *C. jejuni* (strain ATCC 700819) exhibit 23.6% and 40.4% amino acid identity, respectively, to *M. gryphiswaldense* $CetA_2$ and $CetB_2$.

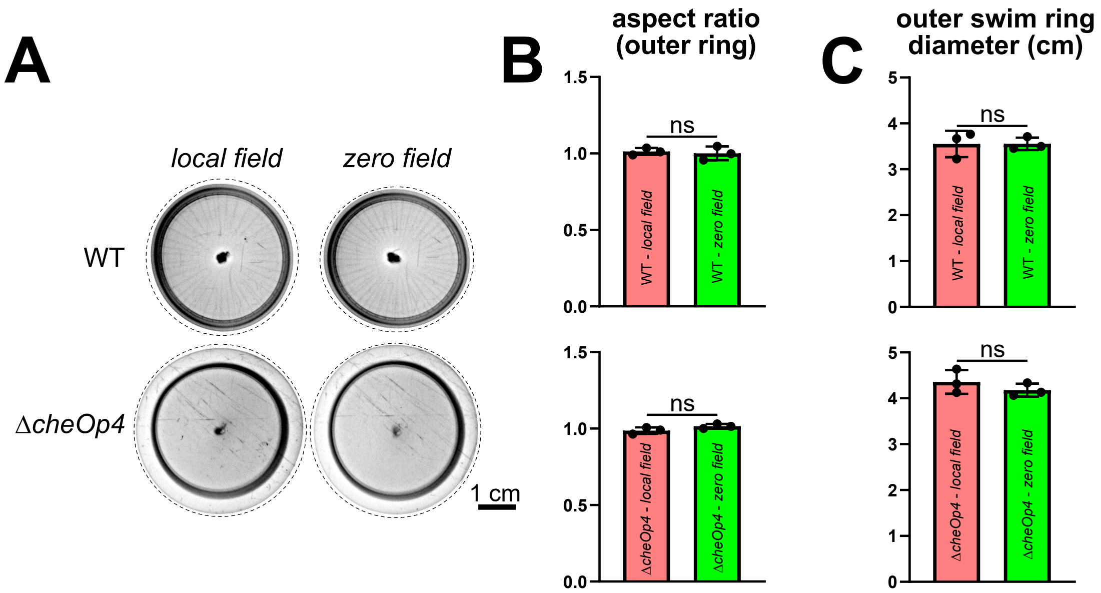

Fig. S24. Impact of the local geomagnetic field on aerotactic swim halo formation. Soft agar plates were incubated for three days simultaneously under the influence of the local geomagnetic field and within a Zero Gauss Chamber (2-G Enterprises). **(A) Swim halo morphology:** No discernible differences in swim halo morphology were observed between the local geomagnetic field and zero-field condition. **(B) Swim halo aspect ratio:** The small difference in the absolute values of the north (X; 16.1 to 18.8 μT) and east (Y; -2 to -4.8 μT) components of the horizontal intensity of the local geomagnetic field did not result in significant distortion of swim halos. **(C) Outer swim halo diameter:** In this specific experiment, the vertical intensity (Z) of the local magnetic field (26 - 47.5 μT) exhibited no substantial impact on the size of swim halos. The experiment involved triplicate cultures (n = 3) of both wild-type (WT) and Δ*cheOp4* strains, with C_{mag} values of 1.29 ± 0.03 and 1.29 ± 0.06, respectively (mean ± SD). Bar charts represent the mean, error bars indicate the SD, and dots represent individual outcomes. Statistical analysis, conducted via t-test, revealed non-significant differences (ns). The local magnetic field was measured using a factory-calibrated GM08 Gauss meter equipped with a TP002HS high-sensitivity transverse probe (Hirst Magnetic Instruments). The provided values reflect the range of measured local geomagnetic field intensities, aligning with computed intensities using the current *International Geomagnetic Reference Field* (https://www.ngdc.noaa.gov/geomag/magfield.shtml) for Bayreuth (Latitude: 49° 56' 52.15" N; Longitude: 11° 34' 44.15" E; corresponding to north, east, and vertical components of 20.55 μT, -1.21 μT, and 43.69 μT, respectively).

Table S1. Pairwise sequence identity (above the diagonal of 100% values) and similarity (below the diagonal) calculated with SIAS (http://imed.med.ucm.es/Tools/sias.html) based on multiple-sequence alignments of *M. gryphiswaldense* CetA **(A)** and CetB **(B)** paralogs. Multiple-sequence alignments were generated with Geneious Prime® 2023.0.4 (Global alignment with free end gaps, BLOSUM62 similarity matrix). A fifth CetB paralog (MSR1_04030, $CetB_5$) lacking a corresponding CetA partner was included in the alignment.

**A** Identity / Similarity	**CetA$_4$**	**CetA$_3$**	**CetA$_2$**	**CetA$_1$**
**CetA$_4$**	100%	22.41%	37.38%	21.73%
**CetA$_3$**	29.18%	100%	29.81%	25.08%
**CetA$_2$**	44.49%	39.9%	100%	33.94%
**CetA$_1$**	29.09%	33.27%	43.8%	100%

**B** Identity / Similarity	**CetB$_5$**	**CetB$_4$**	**CetB$_3$**	**CetB$_2$**	**CetB$_1$**
**CetB$_5$**	100%	48.48%	71.51%	52.12%	45.45%
**CetB$_4$**	55.75%	100%	59.53%	58.95%	49.13%
**CetB$_3$**	73.93%	68.2%	100%	63.58%	47.97%
**CetB$_2$**	58.18%	67.05%	70.52%	100%	51.72%
**CetB$_1$**	52.12%	57.8%	55.49%	59.19%	100%

Table S2. Single-cell tracking of wild-type and *cetBA*$_2$ mutant cells in vicinity of the aerotactic band. (A) Swimming speeds and **(B)** reversal rates (in zero field after 180 min) of wild-type, Δ*cetBA*$_2$, Δ*cetB*$_2$, and Δ*cetA*$_2$ cells in regions surrounding the aerotactic band (reflecting oxygen concentrations above and below optimal). **(Ai)** The mean swimming speed (± SEM) was calculated based on the averaged swimming speeds of individual single-cell trajectories from all experiments . **(Aii)** The average swimming speed was calculated separately for each experiment, and the provided value represents the mean (± SD) of the calculated average speeds. **(Bi)** The reversal rate was calculated by dividing the sum of all detected reversal through the sum of the temporal length of all individual tracks from all experiments. **(Bii)** The reversal rate was calculated per track, the value given represents the average (± SEM) of the per track values from all experiments.

(A) Swimming speeds

strain	redox regime	swimming speed (µm s^{-1})		total duration of analyzed tracks (s)	total number of analyzed tracks	number of experiments
		(i) pooled results from all experiments	(ii) per experiment			
WT	hyperoxic	25.1 ± 0.37	24.0 ± 3.07	1804	937	4
	hypoxic	18.6 ± 0.17	17.5 ± 1.97	2339	1271	4
Δ*cetBA*$_2$	hyperoxic	19.1 ± 0.09	18.9 ± 0.56	4980	2911	3
	hypoxic	17.5 ± 0.16	17.1 ± 0.77	1829	848	3
Δ*cetB*$_2$	hyperoxic	21.3 ± 0.20	21.2 ± 2.21	2164	1307	3
	hypoxic	16.0 ± 0.19	16.3 ± 1.19	1631	866	3
Δ*cetA*$_2$	hyperoxic	19.3 ± 0.15	19.2 ± 1.33	1946	1241	3
	hypoxic	16.7 ± 0.22	16.4 ± 1.17	1383	736	3

(B) Reversal rates

strain	redox regime	reversal rate (s^{-1})		total number of reversal events	total duration of analyzed tracks (s)	total number of analyzed tracks	number of experiments
		(i) based on all tracks	(ii) per track				
WT	hyperoxic	0.945	0.914 ± 0.064	66	70	33	4
	hypoxic	0.887	0.895 ± 0.044	60	68	29	4
Δ*cetBA*$_2$	hyperoxic	0.945	0.965 ± 0.056	87	92	44	3
	hypoxic	0.862	0.931 ± 0.071	115	133	44	3
Δ*cetB*$_2$	hyperoxic	0.866	0.872 ± 0.046	55	64	34	3
	hypoxic	0.863	0.883 ± 0.055	86	100	36	3
Δ*cetA*$_2$	hyperoxic	0.973	0.986 ± 0.074	48	49	30	3
	hypoxic	0.887	0.894 ± 0.052	54	61	27	3

Table S3. Single-cell tracking of the wild type and the Δ*cetBA₂* strain for the investigation of magnetic alignment. Experiments were conducted in a standard wet mount under uncontrolled atmospheric conditions. **(A)** To quantify magnetic alignment, we calculated the variance in sin *θ* among a population of bacteria, using the average heading angles *θ* of their individual swimming trajectories. When cells move randomly, the variance in sin *θ* approaches 0.5. Conversely, alignment with the magnetic field leads to a value nearing zero. **(Ai)** The variance in sin *θ* was computed based on the average *θ* values of individual tracks across all experiments. **(Aii)** The variance in sin *θ* was determined per experiment, with the presented value representing the mean (± SD) of these calculations. **(B)** Additionally, mean swimming speeds for bacterial populations were computed based on the averaged swimming speeds of individual single-cell trajectories. **(Bi)** The mean swimming speed (± SEM) was calculated based on the averaged values of individual tracks from all experiments. **(Bii)** The mean swimming speed was calculated per experiment, with the provided value representing the mean (± SD) of the calculated average speeds per experiment.

strain	magnetic field	(A) magnetic alignment (σ^2 sin *θ*)		(B) swimming speed (µm s^{-1})		total duration of analyzed tracks (s)	total number of analyzed tracks	number of experiments
		(i) pooled results from all experiments	(ii) per experiment	(i) pooled results from all experiments	(ii) per experiment			
WT	zero field	0.506	0.511 ± 0.020	27.2 ± 0.36	26.7 ± 3.70	5274	1137	4
	400 µT	0.262	0.220 ± 0.073	25.7 ± 0.36	26.0 ± 1.49	4020	864	4
Δ*cetBA₂*	zero field	0.487	0.504 ± 0.038	29.4 ± 0.38	27.7 ± 4.64	6584	1139	4
	400 µT	0.130	0.122 ± 0.092	29.7 ± 0.47	29.6 ± 4.83	5403	686	4

Table S4. Strains and plasmids. *E. coli* strains with plasmids and *M. gryphiswaldense* strains with Tn-insertions are not individually listed.

**Strain or vector**	**Relevant characteristic(s)**	**Reference and/or source**
**Strains**		
E. coli		
DH5α	Host for cloning; F^- φ80*lacZ*ΔM15 Δ(*lacZYA-argF*)U169 *recA1 endA1 hsdR17*(r_K^- m_K^+) *phoA supE44* λ^- *thi-1 gyrA96 relA1*	[34]
WM3064	Conjugation strain; *thrB1004 pro thi rpsL hsdS lacZ*ΔM15 *RP4-1360* Δ(*araBAD*)*567* Δ*dapA1341*::[*erm pir*]	William Metcalf at UIUC
BTH101	Two-hybrid reporter strain; F^- *cya-99 araD139 galE15 galK16 rpsL1* (*Str*^{R}) *hsdR2 mcrA1 mcrB1*	Euromedex
Rosetta™(DE3) pLysSRARE	Expression strain; F^- *ompT hsdS*$_B$(r_B^- m_B^-) *gal dcm* (DE3) pLysSRARE (Cam^R)	Novagen
M. gryphiswaldense		
Wild type (WT)	MSR-1 R3/S1; Rif^R, Sm^R	[35]
Δ*cheOp1*	Chemotaxis operon 1 deletion strain	[15]
Δ*cheOp4*	Chemotaxis operon 4 deletion strain	[15]
Δ*aer*$_1$	*MSR1_08650* deletion strain	This study
Δ*aer*$_2$	*MSR1_26020* deletion strain	This study
Δ*aer*$_1$Δ*aer*$_2$	*MSR1_08650* & *MSR1_26020* co-deletion strain	This study
Δ*cetBA*$_1$	*MSR1_00130* & *MSR1_00120* co-deletion strain	This study
Δ*cetBA*$_2$	*MSR1_32200* & *MSR1_32190* co-deletion strain	This study
Δ*cetA*$_2$	*MSR1_32190* deletion strain	This study
Δ*cetB*$_2$	*MSR1_32200* deletion strain	This study
Δ*cetBA*$_3$	*MSR1_19630* & *MSR1_19620*	This study

	co-deletion strain	
$\Delta cetBA_4$	*MSR1_04420* & *MSR1_04410* co-deletion strain	This study
$\Delta hemAT_1$	*MSR1_09310* deletion strain	This study
$\Delta hemAT_2$	*MSR1_02260* deletion strain	This study
$\Delta mcp\text{-}Hr_1$	*MSR1_18100* deletion strain	This study
$\Delta mcp\text{-}Hr_2$	*MSR1_27430* deletion strain	This study
Δ*mcp-cache*	*MSR1_02290* deletion strain	This study
Δ*flaA*	*flaA* deletion strain	This study (strain initially described by Schultheiss et al.[35], regenerated through site-specific deletion)
**Plasmids**		
pORFM	universal in-frame deletion/in-frame fusion vector for GalK-based counterselection; *npt* P_{tet}-*galK* *tetR* *mob*RK2	[1]
pORFM-Δaer_1	*MSR1_08650* (*MGR_0403*) deletion vector; homologous arms were amplified with primers 120/121 and 122/123	This study
pORFM-$\Delta cetBA_1$	*MSR1_00120-30* (*MGR_0748-49*) co-deletion vector; homologous arms were amplified with primers 239/240 and 241/242	This study
pORFM-$\Delta cetBA_2$	*MSR1_32190-200* (*MGR_1772/MGR_1707*) co-deletion vector; homologous arms were amplified with primers 245/246 and 247/248	This study
pORFM-$\Delta cetA_2$	*MSR1_32190* (*MGR_1707*) deletion vector; homologous arms were amplified with primers 592/593 and 594/248	This study
pORFM-$\Delta cetB_2$	*MSR1_32200* (*MGR_1772*) deletion vector; homologous arms were amplified with primers 245/587 and 588/589	This study
pORFM-$\Delta cetBA_3$	*MSR1_19620-30* (*MGR_2563-64*) co-deletion vector; homologous arms were amplified with primers 251/252 and 253/254	This study
pORFM-$\Delta cetBA_4$	*MSR1_04410-20* (*MGR_4201-02*) co-deletion vector; homologous arms were amplified with primers 257/258 and 259/260	This study

pORFM-Δ*hemAT*$_1$	*MSR1_09310* (*MGR_2126*) deletion vector; homologous arms were amplified with primers 263/264 and 265/266	This study
pORFM-Δ*hemAT*$_2$	*MSR1_02260* (*MGR_3994*) deletion vector; homologous arms were amplified with primers 269/270 and 271/272	This study
pORFM-Δ*mcp-Hr*$_1$	*MSR1_18100* (*MGR_1492*) deletion vector; homologous arms were amplified with primers 126/127 and 128/129	This study
pORFM-Δ*mcp-Hr*$_2$	*MSR1_27430* (*MGR_2868*) deletion vector; homologous arms were amplified with primers 132/133 and 134/135	This study
pORFM-Δ*flaA*	*flaA* deletion vector; homologous arms were amplified with primers 74/75 and 76/77	This study
pFM271e_1	universal in-frame deletion/in-frame fusion vector for GalK-based counterselection; *npt* P_{lac}-*galK lacI mob*RK2	[2]
pFM271e_1-Δ*mcp-cache*	*MSR1_02290* (*MGR_3997*) deletion vector; homologous arms were amplified with primers KTS330/KTS331 and KTS332/KTS333	This study
pCM184	allelic exchange vector; Ap^R, Kn^R, Tc^R; *oriT;* ColE1 *ori; loxP*	[3]
pCM184-Δ*aer*$_2$ (pFP06i)	*MSR1_26020* (*MGR_3404*) deletion vector; homologous arms were amplified with primers FP58/FP16 and FP59/FP66	This study
pBAMII-Tn*7*	Site-specific (*glmS* locus) Tn*7*-based insertion vector; *tnsABCD*, Km^R, Amp^R	René Uebe at UBT (unpublished)
pBAMII-Tn*7*-P_{cetBA2}-*cetBA*$_2$	Δ*cetBA*$_2$ transcomplementation plasmid; P_{cetBA2}-*cetBA*$_2$ was amplified with primers 551 & 552 from the wild-type background, cut with XhoI, and cloned into XhoI & SmaI digested pBAMII-Tn*7*	This study
pBAMII-Tn*7*-P_{cetBA2}-*cetB*$_2$	Δ*cetB*$_2$ transcomplementation plasmid; P_{cetBA2}-*cetB*$_2$ was amplified with primers 551 & 552 from the Δ*cetA*$_2$ strain, cut with XhoI, and cloned into XhoI & SmaI digested pBAMII-Tn*7*	This study
pBAMII-Tn*7*-P_{cetBA2}-*cetB*$_2$(N55D)	Tn*7*-based vector for the expression of CetB$_2$ with a N55D amino acid substitution in the FAD-binding pocket. To construct the plasmid, PCR products generated with primers 551 & 833 and 834 & 552 were combined via overlap extension PCR, digested with XhoI, and cloned into XhoI- and SmaI-digested pBAMII-Tn*7*. The resulting plasmid was introduced into the Δ*cetB*$_2$ strain.	This study
pBAMII-Tn*7*-P_{cetBA2}-*cetB*$_2$(F67Q)	Tn*7*-based vector for the expression of CetB$_2$ with a F67Q amino acid substitution in the FAD-binding pocket. To construct the plasmid, PCR	This study

	products generated with primers 551 & 841 and 842 & 552 were combined via overlap extension PCR, digested with XhoI, and cloned into XhoI- and SmaI-digested pBAMII-Tn*7*. The resulting plasmid was introduced into the Δ$cetB_2$ strain.	
pBAMII-Tn*7*-P_{cetBA2}-$cetB_2$(W71M)	Tn*7*-based vector for the expression of $CetB_2$ with a W71M amino acid substitution in the FAD-binding pocket. To construct the plasmid, PCR products generated with primers 551 & 835 and 836 & 552 were combined via overlap extension PCR, digested with XhoI, and cloned into XhoI- and SmaI-digested pBAMII-Tn*7*. The resulting plasmid was introduced into the Δ$cetB_2$ strain.	This study
pBAMII-Tn*7*-P_{cetBA2}-$cetB_2$(N86H)	Tn*7*-based vector for the expression of $CetB_2$ with a N86H amino acid substitution in the FAD-binding pocket. To construct the plasmid, PCR products generated with primers 551 & 837 and 838 & 552 were combined via overlap extension PCR, digested with XhoI, and cloned into XhoI- and SmaI-digested pBAMII-Tn*7*. The resulting plasmid was introduced into the Δ$cetB_2$ strain.	This study
pBAMII-Tn*7*-P_{cetBA2}-$cetA_2$	Δ$cetA_2$ transcomplementation plasmid; P_{cetBA2}-$cetA_2$ was amplified with primers 551 & 552 from the Δ$cetB_2$ strain, cut with XhoI, and cloned into XhoI & SmaI digested pBAMII-Tn*7*	This study
pBAMII-Tn*7*-P_{cetBA2}	control plasmid; P_{cetBA2} was amplified with primers 551 & 552 from the Δ$cetBA_2$ strain, cut with XhoI, and cloned into XhoI & SmaI digested pBAMII-Tn*7*	This study
pJH39	Tn*5* transposon vector for random single-copy chromosomal insertion; pBAM1 *ori*R6K, $P_{mamDC45}$-*mamC-maggbp-gbp*, Km^R, Amp^R, *tnpA*	[6]
pBAM-$P_{mamDC45}$-$cetA_2$-*omNG*	Tn*5*-based integrative plasmid for expression of $CetA_2$-mNG under control of the constitutive $P_{mamDC45}$ promoter; the genetic fusion was assembled as outlined in the Supporting Text and subsequently inserted into pJH39, which had been digested with HindIII and BamHI	This study
pBAM-$P_{mamDC45}$-*omNG*-$cetB_2$	Tn*5*-based integrative plasmid for expression of mNG-$CetB_2$ under control of the constitutive $P_{mamDC45}$ promoter; pJH39-based plasmid; refer to the Supporting Text for construction details	This study
pUT18C	BACTH vector designed to express a given polypeptide fused in frame at its N-terminal end with the T18 fragment; ColE1 ori; Amp^R	[36]
pUT18	BACTH vector designed to express a given polypeptide fused in frame at its C-terminal end with the T18 fragment; ColE1 ori; Amp^R	[36]

pKT25	BACTH vector designed to express a given polypeptide fused in frame at its N-terminal end with the T25 fragment; p15 ori; Km^R	[36]
pKNT25	BACTH vector designed to express a given polypeptide fused in frame at its C-terminal end with the T25 fragment; p15 ori; Km^R	[36]
pUT18C-zip; pKT25-zip	Derivatives of pUT18C and pKT25 with a 114 bp DNA fragment encoding for a leucine zipper (positive control for two-hybrid assays)	[36]
pUT18C-*cetB$_2$*	*cetB$_2$* amplified with primers 530 & 531 cloned into pUT18C via XbaI & KpnI	This study
pUT18-*cetB$_2$*	*cetB$_2$* amplified with primers 530 & 531 cloned into pUT18 via XbaI & KpnI	This study
pKT25-*cetB$_2$*	*cetB$_2$* amplified with primers 530 & 531 cloned into pKT25 via XbaI & KpnI	This study
pKNT25-*cetB$_2$*	*cetB$_2$* amplified with primers 530 & 531 cloned into pKNT25 via XbaI & KpnI	This study
pUT18C-*cetA$_2$*	*cetA$_2$* amplified with primers 532 & 533 cloned into pUT18C via XbaI & KpnI	This study
pUT18-*cetA$_2$*	*cetA$_2$* amplified with primers 532 & 533 cloned into pUT18 via XbaI & KpnI	This study
pKT25-*cetA$_2$*	*cetA$_2$* amplified with primers 532 & 533 cloned into pKT25 via XbaI & KpnI	This study
pKNT25-*cetA$_2$*	*cetA$_2$* amplified with primers 532 & 533 cloned into pKT25 via XbaI & KpnI	This study
pET28a	His-tag expression vector; Km^R	Novagen
pET28a-*cetB$_2$*	*cetB$_2$* amplified with primers 388 & 549 cloned into pET28a via NdeI & BamHI	This study

Table S5. Primers. Restriction sites used for cloning and single nucleotide substitutions are underlined. Start- and stop-codons are indicated in bold.

No.	Primer name	Sequence (5'-3')
Deletion & Transcomplementation		
74	flaA-del_up_fwd	CGCCGATCCACACGTCGCAGCC
75	flaA-del_up_rev	**TTA**ACGGAAGTCAGC**CAT**GGCACACCCCCTTCTGG
76	flaA-del_dw_fwd	**ATG**GCTGACTTCCGT**TAA**TCGGTAATGGTTTAAAGAATGG
77	flaA-del_dw_rev	TGAGGAACGACAAATCCACGGGCAAGC
120	MGR_0403-del_up_fwd	CTCCGTGTGCATTTGCCGTTACGC
121	MGR_0403-del_up_rev	**CTA**TCCCAGGACCCG**CAT**ACCCAACCCCATTCGC
122	MGR_0403-del_dw_fwd	**ATG**CGGGTCCTGGGA**TAG**AAAGGGGGAGGGGGGCC
123	MGR_0403-del_dw_rev	GGGTCCTTGACTTTCCCTGGCGGTCG
126	MGR_1492-del_up_fwd	GTTCTGCTGGACCGTGGACTTCCC
127	MGR_1492-del_up_rev	**CTA**TGCCGCAGAAAA**CAT**GACAGCCTCACATTCGCGC
128	MGR_1492-del_dw_fwd	**ATG**TTTTCTGCGGCA**TAG**AGCATTTTCACGCGAAGCG
129	MGR_1492-del_dw_rev	TTCCATTGGCGACAGCCTGGACCG
132	MGR_2868-del_up_fwd	CCGATTTCGTCGGTTTGGCCGAGCG
133	MGR_2868-del_up_rev	**TCA**CGACCGCAAGGC**CAT**CTTTTCCTCCAGGCAGATGC
134	MGR_2868-del_dw_fwd	**ATG**GCCTTGCGGTCG**TGA**TCGGTGGGGCTGGACTTGG
135	MGR_2868-del_dw_rev	AGCACCTGGGCGACTTCGATATTGG
239	cetBA1-del_up_fwd	ACGGGTCCGGTGCAACATCAACACG
240	cetBA1-del_up_rev	**TCA**GGCCGTCTGAAC**CAT**GTGTCGTCGATCTCAACAGG
241	cetBA1-del_dw_fwd	**ATG**GTTCAGACGGCC**TGA**TCCATGACCACCACAGTCTC
242	cetBA1-del_dw_rev	ATGCAGCTCTTCCTCGTCTTCCAGGG
245	cetBA2-del_up_fwd	AATTCGCACAAGGCCAAGCGGTCGG
246	cetBA2-del_up_rev	**CTA**TTGCATTCGTGC**CAT**ATCCCCCCCACGGGTCGGC
247	cetBA2-del_dw_fwd	**ATG**GCACGAATGCAA**TAG**GGCCGAAGGCCGGCCCGTG
248	cetBA2-del_dw_rev	ATGCTTTGCATGGTGTCGCGGTCGG
251	cetBA3-del_up_fwd	GCCGCGACCTTCCATCAGTTCCATCACCC
252	cetBA3-del_up_rev	**TCA**GAATGCTGCCGC**CAT**CCCACCCCCCCAAACC
253	cetBA3-del_dw_fwd	**ATG**GCGGCAGCATTC**TGA**CCTGCGCTAAAGATTAGGCACC
254	cetBA3-del_dw_rev	CGGCGGACGAGACGAAATCGGAGG
257	cetBA4-del_up_fwd	CCGGTGGACTTGGCCGTCATTGCCC
258	cetBA4-del_up_rev	**TCA**GCTCACACGATC**CAT**TGGTCATGCCCCGAAGGTCTTGG
259	cetBA4-del_dw_fwd	**ATG**GATCGTGTGAGC**TGA**ACAAAAAGCCCCGCCGAACAAACG

260	cetBA4-del_dw_rev	ACCATCACCGTCGCCCAGGACCAATCG
263	MGR_2126-del_up_fwd	GCTCGGTGATGACGCTGTGGGTGCC
264	MGR_2126-del_up_rev	**TCA**GCCGGCATCCAT**CAC**TTGAAAGCCCCCCCAGAGCC
265	MGR_2126-del_dw_fwd	**GTG**ATGGATGCCGGC**TGA**TCGGCCCGTCACGGGCC
266	MGR_2126-del_dw_rev	GCCGATGGCGAGAAGGCGTTGATGGG
269	MGR_3994-del_up_fwd	TCGACCAAACCGACGCAGGCGGACC
270	MGR_3994-del_up_rev	**TCA**GCCCAGGTCGGT**CAT**GCGGGCCTAATGGTTTAAGTGC
271	MGR_3994-del_dw_fwd	**ATG**ACCGACCTGGGC**TGA**GCCCGTTCCCGGCAAGC
272	MGR_3994-del_dw_rev	CTGCCCGGCTCCAACGCCACCACC
551	cetBA2-op_XhoI_fwd	GCCGCTCGAGAAGAATACCCGTGTGCAGTGCGAAATCC
552	cetBA2-op_KpnI_rev	GGGGTACCAAGGTACGGTCGGTCAGGAGGAAGG
587	cetB2-del_up_rev	CGAATTCATATCCCCCCCACGGGTCGGCTCG
588	cetB2-del_dw_fwd	GGGGGGGAT**ATG**AATTCGTCTTCGCTCTC**TAA**GG
589	cetB2-del_dw_rev	CGACGGTGGACGACTGGTCGC
592	cetA2-del_up_fwd	ATCCGTCATCAACGAGCCGACCC
593	cetA2-del_up_rev	**CTA**TTGCATC**TTA**GAGAGCGAAGACGAATT**CAT**CG
594	cetA2-del_dw_fwd	CTCTC**TAA**GATGCAA**TAG**GGCCGAAGGCCGGCCCGTG
KTS330	Part1_Del_MCP-3997_Rv	ACCACCTCTAGACAATGGGAAAGCGACACA
KTS331	Part1_Del_MCP-3997_Fw	GTCTCGTGC**ATG**TTCATCAAGATG**TAG**CAGGGCGTCAG
KTS332	Part2_Del_MCP-3997_Rv	GACGCCCTG**CTA**CATCTTGATGAA**CAT**GCACGAGACCC
KTS333	Part2_Del_MCP-3997_Fw	AAAAAACTCGAGCCAAGTTCCAACCCATCA
FP16	mgr3404upstr_rev	GGCACACCCCAAACATAAGTTTAC
FP58	mgr3404upstr_for2	TCTCAGGTGATGACCGTCGG
FP59	mgr3404dstr_for2	AAGTTAACTTTGCGTCGAAAACAGCGGGTG
FP66	mgr3404dstr_rev2	ATGAGCTCAGTTGCCCAGCAGGTTGAAGCG
**CetB$_2$ variants**		
833	cetB2_N55D_up_rev	GATGACATCGTGGGGCTGCCCCAGAACC
834	cetB2_N55D_dw_fwd	CCCCACGATGTCATCCGCCACCCGGACATGCC
835	cetB2_W71M_up_rev	GGTGTCCATCAACAGCTTGAATACGCAACG
836	cetB2_W71M_dw_fwd	CTGTTGATGGACACCATCGCCTCGGGCAAGG
837	cetB2_N86H_up_rev	GGAGCGGTGCACCACATAGGCGAAGCATTCC
838	cetB2_N86H_dw_fwd	GTGGTGCACCGCTCCAAGAACGGCGACC
841	cetB2_F67Q_up_rev	CAGCTTCTGTACGCAACGCGGCATGTCC
842	cetB2_F67Q_dw_fwd	TGCGTACAGAAGCTGTTGTGGGACACC

**Two-hybrid**		
530	cetB2_fwd_XbaI	GCTCTAGAC**ATG**GCACGAAGCGATGTTAAACTGACTGG
531	cetB2_rev_KpnI	GGGGTACCCCGAGAGCGAAGACGAATTCATCGTAGGGC
532	cetA2_fwd_XbaI	GCTCTAGAC**ATG**AATTCGTCTTCGCTCTCTAAGGCCTGG
533	cetA2_rev_KpnI	GGGGTACCCCTTGCATGCGCATACCCGAGCAGACG
**Fluorescent fusions**		
104	NheI_4HL_XbaI_long_fwd	GCTAGCCTGGCCGAAGCCGCGGCCAAGGAGGCCGCCGCGAA GGAAGCCGCGGCCAAGGAGGCCGCCGCGAAGGCCGCGGCCT CTAGA
113	MGR_1707_N_NheI-HL_rev	CTTCGGCCAGGCTAGCTTGCATGCGCATACCCGAGCAGACG
549	cetB2_BamHI_rev	CGGGATCC**TTA**GAGAGCGAAGACGAATTCATCGTAGGGC
630	cetA2_N_HindIII_fwd	CCCAAGCTTAGGAGATCAGCAT**ATG**AATTCGTCTTCGCTCTCTA AGGCCTGG
631	omNG10_XbaI-HL_fwd	GGCCGCGGCCTCTAGAGTGTCCAAGGGCGAGGAAGACAATATGG
632	omNG10_BamHI_rev	CGGGATCC**TCA**CTTGTACAGTTCGTCCATGCCC
642	cetB2_XbaI-HL_fwd	GGCCGCGGCCTCTAGAGCACGAAGCGATGTTAAACTGACTGG
650	omGL_HindIII-NdeI_fwd	CCCAAGCTTAGGAGATCAGCAT**ATG**GTCTCGAAGGGCGAGGAGC
652	omGL_NheI-HL_rev	CTTCGGCCAGGCTAGCCTTATACAGCTCGTCCATGTCATGG
666	omNG10_MP_fwd	CCTGCGAAGCTTAGGAGATCAGCATATGGTGTCCAAGGGCGAGG
667	omNG10_MP_rev	CCGCGGCTTCGGCCAGGCTAGCCTTGTACAGTTCGTCCATGCC
**Protein expression**		
388	cetB2_N_HindIII_NdeI_fwd	CCCAAGCTTAGGAGATCAGCAT**ATG**GCACGAAGCGATGTTAAA CTGACTGG
549	cetB2_BamHI_rev	CGGGATCC**TTA**GAGAGCGAAGACGAATTCATCGTAGGGC

Movie S1 (separate file). Time-lapse of aerotactic band formation (32x of original speed) in a microcapillary. Dark-field microscopy video recording (25 fps) at 100× magnification, focusing on wild-type cells in a zero field (geomagnetic field canceled to eliminate magnetic field-related effects on aerotactic band formation). The spatio-temporal dynamics of aerotactic band formation are orchestrated by the evolving oxygen gradient, influenced by oxygen diffusion into the medium, oxygen solubility, and oxygen consumption through aerobic respiration. The aerotactic band attains a quasi-stable position (steady state) when oxygen depletion resulting from cellular respiration equals the diffusion of oxygen into the medium.

Movie S2 (separate file). Dark-field microscopy video recording (25 fps) at 200× magnification capturing the aerotactic band (after 180 minutes) of wild-type, $\Delta cetBA_2$, and transcomplemented $\Delta cetBA_2$ cells in a zero field (geomagnetic field canceled). Cultures were adjusted to the same optical density before being transferred into glass microcapillaries. The meniscus at the air-liquid medium interface is directed towards the right.

Dataset S1 (separate file). Catalog of putative MCPs encoded in the *M. gryphiswaldense* genome, inclusive of protein size, locus_tags, predicted motifs/ sensory domains, domain architecture, MCP length class, and protein abundance. All proteins containing the 'MCPsignal' domain were classified as MCPs. 'n.a.' denotes data not available or not detected in the analysis data set.

SI References

[1] O. Raschdorf, J. M. Plitzko, D. Schüler, F. D. Müller, *Applied and environmental microbiology* **2014**, *80*, 4323.

[2] P. Richter, B. Melzer, F. D. Müller, *PLOS Genetics* **2023**, *19*, e1010788.

[3] C. J. Marx, M. E. Lidstrom, *BioTechniques* **2002**, *33*, 1062.

[4] A. Scheffel, A. Gärdes, K. Grünberg, G. Wanner, D. Schüler, *Journal of bacteriology* **2008**, *190*, 377.

[5] S. Borg, J. Hofmann, A. Pollithy, C. Lang, D. Schüler, *Applied and environmental microbiology* **2014**, *80*, 2609.

[6] S. Borg, F. Popp, J. Hofmann, H. Leonhardt, U. Rothbauer, D. Schüler, *mBio* **2015**, *6*, e02117-14.

[7] N. C. Shaner, G. G. Lambert, A. Chammas, Y. Ni, P. J. Cranfill, M. A. Baird, B. R. Sell, J. R. Allen, R. N. Day, M. Israelsson, M. W. Davidson, J. Wang, *Nat Methods* **2013**, *10*, 407.

[8] B. C. Campbell, E. M. Nabel, M. H. Murdock, C. Lao-Peregrin, P. Tsoulfas, M. G. Blackmore, F. S. Lee, C. Liston, H. Morishita, G. A. Petsko, *Proceedings of the National Academy of Sciences of the United States of America* **2020**, *117*, 30710.

[9] F. van den Ent, J. Löwe, *Journal of Biochemical and Biophysical Methods* **2006**, *67*, 67.

[10] U. Heyen, D. Schüler, *Applied microbiology and biotechnology* **2003**, *61*, 536.

[11] D. Schüler, R. Uhl, E. Bäuerlein, *FEMS microbiology letters* **1995**, *132*, 139.

[12] D. Pfeiffer, D. Schüler, *Applied and environmental microbiology* **2020**, *86*, e01976-19.

[13] M. K. Welleweerd, T. Hageman, M. Pichel, D. van As, H. Keizer, J. Hendrix, M. M. Micheal, I. S. M. Khalil, A. Mir, N. Korkmaz, R. Kräwinkel, D. M. Chevrier, D. Faivre, A. Fernandez-Castane, D. Pfeiffer, L. Abelmann, *The Review of scientific instruments* **2022**, *93*, 94101.

[14] A. Ducret, E. M. Quardokus, Y. V. Brun, *Nat Microbiol* **2016**, *1*, 16077.

[15] F. Popp, J. P. Armitage, D. Schüler, *Nature communications* **2014**, *5*, 5398.

[16] J. Schindelin, I. Arganda-Carreras, E. Frise, V. Kaynig, M. Longair, T. Pietzsch, S. Preibisch, C. Rueden, S. Saalfeld, B. Schmid, J.-Y. Tinevez, D. J. White, V. Hartenstein, K. Eliceiri, P. Tomancak, A. Cardona, *Nature methods* **2012**, *9*, 676.

[17] D. Pfeiffer, J. Herz, J. Schmiedel, F. Popp, D. Schüler, *Applied and environmental microbiology* **2020**, *87*, e02229-20.

[18] A. Kalmijn, *IEEE Trans. Magn.* **1981**, *17*, 1113.

[19] R. Nadkarni, S. Barkley, C. Fradin, *PloS one* **2013**, *8*, e82064.

[20] I. Arganda-Carreras, V. Kaynig, C. Rueden, K. W. Eliceiri, J. Schindelin, A. Cardona, H. Sebastian Seung, *Bioinformatics* **2017**, *33*, 2424.

[21] E. Katzmann, M. Eibauer, W. Lin, Y. Pan, J. M. Plitzko, D. Schüler, *Applied and environmental microbiology* **2013**, *79*, 7755.

[22] D. Pfeiffer, M. Toro-Nahuelpan, M. Bramkamp, J. M. Plitzko, D. Schüler, *mBio* **2019**, *10*, e02716-18.

[23] D. Pfeiffer, M. Toro-Nahuelpan, R. P. Awal, F.-D. Müller, M. Bramkamp, J. M. Plitzko, D. Schüler, *Proceedings of the National Academy of Sciences of the United States of America* **2020**, *117*, 32086.
[24] G. Karimova, A. Ullmann, D. Ladant, *Methods in enzymology* **2000**, *328*, 59.
[25] N. Philippe, L.-F. Wu, *Journal of molecular biology* **2010**, *400*, 309.
[26] X. Zhu, X. Ge, N. Li, L.-F. Wu, C. Luo, Q. Ouyang, Y. Tu, G. Chen, *Integrative biology quantitative biosciences from nano to macro* **2014**, *6*, 706.
[27] K. T. Silva, M. Schüler, F. Mickoleit, T. Zwiener, F. D. Müller, R. P. Awal, A. Weig, A. Brachmann, R. Uebe, D. Schüler, *mSystems* **2020**, *5*, e00565-20.
[28] Y. Li, E. Katzmann, S. Borg, D. Schüler, *Journal of bacteriology* **2012**, *194*, 4847.
[29] R. Uebe, D. Schüler, *Nature reviews. Microbiology* **2016**, *14*, 621.
[30] M. Grognot, K. M. Taute, *Current Opinion in Microbiology* **2021**, *61*, 73.
[31] A. Scheffel, D. Schüler, *Journal of bacteriology* **2007**, *189*, 6437.
[32] M. Bennet, A. McCarthy, D. Fix, M. R. Edwards, F. Repp, P. Vach, J. W. C. Dunlop, M. Sitti, G. S. Buller, S. Klumpp, D. Faivre, *PloS one* **2014**, *9*, e101150.
[33] C. T. Lefèvre, M. Bennet, L. Landau, P. Vach, D. Pignol, D. A. Bazylinski, R. B. Frankel, S. Klumpp, D. Faivre, *Biophysical journal* **2014**, *107*, 527.
[34] D. Hanahan, *Journal of molecular biology* **1983**, *166*, 557.
[35] D. Schultheiss, M. Kube, D. Schüler, *Applied and environmental microbiology* **2004**, *70*, 3624.
[36] G. Karimova, A. Ullmann, D. Ladant, *Journal of molecular microbiology and biotechnology* **2001**, *3*, 73.
